# National Borders Effectively Halt the Spread of Rabies: The Current Rabies Epidemic in China Is Dislocated from Cases in Neighboring Countries

**DOI:** 10.1371/journal.pntd.0002039

**Published:** 2013-01-31

**Authors:** Zhenyang Guo, Xiaoyan Tao, Cuiping Yin, Na Han, Jinning Yu, Hao Li, Haizhou Liu, Wei Fang, James Adams, Jun Wang, Guodong Liang, Qing Tang, Simon Rayner

**Affiliations:** 1 State Key Laboratory for Infectious Disease Prevention and Control, National Institute for Viral Disease Control and Prevention, Chinese Center for Disease Control and Prevention, Beijing, China; 2 State Key Laboratory for Virology, Wuhan Institute of Virology, Chinese Academy of Sciences, Wuhan, Hubei, China; Centers for Disease Control and Prevention, United States of America

## Abstract

China has seen a massive resurgence of rabies cases in the last 15 years with more than 25,000 human fatalities. Initial cases were reported in the southwest but are now reported in almost every province. There have been several phylogenetic investigations into the origin and spread of the virus within China but few reports investigating the impact of the epidemic on neighboring countries. We therefore collected nucleoprotein sequences from China and South East Asia and investigated their phylogenetic and phylogeographic relationship. Our results indicate that within South East Asia, isolates mainly cluster according to their geographic origin. We found evidence of sporadic exchange of strains between neighboring countries, but it appears that the major strain responsible for the current Chinese epidemic has not been exported. This suggests that national geographical boundaries and border controls are effective at halting the spread of rabies from China into adjacent regions. We further investigated the geographic structure of Chinese sequences and found that the current epidemic is dominated by variant strains that were likely present at low levels in previous domestic epidemics. We also identified epidemiological linkages between high incidence provinces consistent with observations based on surveillance data from human rabies cases.

## Author Summary

Rabies as a fatal zoonotic disease continues to be a public threat to global public health. After India, China reports the second highest number of human cases, with more than 117,500 deaths and three major epidemics since 1950. China remains in the middle of the third epidemic. In this work we investigate the impact of China on rabies in South East (SE) Asia. We collected nucleoprotein sequences from samples isolated throughout SE Asia and investigated their phylogenetic and geographic relationships. Our results indicate that clear geographic patterns exist within rabies virus in SE Asia, with isolates mainly clustered according to their geographic origin. While we found evidence of the sporadic exchange of strains between neighboring countries, the major strain responsible for the current Chinese epidemic does not appear to spread to neighboring countries. Our findings suggest that national geographical boundaries and border controls act as effective barriers to halt the spread of rabies from China into adjacent regions. We further investigated the geographic structure of Chinese sequences and found the current epidemic is dominated by variant strains that likely evolved from previous domestic epidemics. Our study provides valuable insight for rabies control and prevention in China and SE Asia.

## Introduction

Rabies is a fatal zoonotic disease, posing a severe public health problem with more than 55,000 human rabies deaths occurring annually. 99% of all fatalities occur in developing countries [1], [2] and Asia accounts for 80% of the worldwide total [3]. After India, China reports the second highest number of human cases, with more than 117500 recorded deaths since 1950 and three major epidemics (1956–1957, 1980–1990 and 1997 to the present day) [3], [4]. In the majority of cases in Asia, the domestic dog acts as the main reservoir for rabies transmission, with 85%–95% of human rabies cases ascribed to dog bites [5], [6] which in turn is a consequence of poor dog population control.

Rabies virus (RABV) belongs to the genus *Lyssavirus*, family *Rhabdoviridae*. Previous studies indicate that globally there are six distinct clades: Africa2, Africa3, Indian subcontinent, Arctic related, Cosmopolitan and Asian, with the last four lineages circulating in the Asia region [7], [8]. The Indian subcontinent clade is confined to Sri Lanka and southern India, while the arctic-related clade is widely distributed, spanning from far east Siberia to western Asia including India, Pakistan and Iraq [8]–[10]. The Asia clade is disseminated widely throughout Southeast Asian countries including China, Vietnam, Thailand, Cambodia, Philippines, Myanmar and Laos [7], [8].

In recent years, several studies involving phylogenetic analysis of RABVs have provided some insight into the evolutionary diversity of the rabies virus within China and the association with the strains in neighboring countries. In particular, previous findings indicate that RABVs from China are closely related to those from neighboring countries, possibly sharing a common ancestor [7], [11]–[14]. However, despite the severity of the problem, there has been no extensive investigation of the impact of the current rabies epidemic in China on surrounding regions or, conversely, the influence of these regions on the epidemic. Therefore, to investigate this question further we conducted a comprehensive phylogeographic analysis to explore the phylodynamics of rabies isolates from China and neighboring countries.

## Materials and Methods

### Sample collection and sequencing

From 2003 to 2010, we collected dog brain samples from provinces and municipalities in China where rabies was endemic or emerging, with regions selected as described previously [15]. Specifically, there were two stages to the surveillance program. In the first stage of the program the goal was to examine the infection rate in the general dog population in high incidence regions. In this stage, samples were collected from local meat markets. As dogs are brought to the meat market from the surrounding area (i.e. of the order of a few square kilometres) and as there is no transportation of dogs to other markets this represented a random sample of the dog population for this region. In the second stage of the surveillance program, isolates were primarily collected from suspected rabid animals (wildlife or domestic) or human related cases. All samples were tested for RABV using direct fluorescent assay (DFA) as described previously [6]. Total RNA was extracted by Trizol reagent (Invitrogen, Burlington, ON) according to the manufacturer's instructions. Based on this, 84 samples tested positive for rabies virus. Complete RABV N (nucleoprotein) gene sequences were determined using RT-PCR and sequencing reactions as described elsewhere [8], [16].

### Selection of GenBank sequences

**Figure 1:**
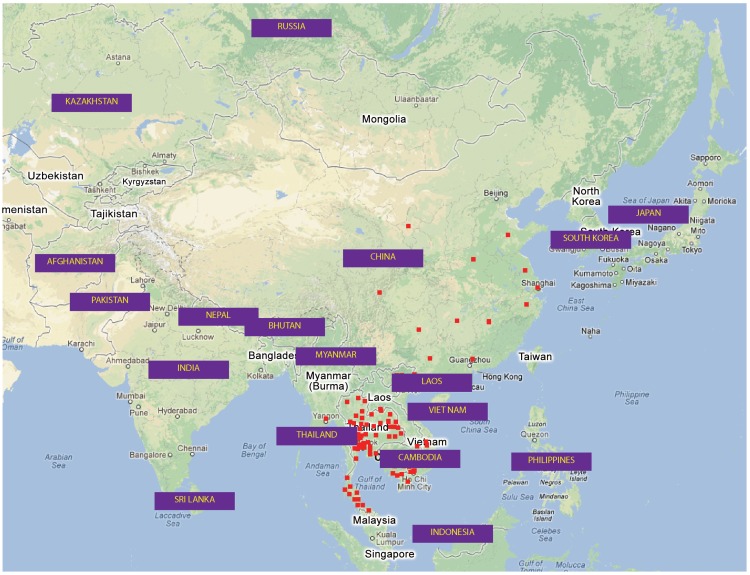
Location of isolates used in the study. Multiple datasets were generated based on different regions of the N gene and representation of each country varies amongst datasets. Squares show GPS coordinates of samples in datasets 4 & 5 used in the analysis of isolates from both sides of the South China border. China sequences only show the province in which the sample was isolated. Full details of each dataset are supplied in Table S1 and 2.

RABV sequences were collected from both China and neighboring Asian countries and, based on the N sequence, six different datasets were created that provided a compromise between number of sequences, alignment length and range of isolation date and geography. For the Asian phylogenetic analysis, dataset 1 comprised 110 sequences, spanning the full 1350 bp of the gene, dataset 2 consisted of 177 N sequences spanning nucleotides 1032–1350 and dataset 3 consisted of 312 sequences spanning nucleotides 64–399, two highly variable regions of the gene. Two additional datasets were created to investigate the relationship between isolates from China and countries close to its southern border. Dataset 4 comprised sequences spanning nucleotides 40–399 and dataset 5 contained sequences spanning nucleotides 1033–1329. Finally, to explore the phylogenetic diversity of RABVs in China, we retrieved all Chinese full length N sequences from Genbank. After combining with our newly acquired sequences and removing identical sequences from the same province, we composed a sixth dataset of 232 complete China N gene sequences (nucleotides 1–1353). A complete list of the new sequences and their background information, together with additional sequences retrieved from Genbank is given in Table S1 and the composition of the datasets are summarized in Table S2. A map of all geographic regions incorporated in the study and the geographical location of all isolates in datasets 4 and 5 (generated using the Google Maps API) is shown in Figure 1.

### Phylogenetic analysis of RABV in Asia

For each of the datasets 1–3, a maximum clade credibility (MCC) rooted tree was constructed using the Bayesian Markov Chain Monte Carlo (MCMC) methods implemented in the BEAST package (v1.6.2) [17]. A relaxed (uncorrelated lognormal) clock model, a general time-reversible nucleotide substitution model with rate heterogeneity and an invariable sites (GTR+I+Γ_4_) model of substitution determined by jModelTest [18], and a constant coalescent model were used to conduct the analysis. For each dataset, the MCMC analysis was run for 50 million generations to ensure sufficient mixing. Convergence of parameters estimates was checked using TRACER (http://beast.bio.ed.ac.uk/) and was indicated by an effective sample size (ESS)>200. From this approach we derived the phylogenies of each dataset. Posterior probability values were presented as indicators of nodal support.

### Quantifying the extent of geographic structure of RABV in Asia

To assess the geographic structure of RABV in Asia in a more quantitative manner, we examined the posterior distribution of genealogies within the trees produced in the previous step using the Bayesian Tip-Significance testing (BaTS) software tool [19]. For datasets 1 and 2, sequences were assigned uppercase letters to define their state according to their geographic location (See table 1 and table 2 for details). To determine the strength of geographical association with sampling locations across the entire tree, BaTS calculates the association index (AI) [20] and the parsimony score (PS) [21], as well as the maximum monophyletic clade size (MC) [19] to assess the correlation for specific locations. The PS score takes a score between 1 and n, where n is the number of tips in the tree; a PS of 1 corresponds to complete phylogeny-trait association (a measure of the extent to which neighboring taxa in a phylogenetic tree share a character of interest, in this case the geographical location). The AI is a sum across all internal nodes and is defined by
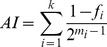
Where *f_i_* is the frequency of the most common trait (here the geographical location) among the tips subtended by node *i*, and *m_i_* is the number of tips subtended by *i*, Low AI values correspond to strong phylogeny-trait associations. The monophyletic clade (MC) statistic provides a measure of the phylogeny-trait correlation for each trait and is defined by
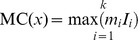
Where *m_i_* is the number of tips subtended by node *i* and *I_i_* = 1 if all tips under *i* have trait *x* and 0 otherwise. For this statistic, higher MC values indicate stronger phylogeny-trait associations.

**Table 1:** Phylogeny-trait analysis for Asian dataset 1.

*Statistic*	*Observed value (95% CI)*	*Null value (95% CI)*	*P value*
**AI**	0.87 (0.63–0.96)	8.75 (7.60–9.76)	0.00
**PS**	14.09 (14.00–15.00)	52.27 (49.24–54.95)	0.00
**MC(A)**	7.00 (7.00–7.00)	1.33 (1.00–2.00)	<0.001
**MC(B)**	5.00 (5.00–5.00)	1.06 (1.00–1.78)	<0.001
**MC(C)**	30.00 (30.00–30.00)	3.70 (2.19–5.48)	<0.001
**MC(D)**	1.00 (1.00–1.00)	1.01 (1.00–1.00)	1.00
**MC(E)**	7.00 (7.00–7.00)	1.47 (1.00–2.01)	<0.001
**MC(F)**	9.56 (9.00–11.00)	1.68 (1.00–2.68)	<0.001
**MC(G)**	4.00 (4.00–4.00)	1.07 (1.00–1.85)	<0.001

Table shows estimated geographic structure of RABVs using the Bayesian Tip-Significance testing (BaTS) software tool for Asian dataset 1 based on the estimated tree shown in figure 1a. Countries are assigned to the following states according to their geographic regions. A: Kazakhstan, Mongolia, Russia; B: South Korea; C: China; D: Japan; E: Afghanistan, India, Nepal, Pakistan, Sri Lanka; F: Cambodia, Laos, Myanmar, Thailand, Viet Nam; G: Philippines. Strength of geographical association for these locations across the entire tree is estimated by calculating the association index (AI) and the parsimony score (PS). Low AI and PS values correspond to strong phylogeny-trait associations. The correlation for each specific location is estimated by calculating the associated maximum monophyletic clade size (MC); larger MC values indicate stronger phylogeny-trait associations. The low AI and PS statistics indicate the isolates are mostly clustered according to their geographic origin. The large MC values (compared to the null value) indicate all the defined geographic regions exhibit population subdivision with the exception of region D (Japan) which indicates gene flow from other regions. See Materials and Methods for details.

**Table 2:** Phylogeny-trait analysis for Asian dataset 2.

*Statistic*	*Observed value (95% CI)*	*Null value (95% CI)*	*P value*
**AI**	2.05 (1.68–2.42)	18.89 (17.56–20.10)	0.00
**PS**	18.04 (18.00–19.00)	114.28 (109.84–118.27)	0.00
**MC(A)**	8.00 (8.00–8.00)	1.14 (1.00–1.99)	<0.001
**MC(B)**	8.99 (9.00–9.00)	1.10 (1.00–1.90)	<0.001
**MC(C)**	45.01 (45.00–45.00)	3.35 (2.50–4.53)	<0.001
**MC(D)**	2.00 (2.00–2.00)	1.01 (1.00–1.00)	0.002
**MC(E)**	23.34 (21.00–26.00)	2.44 (2.00–3.24)	<0.001
**MC(F)**	12.24 (7.00–16.00)	1.74 (1.09–2.25)	<0.001
**MC(G)**	8.00 (8.00–8.00)	1.09 (1.00–1.79)	<0.001
**MC(H)**	13.99 (14.00–14.00)	1.26 (1.00–2.00)	<0.001
**MC(I)**	8.00 (8.00–8.00)	1.09 (1.00–1.91)	<0.001

Table shows estimated geographic structure of RABVs using the Bayesian Tip-Significance testing (BaTS) software tool for Asian dataset 2 based on the estimated tree shown in figure 1b. See Countries are assigned to the following states according to their geographic regions. A: Kazakhstan, Mongolia, Russia; B: South Korea; C: China; D: Japan; E: Afghanistan, Bhutan, India, Nepal, Pakistan; F: Cambodia, Laos, Myanmar, Thailand, Viet Nam; G: Philippines; H: Indonesia; I : Sri Lanka. Strength of geographical association for these locations across the entire tree is estimated as described for Table 1. Materials and Methods for full details.

As BaTS performs the association test from the credible set of trees generated by BEAST in the previous step, it can also estimate the uncertainty associated with the predictions.

### RABV migration among Asian countries

The BaTS analysis indicated there was clustering of geographical states according to region and strong phylogenetic trait association, suggesting the possibility of the occurrence of translocation events. To test how the rabies virus was dispersed across the geographic region of Asia, each isolate was assigned the following lowercase letters to define their state according to their country of origin (a: Afghanistan; b: Cambodia; c: China; d: India; e: Indonesia; f: Japan; g: Kazakhstan; h: Laos; i: Mongolia; j: Myanmar; k: Nepal; l: Pakistan; m: Philippines; n: Russia; o: South Korea; p: Sri Lanka; q: Thailand; r: Vietnam) and RABV translocation events were traced through phylogenies derived from the Asian datasets utilizing the program MigraPhyla [22], [23] using both accelerated transformation of character states (ACCTRAN) and delayed transformation of character states (DELTRAN) parsimony optimization methods. To estimate the reliability of the predicted translocation events, a Monte Carlo test of 10,000 trials was used to randomly distribute the same localities across the tree tips and these ‘random’ trees were then examined for translocation events. The P value for a translocation event between two locations was estimated based on the number of times the translocation event was observed in the original tree compared to the number of times the events occurred in the ‘randomized’ trees. To correct for multiple tests and the sparsity of the generated translocation matrix, a sparse false discovery rate (sFDR) correction was applied to test the significance of the estimated P values. The sFDR cutoff was set by *P value rank*×(0.05/*total of migration events*)>*P value*. Translocation results were visualized using the Circos software package [24].

### Phylogenetic analysis of isolates close to south China border

To investigate the relationship between isolates from China and from regions close to the South China border, two ML trees based on datasets 4 and 5 respectively were constructed. The datasets were comprised of sequences from countries adjacent or close to the South China border (Figure 1). Sequences from India, Bhutan and Bangladesh were not included as these countries border Tibet and Sichuan which only began to record rabies cases in 2011. Two datasets were used because different surveillance programs in the various countries sequenced different regions of the N gene and it was not possible to generate a single comprehensive dataset representative of the entire geographic region. The datasets are summarized in Table S1 and S2.

### Phylogeography analysis of RABV in China

Using the same nucleotide substitution model as datasets 1 to 3, we used dataset 6 to reconstruct a MCC tree using BEAST v.1.6.2 [17]. As two major clades of Chinese isolates were identified in both the Asian and China analyses and accounted for most sequences in dataset 6, we selected these for further investigation. To determine the viral dispersion among provinces in China, a non-reversible discrete phylogeography model was applied to each of these two lineages, with the sampling provinces of these Chinese isolates acting as the discrete states [25]. As the geographic origin of *RABV* remains unclear, we used a Bayesian stochastic search variable selection (BSSVS) method which employed a Bayes factor test to identify the best supported migration pathways between geographic locations (i.e. provinces) that were epidemiologically linked [25]. The SPREAD program [26] was used to produce an animation of the results in the keyhole markup language (KML) to illustrate the epidemiological links, which can be viewed by Google Earth (http://earth.google.com).

## Results

### Phylogenetic structure of RABVs in Asia

**Figure 2:**
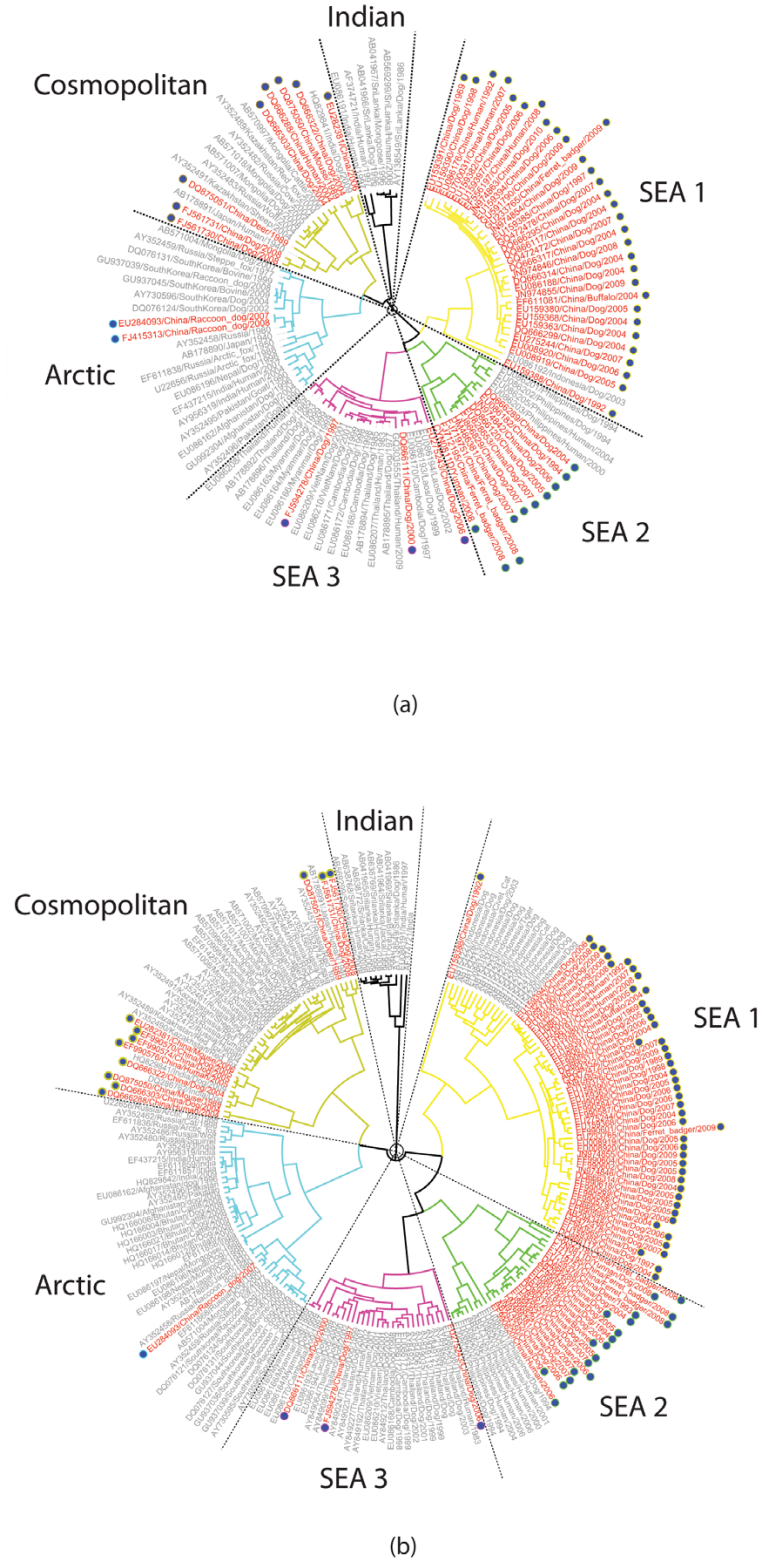
Estimated phylogenetic relationship of South East Asia isolates. Maximum clade credibility (MCC) tree for Asia dataset 1 (a) and dataset 2 (b). Isolates from China are marked in red and marked with solid circle; isolates from other countries are marked in gray. Six distinct clusters are supported with strong a posteriori probability values: Indian subcontinent; Cosmopolitan; Arctic-related; Southeast Asia (SEA) SEA1; SEA2; and SEA3.

Consistent with previous studies [7], [8], phylogenetic analysis of datasets 1, 2 and 3 revealed six distinct clusters in Asia: Indian subcontinent; Cosmopolitan; Arctic-related; Southeast Asia (SEA) SEA1; SEA2; and SEA3, all of which are supported with strong a posteriori probability values (Figure 2a, 2b and Figure S1). The geographic composition of these clades is also consistent with previous results. The Indian subcontinent cluster only contains isolates from India and Sri Lanka [8]. The Cosmopolitan cluster comprises isolates from a much broader region of Asia including Russia, Kazakhstan, Mongolia, China and India. Interestingly, an unpublished strain isolated from dog in Pantnagar in Uttarakhand Northern India (HQ829841) was grouped with isolates from China rather than with those from India, but lack of background information makes it difficult to determine the significance of this result. The Arctic-related cluster is comprised of strains circulating in Russia, Mongolia, South Korea, China, India, Nepal as well as Afghanistan and Pakistan, and other publications also report strains from Middle Eastern countries such as Iran and Iraq placed within this clade [9], [11]. The SEA1 cluster is confined to strains from China and Indonesia and there is clear subdivision according to geographic origin. The SEA2 cluster includes isolates from China and Philippines and is similarly split into two subgroups according to country of origin. The SEA3 cluster contains isolates from southwestern China and the biogeographical region referred to as the Indochina peninsula or approximately equivalent to Mainland Southeast Asia, similar to the Asian 2 group reported in another study [7]. Overall, our results agree with previous studies, but the structure within each clade offers new insight from a geographical and phylogenetic perspective. Despite the obvious correlation between China and neighboring countries, the distinct grouping of Chinese isolates suggest that the Chinese strains in the Cosmopolitan, SEA1 and SEA2 clusters, which contain the majority of the Chinese isolates, have evolved independently from their counterparts from neighboring countries, regardless of the collection date of isolates.

### Quantifying the extent of geographic structure of RABV in Asia

In our phylogeny-geographic origin association analysis, we grouped countries according to their geographic proximity and examined their dispersion within the predicted trees by calculating the PS and AI indices. The results for dataset 1 and dataset 2 are summarized in Table 1 and Table 2 respectively. For each dataset, a measure of the overall tree structure is provided by the AI and PS statistics; these can be interpreted by comparison with the associated null value, which is the corresponding statistic calculated from a null distribution of trees randomly selected from the posterior sample of trees generated by BEAST. In both cases, the AI and PS statistics for the estimated trees are much less than the null values at P = 0, indicating strong support for the presence of geographic structure and suggesting the isolates are mostly clustered according to their geographic origin. A measure of the phylogeny-trait association for each location is provided by the MC statistic and is calculated for each location in both datasets (Table 1 and Table 2). The MC statistic is positively correlated with the strength of phylogeny-trait association and values greater than the null value indicate strong association. All of the defined geographic regions show significant support (P value<0.001) for population subdivision with the exception of region D (Japan) which indicates gene flow from other regions. Region C only contains isolates from China, and the large MC value with significant statistical support indicates a preponderance of *in situ* evolution within China.

### Migration of RABV in Asia

**Figure 3:**
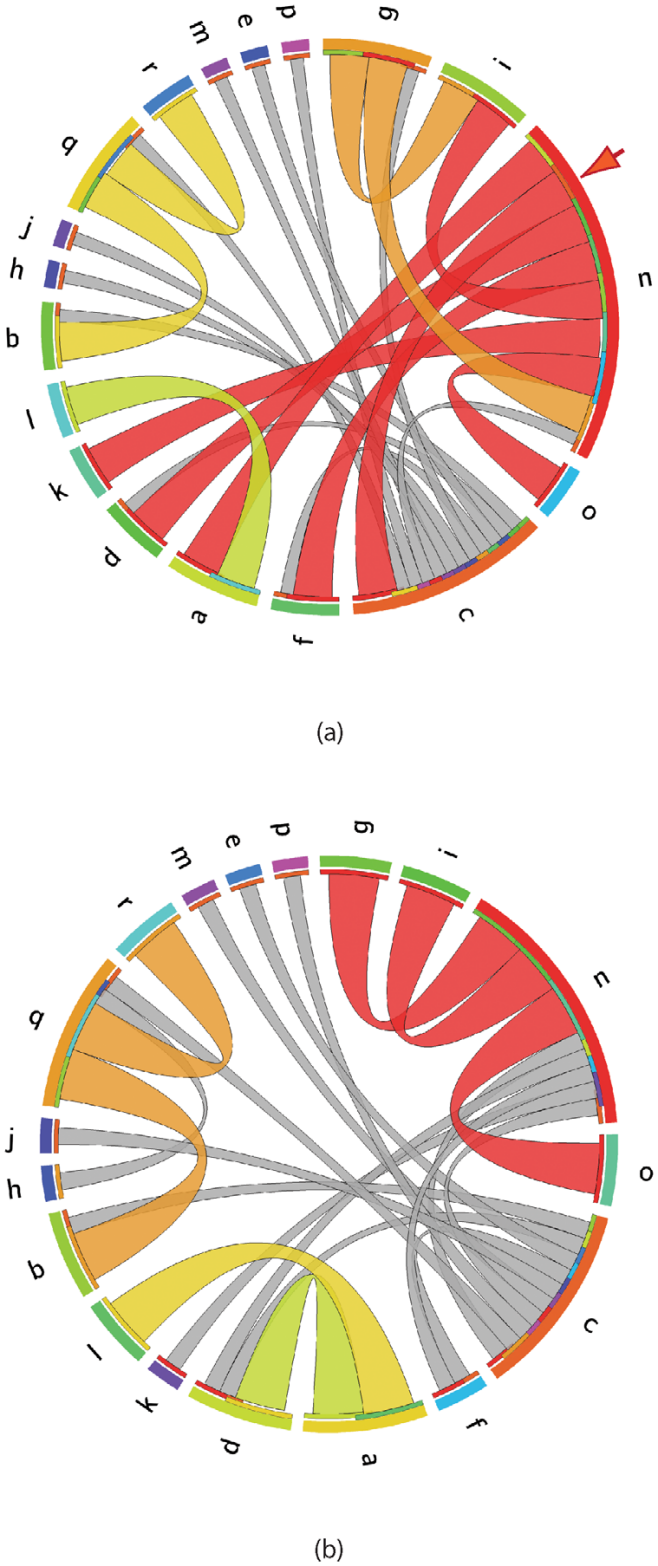
Predicted translocation events among South East Asia isolates. Estimated RABV translocation events among Asian countries for dataset 1 (a) and dataset 2 (b). Migration events between two countries are indicated by a line between those countries with the line coloured according to the source state. For example, the arrow in the top right of (a) marks a translocation event between Russia (state n - coloured red) and China (state c - coloured orange) The colour assigned to each state is indicated by the outer circle. The colour on the inner circle corresponds to the sink state for the translocation event. Translocation events that are not statistically significant are coloured grey. For example, there are many translocation events predicted with China as the source, but none of them are significant. Country states are defined as follows: a: Afghanistan; b: Cambodia; c: China; d: India; e: Indonesia; f: Japan; g: Kazakhstan; h: Laos; i: Mongolia; j: Myanmar; k: Nepal; l: Pakistan; m: Philippines; n: Russia; o: South Korea; p: Sri Lanka; q: Thailand; r: Vietnam.

The results of the MigraPhyla translocation analysis of the Asian datasets are summarized in Figure 3. After applying a sparse false discovery rate (sFDR) correction, the remaining translocation events inferred from the Asian datasets phylogenies indicate that China and Russia play an important role in transmitting RABVs across the Asian region. The following significant translocation pathways were identified (Figure 3a/dataset 1: Russia to Mongolia, South Korea, China, Japan, Afghanistan, India and Nepal; Kazakhstan to Mongolia and Russia; Afghanistan to Pakistan; and Thailand to Cambodia and Viet Nam; Figure 3b/dataset 2: Russia to South Korea, Kazakhstan and Mongolia; India to Afghanistan; Afghanistan to Pakistan; and Thailand to Cambodia and Viet Nam). Among these significant translocation events, dispersal mainly occurred among geographically adjoining countries in all three datasets (Figure 3 and Figure S2), with the exception of Russian isolates which, according to dataset 1, were predicted to have spread extensively to distant regions (Figure 3a). However, many countries in dataset 1 are represented by only a few isolates which may have biased the result; this conclusion is supported by the results for datasets 2 and 3 (Figure 3b and Figure S2) which, with the exception of South Korea, only retain translocation events from Russia to adjacent countries. However, these larger datasets still predict translocation events from China to every neighboring country (although these migration events do not have high statistical support) but relatively few predictions of translocation events in the opposite direction. i.e., China has an impact on rabies in neighboring countries, but cases imported into the country have negligible effect on the epidemic in China, which appears to be driven by internal events.

### Relationship between South East Asia and China isolates

**Figure 4:**
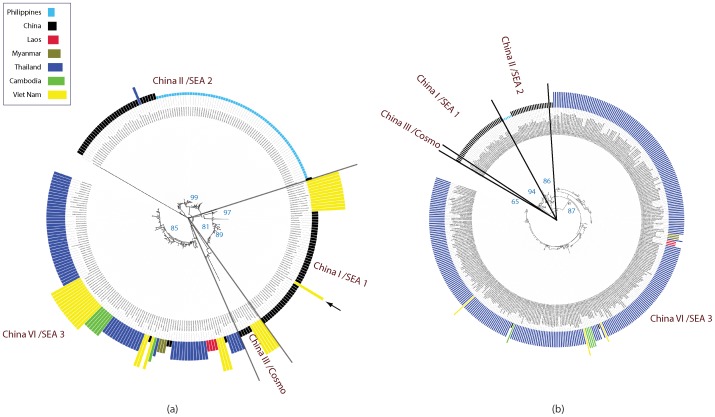
Estimated phylogenetic relationship of isolates close to south China border. Maximum Likelihood Trees for (a) dataset 4 and (b) dataset 5 containing sequences isolated in countries adjacent, or close to, the South China border. Outside bar indicates country of original according to colour and height of bar. In both trees, isolates form four major clades with strong bootstrap support. The majority of non Chinese isolates are placed in the China VI/SEA 3 and China II/SEA 2 clades. The China I/SEA 1 clade, which is the primary variant strain associated with the current epidemic in China, is exclusively composed of Chinese samples, with the exception of a small group of Vietnamese sequences (marked in yellow) in the left hand tree. These sequences form a separate branch with strong bootstrap support and the early branch point indicates these strains are distinct from the Chinese strains. The arrow marks a human sample from Viet Nam that is placed in the Chinese clade and which was isolated on the Vietnamese side of the principal border crossing into China. See main text for details.

To investigate the possibility that the absence of statistically significant translocation events between China and neighboring countries was simply a consequence of bias towards China isolates in the datasets, we generated two additional datasets, 4 and 5, comprising sequences from countries adjacent, or close to, the South China border. Surveillance data indicates that the border provinces of Guangxi, Guizhou, Guangdong and Henan in the Southwest represent the majority of early cases, so the majority of Chinese isolates were selected from these regions [27]. The ML trees for these two datasets are shown in Figure 4a and 4b respectively. The country of origin of the sequences are represented by the height and colour of the bars on the outside of the tree. In both trees the sequences are grouped into four major clades SEA1/China I, SEA2/China II, SEA3/China VI and Cosmopolitan/China III, consistent with their classification in the trees in Figure 2 and Figure S1. If the national borders failed to halt the spread of rabies, we would expect to find a close evolutionary relationship between China isolates from the current epidemic and isolates from other countries in South East Asia (Philippines, Laos, Myanmar, Thailand, Cambodia and Viet Nam).

Furthermore, surveillance studies indicate that SEA1 is the dominant strain in the current Chinese epidemic and SEA2 is associated with the previous epidemic that occurred during the 1970s and 1980s [27], [28] (and Table S3 and unpublished data). Thus, if there was any spillover from the current epidemic into neighboring countries, we would expect to find some isolates from other countries placed in the SEA1 clade. After removing duplicate entries, the two datasets represent a total of 550 unique isolates. The majority of these isolates are placed in SEA3, with the remainder dispersed between SEA2 and Cosmopolitan. A single group of 11 Viet Nam sequences isolated in the north of the country between 2007 and 2009 from both Human and Dog are located in the SEA1 (Figure 4a insert). However, their branch point from the China sequences, which include isolates dating back to 1969, indicates they are from a distinct lineage that is not associated with the current China epidemic. Interestingly, there is a single Viet Nam isolate placed in the middle of the Chinese China I sequences. Upon further investigation, it was found that this sequence was isolated from a human subject in Lang Son city in Lang Son province, which is the most important border crossing between Viet Nam and China, although no further information is available regarding the subject. Given this is a single Viet Nam isolate within the China branch, that all the Vietnamese dog isolates are in a separate branch, and considering the volume of cross border traffic between Lang Son and Pingxiang city (???) in Guizhou province on the Chinese side, it seems probable that this infection event occurred within China.

### Phylogeography analysis of RABVs in China

**Figure 5:**
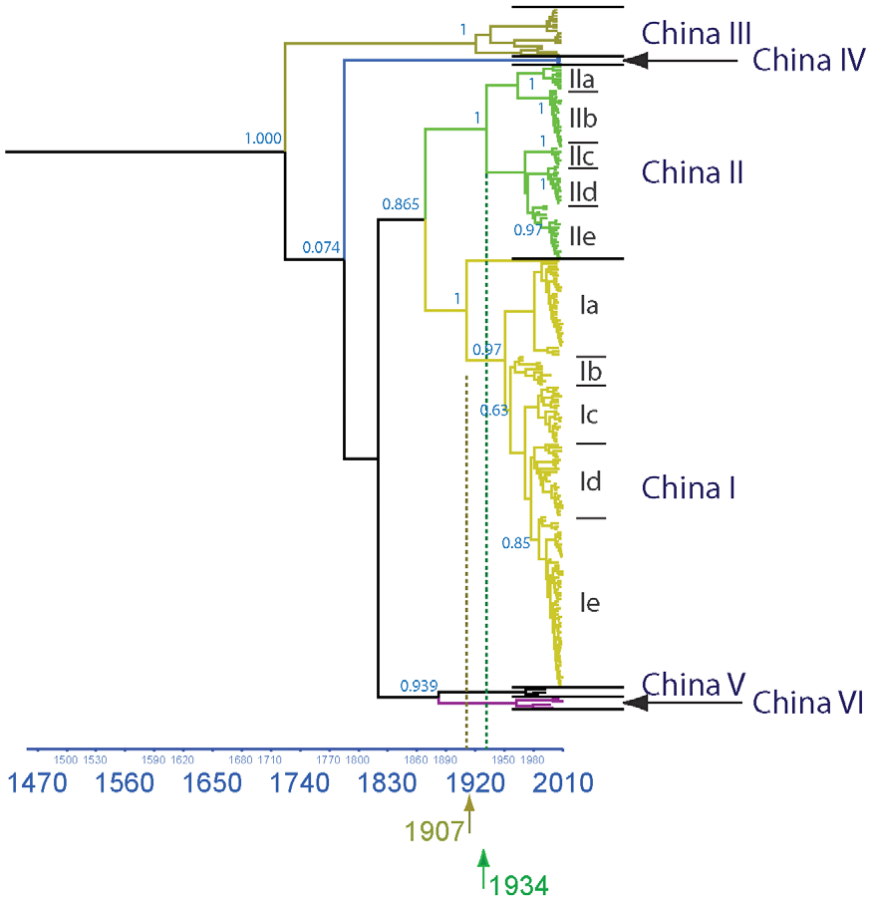
Estimated phylogenetic relationship of Chinese isolates. Maximum clade credibility (MCC) tree of dataset 6 using 232 full length N gene sequences of Chinese rabies virus isolates. Six distinct lineages (China I–VI) are predicted with high posterior value support with the majority of new cases placed in China I and China II. Estimated time of divergence for these two clades is shown on the X-axis. Horizontal branches are drawn according estimated year of divergence. Posterior probability values are shown for key nodes.

The above analyses indicate that SEA1, the dominant variant rabies strain in China, has not spilled over into neighboring countries. However, to further explore the diversity of the rabies virus in China, we conducted a comprehensive phylogenetic analysis using all available Chinese RABV N sequences (dataset 6). Bayesian coalescent analysis of RABVs from China identified six distinct lineages (China I–VI) with high posterior value support (Figure 5), which is in accordance with previous studies using complete G and N sequences [12]. The China_VI lineage includes a few isolates that originated from Guangxi and Yunnan provinces in southeastern China and which are closely related to RABVs from countries in the Indochina peninsula/Mainland Southeast Asia (corresponding to the SEA3 cluster in the Asian analysis results (Figure 2a and 2b). The China V lineage only contains three isolates that were collected around 20 years ago; this lineage is probably associated with an earlier epidemic (unpublished data - manuscript in preparation) and died out due to a population bottleneck or remains present at low levels and cannot be easily sampled. The China IV lineage consists of samples that are only found in Inner Mongolia and are closely related to the Arctic_related clade [9], [11] (Figure 2). The China III lineage has isolates collected across the country and corresponds to the Cosmopolitan clade [29]. The lack of diversity in this clade is highlighted by identification of four isolates in this clade possessing 99.4% nucleotide similarity despite being collected from four distant provinces (Guizhou, Hunan, Henan and Jiangsu) in the same year. The China I and China II lineages are representative of most RABV isolates prevalent in China over the last decade and correspond to the two Chinese subgroups samples in the SEA1 and SEA2 clusters in Figure 2. Both of these lineages can be further divided into five clearly defined sublineages with varying support. In several sublineages (China IIe, China Id and China Ie), recently acquired isolates shared a common ancestor with basal old strains from late 1980s or early 1990s, suggesting that those current prevalent RABV strains might evolved from earlier epidemic strains. Interestingly, 100% nucleotide identity was observed in eight new collected isolates (AH12/Anhui/2005, CGZ0518/Guizhou/2005, CGX0516/Guangxi/2005, CHN0813/Hunan/2008, CJS0621/Jiangsu/2006, CJX0902/Jiangxi/2009, CYN0924/Yunnan/2009, CZJ0804/Zhejiang/2008) from eight provinces spanning from 2005 to 2009 in the China_IIb sublineage.

In our Bayesian coalescent approach of China RABVs, the mean rate of nucleotide substitution was estimated to be 5.23×10^−4^ substitutions per site per year (95% HPD values, 3.94×10^−4^–6.68×10^−4^), which agrees with previous estimates [7], [30]. Estimates of the Time to the Most Recent Common Ancestor (TMRCA) indicates that current strains diverged around 1711 CE (95% HPD values, 1399–1869), concordant with previous estimation using N gene, but slightly earlier than the estimate for the G gene [7], [13], [31]. The divergence time of China I and China II lineages were determined to be 1907 and 1934, respectively, i.e., these two lineages evolved independently of external RABV strains over a long time span undergoing localized evolution.

**Figure 6:**
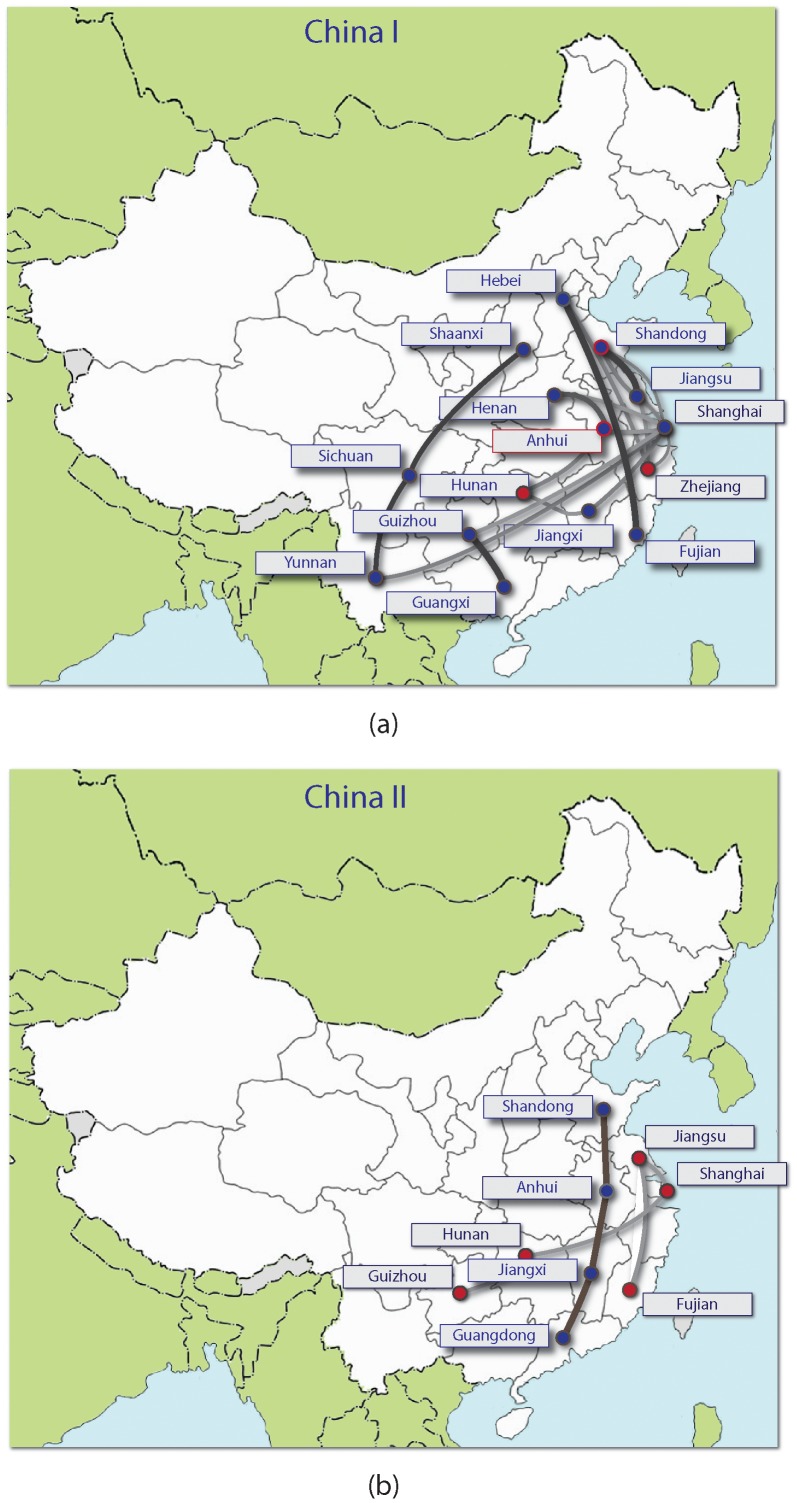
Estimated translocation events within China. Discrete phylogeographic analysis of MCC tree shown in figure 5 for clades (a) China I (b) China II, the two dominant variant strains in the current epidemic. Linkages significant at Bayes Factor 3 are shown. Lines link locations that are epidemiologically related with the shading and line thickness indicating relative support. Darker and thicker lines correspond to stronger support (e.g., Hebei to Fujian in China I), thinner and lighter lines indicate weaker support (e.g. Jiangsu to Fujian in China II).

The phylogeographical analyses of China I and China II lineages identified several provinces that appear to be epidemiologically linked. The transmission pathways for these two clades with Bayes factor greater than 3 are shown in Figure 6a and 6b respectively. Notably, China I contains many more linkages than China II, which suggests that this lineage plays the dominant role in the spread of rabies in China. Figure 6a also indicates that east China appears to be not only epidemiologically related to adjoining provinces but also to distant provinces, and seems to act as an epidemic hub for transmission of rabies virus to other regions, which is consistent with results from our previous analysis [15]. Other long distance transmissions of rabies virus can also be identified as well as translocation events between neighboring provinces. For example, Shaanxi province has previously experienced very low rabies incidence but cases have begun to increase in recent years. Figure 6a indicates a strong epidemiological linkage from Shaanxi to Sichuan and from Sichuan to Yunnan. This is consistent with surveillance data for human rabies cases which show dissemination of the virus from southwest China to neighboring provinces and into regions such as Shaanxi in the northern part of the county that have previously been incident free for several years [32]. For both clades, rather than a random dispersion of epidemiological linkages, there appears to be a general trend of vertical transmission (Shandong-Guangdong, Hebei-Fujian, Shandong-Zhejiang) and horizontal transmission (Yunnan-Shanghai, Guizhou-Shanghai, Hunan-Shanghai) which is also consistent with human rabies surveillance data which highlights a flow of cases from high incidence regions in the south of the country to medium and low incidence regions [32].

## Discussion

Rabies remains a serious public health problem throughout Asia. Nevertheless, the current goal is to eliminate rabies in China by 2020 (a target set at the ASEAN plus 3 rabies conference). Thus, effective and feasible long term programs for prevention and control are essential. Nevertheless, the situation can vary among countries or regions due to local problems or specific conditions [33], [34] and understanding these differences may aid the development of effective control measures.

In this study we performed a detailed phylogenetic analysis of RABVs in Asia using a comprehensive dataset selected from all currently available samples, as well as new samples collected as part of a national surveillance program, with a view to obtaining a better understanding of the role of different countries in the distribution of Asia rabies. There are already many published reports on the distribution of rabies within China and from the broader perspective of Asia. [6], [14], [35], [36]. These studies have investigated the phylogenetic relationship between Chinese strains and strains from other Asian countries and have demonstrated several isolates share a close phylogenetic relationship. However, the degree of exchange between neighboring countries and the relevance to the current rabies epidemic in China remains unclear. In our analysis we have examined the relationship between China and its Asian neighbors in far greater depth by investigating the geographical structure of the estimated phylogenies to try and interpret the contribution of specific regions to the observed epidemic in China, and conversely, the impact of the rabies epidemic in China on neighboring regions. One of the limitations of previous studies is the restricted number of samples that have been isolated from many Asian countries. In this work, we attempted to overcome this problem by constructing multiple datasets based on different regions of the N gene which allowed us to incorporate a broader range of isolates; the results for each dataset were consistent with those obtained with full gene sequences, indicating our results were robust.

Phylogenetic analysis indicates that geographic structure is the defining feature of the tree and that RABVs are strongly clustered according to their geographic origins. The Chinese isolates could be classified into two types: type A strains comprise isolates that were mixed in with strains from neighboring countries, indicating they shared a close evolutionary relationship; type B strains, although placed in clades with other Asian strains, formed distinct subclades that only consisted of Chinese sequences. For the type A isolates, the majority of Chinese isolates (from Guangxi and Yunnan provinces in Southwest of China) belonged to the SEA3 clade and clustered with isolates from countries in the Indochina peninsula/mainland Southeast Asia region, suggesting this might be a convergent region for RABV panmixis due to frequent commerce including animal trade [12], [37]. Additional Chinese RABV isolates from other clades were also clustered with isolates from other countries but their small number suggests these represented sporadic events. The majority of isolates were of type B strains. One major lineage of currently circulating Chinese rabies strains shared a common ancestor with those from Philippines in SEA2 clade, while a second Chinese lineage appears closely related to strains from Indonesia in SEA 1 clade. The estimated date of the TMRCA of these strains of 1907 and 1934 respectively, coincides with of historical emigration from China to Southeast Asian Countries [38], suggesting some association might exist between emigration and the transmission of RABVs between these countries [6], [35]. The presence of distinct clades implies that, after adapting to local hosts and environment, the Chinese RABV strains evolved separately, i.e. without gene flow in our out of the country, to become the predominant strains associated with the current rabies epidemic in China.

Translocation analysis of RABVs between China and other Asian countries illustrated that gene flow of RABV principally occurred amongst geographically adjacent countries. However, although several translocation events from China to other countries were predicted, all of them lacked strong statistical support. While the translocation analysis should be interpreted with caution due to possible sampling bias (due to the disproportionately high number of China isolates) and the analytical method (translocation events are based on best estimates of ancestor states which are not necessarily unique), our results are further supported by the classification of isolates from countries bordering south China. Thailand and Viet Nam in particular have comprehensive surveillance programs, but out of 550 isolates from the sampled countries, only 11 sequences (from Viet Nam) were grouped with Chinese sequences from the dominant variant strain, and these were in a separate and distinct branch. The identification of a single Viet Nam isolate within the Chinese sequences of China I clade is a cause for concern, although it does seem probable that the subject was infected within China. Nevertheless, it would be prudent to closely monitor the rabies situation in Lang Son city in Viet Nam, if similar cases were found in the canine population, then this would be evidence of spillover. Nevertheless, in spite of the scale of the epidemic within China, it appears that, currently, few cross border translocation events occur. Although China occupies a large geographic area bordering many countries, with the exception of arctic-related strains introduced from Russia to Inner Mongolia, there has been no major influx of rabies cases from outside China. Conversely, despite the large number of rabies cases currently experienced in China we identified relatively few translocation events. More importantly, these events originated from clades that are not significantly associated with the current epidemic.

Having established the current epidemic is evolving independently of neighboring countries, we also investigated the dispersion and genetic variation of the virus within China. The phylogeographic analysis of the Chinese RABV isolates dataset identified six lineages existing in China with an isolation date ranging from 1969 to 2010, spanning the current and previous epidemics [39]. Two major lineages (China I and China II) account for most of current rabies epidemic. Consistent with the Asian phylogeography analyses, these two lineages are highly localized, experiencing infrequent gene flow from outside mainland of China. The China III lineage corresponds to the Cosmopolitan clade, which had been predicted to a consequence of global colonization from Europe between the 15th to 19th century [40]. However, from the current dataset, it appears that this lineage only exists at a relatively low level and is associated with occasional events rather than significantly contributing towards the current epidemic. These three lineages can generally be classified as type B strains as described above. The remaining three lineages (China IV to VI) are more representative of type A strains and also, based on the total number and date of isolates, appear to contribute little to the current epidemic.

Previous studies have demonstrated the role of humans in dispersion of rabies in Africa [41], [42]. In particular, estimates of viral gene flow in localities in Algeria and Morocco in the Talbi study were 2 to 4 times higher than corresponding estimates in wildlife [42]. Although this highlights the importance of anthropogenic influences, it is difficult to make a direct comparison between our results and the Talbi study. Although the samples were collected over a 20 year period in Algeria and Morocco, there are marked socio-economic differences in the geographic regions. Firstly, the populations of Tunisia and Algeria are 10 and 35 million respectively, compared to 1.3 billion in China. Secondly, between 1995 and 2011, the estimated Gross Domestic Product (GDP) of Tunisia and Algeria increased from $18billion to $45billion and from $40billion to $180 billion respectively. Over the same time period, the GDP of China increased from $730 billion to $7.3 trillion. It is the rapid economical expansion in China over the last twenty years that has probably had the most significant impact on the spread of rabies and, ironically, on its control as more funds have become available for vaccination programs, education and subsidies for post exposure treatment. Prior to the plan for economic reform plan instigated by Deng XiaoPing, travel was more restricted and more commonly at the local level. Long distance travel was generally by train or bus and large scale transportation of goods only began to increase as the industrial infrastructure expanded. As the economy grew and relocation was more straightforward the population became more mobile. This likely facilitated the spread of RABV as people moved from villages to towns and cities, or between cities, transporting their dogs as part of the relocation process. Long distance relocation may explain our identification of identical N gene sequences from eight difference and geographically distant provinces. This is also supported by recent reports of rabies cases in Beijing where infected dogs were brought to the capital by migrant workers. On the other hand, dog meat markets likely aid the establishment and dissemination of RABV within a local region, as large numbers of dogs are able to roam freely. However, it is improbable they are associated with long range dissemination of the virus as, in general, there is no transportation of dogs occurs over long distances. News reports in the foreign press featuring truckloads of animals in cages are related to large scale operations that are only located in the major cities. Within each facility, dogs are kept within compounds or cages and moved to market in a matter of days, thus they are unable to contribute to the spread of the disease and only contribute sporadic cases.

In 1985, a national rabies control and prevention program was implemented, and by 1996 rabies cases had decreased to 159 [39], [43]. However, after this point the number of cases rapidly increased and a new epidemic emerged in the country. Available data showed that at least three distinct RABV lineages survived the control program and successfully reemerged, suggesting the presence of multiple reservoirs to allow RABVs' persistence over an extended period. In China, domestic dogs served as the main reservoir for RABVs with wildlife such as ferret badger also identified as a reservoir but playing an underdetermined role in the epidemic [44], [45]. Bats act as an additional potential reservoir of RABV [46] although their role has yet to be investigated in the current epidemic. All of these factors further complicate the task of rabies control and long-term support coordinated at the national level is key to the success of such efforts. Based on surveillance data and epidemiological surveys from the past decade, new regulations on rabies control have been drafted by the Ministry of Agriculture and Health in China. These new regulations place emphasis on rabies control at the source (such as vaccination of domestic animals, especially in rural area) and have already proved to be effective, as seen from the reduction in rabies cases in high incidence provinces in recent years [32]. Also, trial dog vaccination programs implemented in certain high incident regions in southwest provinces in China have also proved effective in controlling rabies. In the next phase of the program, vaccination will be extended to additional regions to incorporate more of the dog population with the aim of building up a vaccination barrier to combat rabies spread.

The rapid dispersal of rabies cases across the country indicates there are efficient transmission routes to facilitate dissemination of the virus. Evidence of the role of RABV transmission via human intervention and translocations has been well documented [8], [42], [47] and our predicted horizontal and vertical epidemiological linkages between provinces are consistent with the observed dispersion of the virus according to human rabies surveillance data [32].

Nevertheless, although there have been many reports regarding the recent spread of RABV across China, an detailed investigation of the impact of the epidemic in the context of Southeast Asia has yet to be considered. Our observation of a dominant variant strain that is unique to China is significant in that it suggests that neighboring countries have not been seriously impacted by the epidemic. In spite of the increasing trade between China and other countries in South East Asia, it further suggests that current border controls remain effective at restricting the passage of infected animals. The filtering of rabies cases at national borders shows that it is possible to limit the spread of the virus if suitable barriers exist and these findings may provide guidance for further determining effective measures for rabies control within China and to meet the goal of eliminating of rabies in China by 2020.

## Supporting Information

Figure S1 Maximum clade credibility (MCC) tree of Asian dataset 3.
(TIF)

Figure S2 Estimated RABV translocation events among Asian countries for dataset 4.
(TIF)

Table S1 Epidemiological information of rabies virus isolates in this study.
(DOC)

Table S2 Summary of datasets used in this study.
(DOC)

Table S3 Number of China I and China II isolates collected by Trial National Rabies Surveillance Program.
(DOC)

## References

[1] WHO (2005) WHO Expert Consultation on rabies. World Health Organ Tech Rep Ser 931: 1–88, back cover. 16485446

[2] Knobel DL, Cleaveland S, Coleman PG, Fevre EM, Meltzer MI, et al (2005) Re-evaluating the burden of rabies in Africa and Asia. Bull World Health Organ 83: 360–368. 15976877 PMC2626230

[3] Tang X, Luo M, Zhang S, Fooks AR, Hu R, et al (2005) Pivotal role of dogs in rabies transmission, China. Emerg Infect Dis 11: 1970–1972. 16485494 10.3201/eid1112.050271 PMC3367627

[4] Zhang YZ, Xiong CL, Lin XD, Zhou DJ, Jiang RJ, et al (2009) Genetic diversity of Chinese rabies viruses: evidence for the presence of two distinct clades in China. Infect Genet Evol 9: 87–96. 19041424 10.1016/j.meegid.2008.10.014

[5] Nadin-Davis SA, Sheen M, Wandeler AI (2011) Recent emergence of the Arctic rabies virus lineage. Virus Res 163: 352–62. 22100340 10.1016/j.virusres.2011.10.026

[6] Tao XY, Tang Q, Li H, Mo ZJ, Zhang H, et al (2009) Molecular epidemiology of rabies in Southern People's Republic of China. Emerg Infect Dis 15: 1192–1198. 19751579 10.3201/eid1508.081551 PMC2815963

[7] Meng S, Sun Y, Wu X, Tang J, Xu G, et al (2011) Evolutionary dynamics of rabies viruses highlights the importance of China rabies transmission in Asia. Virology 410: 403–409. 21195445 10.1016/j.virol.2010.12.011

[8] Bourhy H, Reynes JM, Dunham EJ, Dacheux L, Larrous F, et al (2008) The origin and phylogeography of dog rabies virus. J Gen Virol 89: 2673–2681. 18931062 10.1099/vir.0.2008/003913-0 PMC3326349

[9] Kuzmin IV, Hughes GJ, Botvinkin AD, Gribencha SG, Rupprecht CE (2008) Arctic and Arctic-like rabies viruses: distribution, phylogeny and evolutionary history. Epidemiol Infect 136: 509–519. 17599781 10.1017/S095026880700903X PMC2870842

[10] Nadin-Davis SA, Turner G, Paul JP, Madhusudana SN, Wandeler AI (2007) Emergence of Arctic-like rabies lineage in India. Emerg Infect Dis 13: 111–116. 17370523 10.3201/eid1301.060702 PMC2725804

[11] Shao XQ, Yan XJ, Luo GL, Zhang HL, Chai XL, et al (2011) Genetic evidence for domestic raccoon dog rabies caused by Arctic-like rabies virus in Inner Mongolia, China. Epidemiol Infect 139: 629–635. 20546631 10.1017/S0950268810001263

[12] Meng S, Xu G, Wu X, Lei Y, Yan J, et al (2010) Transmission dynamics of rabies in China over the last 40 years: 1969–2009. J Clin Virol 49: 47–52. 20650678 10.1016/j.jcv.2010.06.014

[13] Gong W, Jiang Y, Za Y, Zeng Z, Shao M, et al (2010) Temporal and spatial dynamics of rabies viruses in China and Southeast Asia. Virus Res 150: 111–118. 20214936 10.1016/j.virusres.2010.02.019

[14] Yamagata J, Ahmed K, Khawplod P, Mannen K, Xuyen DK, et al (2007) Molecular epidemiology of rabies in Vietnam. Microbiol Immunol 51: 833–840. 17895600 10.1111/j.1348-0421.2007.tb03979.x

[15] Jinning Yu HL, Tang Qing, Rayner Simon, Han Na, Guo Zhenyang, et al (2012) The Spatial and Temporal Dynamics of Rabies in China. PLoS Negl Trop Dis 6: e1640. 22563518 10.1371/journal.pntd.0001640 PMC3341336

[16] Kissi B, Tordo N, Bourhy H (1995) Genetic polymorphism in the rabies virus nucleoprotein gene. Virology 209: 526–537. 7778285 10.1006/viro.1995.1285

[17] Drummond AJ, Rambaut A (2007) BEAST: Bayesian evolutionary analysis by sampling trees. BMC Evol Biol 7: 214. 17996036 10.1186/1471-2148-7-214 PMC2247476

[18] Posada D (2008) jModelTest: phylogenetic model averaging. Mol Biol Evol 25: 1253–1256. 18397919 10.1093/molbev/msn083

[19] Parker J, Rambaut A, Pybus OG (2008) Correlating viral phenotypes with phylogeny: accounting for phylogenetic uncertainty. Infect Genet Evol 8: 239–246. 17921073 10.1016/j.meegid.2007.08.001

[20] Wang TH, Donaldson YK, Brettle RP, Bell JE, Simmonds P (2001) Identification of shared populations of human immunodeficiency virus type 1 infecting microglia and tissue macrophages outside the central nervous system. J Virol 75: 11686–11699. 11689650 10.1128/JVI.75.23.11686-11699.2001 PMC114755

[21] Slatkin M, Maddison WP (1989) A cladistic measure of gene flow inferred from the phylogenies of alleles. Genetics 123: 603–613. 2599370 10.1093/genetics/123.3.603 PMC1203833

[22] HoDac HM LR, Wallace RG, Fitch WM (2007) MigraPhyla: statistical analysis of migration events through a phylogeny. Version: 1.0b.

[23] Wallace RG, Hodac H, Lathrop RH, Fitch WM (2007) A statistical phylogeography of influenza A H5N1. Proc Natl Acad Sci U S A 104: 4473–4478. 17360548 10.1073/pnas.0700435104 PMC1838625

[24] Krzywinski M, Schein J, Birol I, Connors J, Gascoyne R, et al (2009) Circos: an information aesthetic for comparative genomics. Genome Res 19: 1639–1645. 19541911 10.1101/gr.092759.109 PMC2752132

[25] Lemey P, Rambaut A, Drummond AJ, Suchard MA (2009) Bayesian phylogeography finds its roots. PLoS Comput Biol 5: e1000520. 19779555 10.1371/journal.pcbi.1000520 PMC2740835

[26] Bielejec F, Rambaut A, Suchard MA, Lemey P (2011) SPREAD: spatial phylogenetic reconstruction of evolutionary dynamics. Bioinformatics 27: 2910–2912. 21911333 10.1093/bioinformatics/btr481 PMC3187652

[27] Yu J, Li H, Tang Q, Rayner S, Han N, et al (2012) The spatial and temporal dynamics of rabies in China. PLoS Negl Trop Dis 6: e1640. 22563518 10.1371/journal.pntd.0001640 PMC3341336

[28] Zhang YZ, Xiong CL, Xiao DL, Jiang RJ, Wang ZX, et al (2005) Human rabies in China. Emerg Infect Dis 11: 1983–1984. 16485502 10.3201/eid1112.040775 PMC3367615

[29] Badrane H, Tordo N (2001) Host switching in Lyssavirus history from the Chiroptera to the Carnivora orders. J Virol 75: 8096–8104. 11483755 10.1128/JVI.75.17.8096-8104.2001 PMC115054

[30] Talbi C, Holmes EC, de Benedictis P, Faye O, Nakoune E, et al (2009) Evolutionary history and dynamics of dog rabies virus in western and central Africa. J Gen Virol 90: 783–791. 19264663 10.1099/vir.0.007765-0

[31] Ming P, Yan J, Rayner S, Meng S, Xu G, et al (2010) A history estimate and evolutionary analysis of rabies virus variants in China. J Gen Virol 91: 759–764. 19889927 10.1099/vir.0.016436-0

[32] Yin CP, Zhou H, Wu H, Tao XY, Rayner S, et al (2012) Analysis on factors related to rabies epidemic in China from 2007–2011. Virol Sin 27: 132–143. 22492004 10.1007/s12250-012-3244-y PMC8218126

[33] Bourhy H, Dautry-Varsat A, Hotez PJ, Salomon J (2010) Rabies, still neglected after 125 years of vaccination. PLoS Negl Trop Dis 4: e839. 21152052 10.1371/journal.pntd.0000839 PMC2994912

[34] Dodet B, Goswami A, Gunasekera A, de Guzman F, Jamali S, et al (2008) Rabies awareness in eight Asian countries. Vaccine 26: 6344–6348. 18804507 10.1016/j.vaccine.2008.09.003

[35] Susetya H, Sugiyama M, Inagaki A, Ito N, Mudiarto G, et al (2008) Molecular epidemiology of rabies in Indonesia. Virus Res 135: 144–149. 18420300 10.1016/j.virusres.2008.03.001

[36] Smith J, McElhinney L, Parsons G, Brink N, Doherty T, et al (2003) Case report: rapid ante-mortem diagnosis of a human case of rabies imported into the UK from the Philippines. J Med Virol 69: 150–155. 12436491 10.1002/jmv.10253

[37] Liu Q, Xiong Y, Luo TR, Wei YC, Nan SJ, et al (2007) Molecular epidemiology of rabies in Guangxi Province, south of China. J Clin Virol 39: 295–303. 17588806 10.1016/j.jcv.2007.04.021

[38] Skeldon R (1996) Migration From China. Journal of International Affairs 49: 434–455.

[39] Hu R, Tang Q, Tang J, Fooks AR (2009) Rabies in china: an update. Vector Borne Zoonotic Dis 9: 1–12. 18803503 10.1089/vbz.2008.0046

[40] Nadin-Davis SAB, J. (2004) Europe as a source of rabies for the rest of the world. In: King AAF, Aubert M, Wandeler AI, editors. Historical perspective of rabies in Europe and the Mediterranean Basin: a testament to rabies by Dr Arthur A King, 2004. World Organisation for Animal Health. pp. 259–280.

[41] Hampson K, Dushoff J, Bingham J, Bruckner G, Ali YH, et al (2007) Synchronous cycles of domestic dog rabies in sub-Saharan Africa and the impact of control efforts. Proc Natl Acad Sci U S A 104: 7717–7722. 17452645 10.1073/pnas.0609122104 PMC1863501

[42] Talbi C, Lemey P, Suchard MA, Abdelatif E, Elharrak M, et al (2010) Phylodynamics and human-mediated dispersal of a zoonotic virus. PLoS Pathog 6: e1001166. 21060816 10.1371/journal.ppat.1001166 PMC2965766

[43] Song M, Tang Q, Wang DM, Mo ZJ, Guo SH, et al (2009) Epidemiological investigations of human rabies in China. BMC Infect Dis 9: 210. 20025742 10.1186/1471-2334-9-210 PMC2803182

[44] Liu Y, Zhang S, Wu X, Zhao J, Hou Y, et al (2010) Ferret badger rabies origin and its revisited importance as potential source of rabies transmission in Southeast China. BMC Infect Dis 10: 234. 20691095 10.1186/1471-2334-10-234 PMC2927599

[45] Zhang S, Tang Q, Wu X, Liu Y, Zhang F, et al (2009) Rabies in ferret badgers, southeastern China. Emerg Infect Dis 15: 946–949. 19523299 10.3201/eid1506.081485 PMC2727325

[46] Jiang Y, Wang L, Lu Z, Xuan H, Han X, et al (2010) Seroprevalence of rabies virus antibodies in bats from southern China. Vector Borne Zoonotic Dis 10: 177–181. 19492948 10.1089/vbz.2008.0212

[47] Johnson N, Black C, Smith J, Un H, McElhinney LM, et al (2003) Rabies emergence among foxes in Turkey. J Wildl Dis 39: 262–270. 12910752 10.7589/0090-3558-39.2.262

