# Supplementary figures and images for: National Borders Effectively Halt the Spread of Rabies: The Current Rabies Epidemic in China Is Dislocated from Cases in Neighboring Countries
Source: PLoS Negl Trop Dis. 2013 Jan 31;7(1):e2039. doi: 10.1371/journal.pntd.0002039 (PMC3561166; doi:10.1371/journal.pntd.0002039)

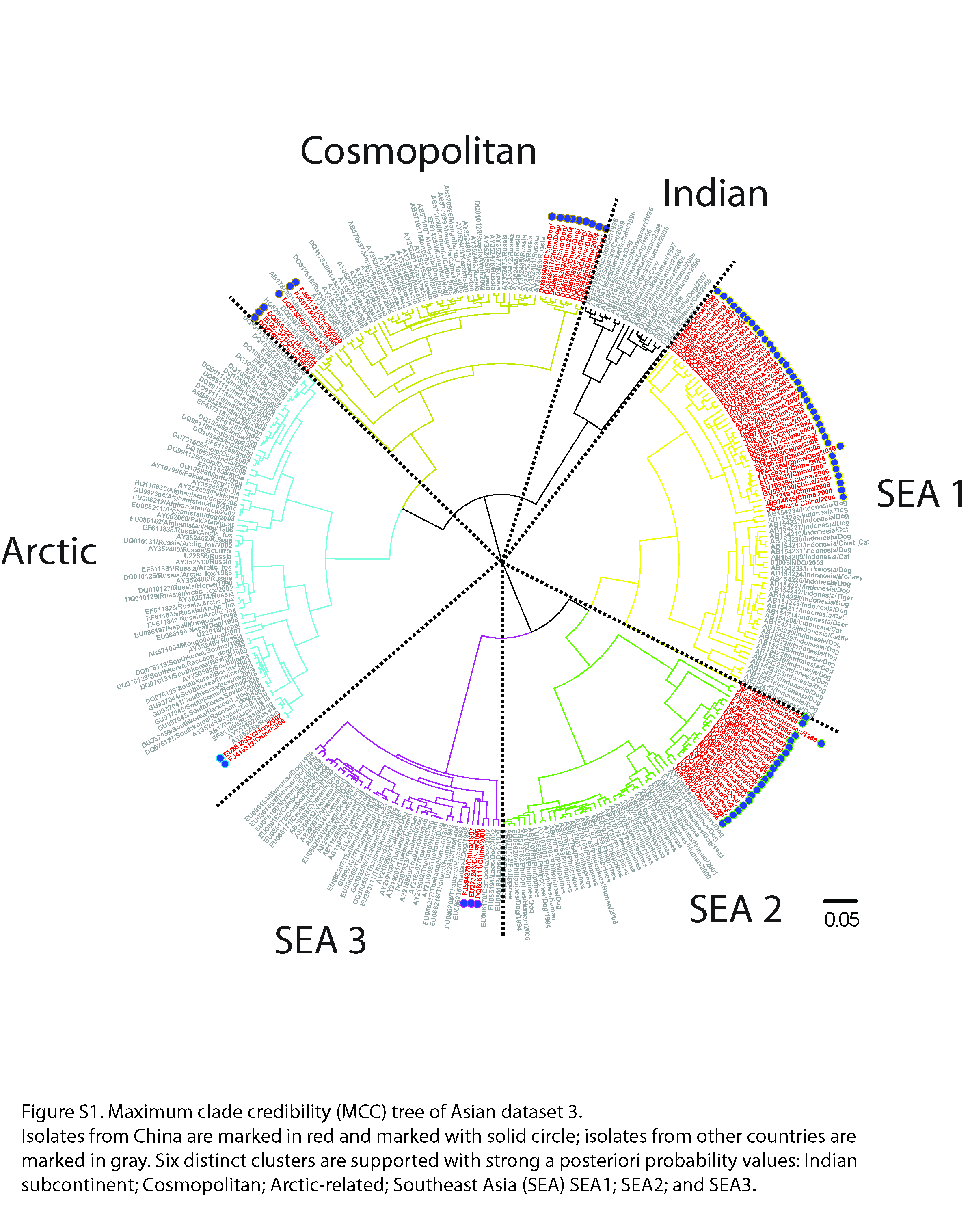

Supplement: Figure S1 — Maximum clade credibility (MCC) tree of Asian dataset 3. (TIF) [file pntd.0002039.s001.tif]

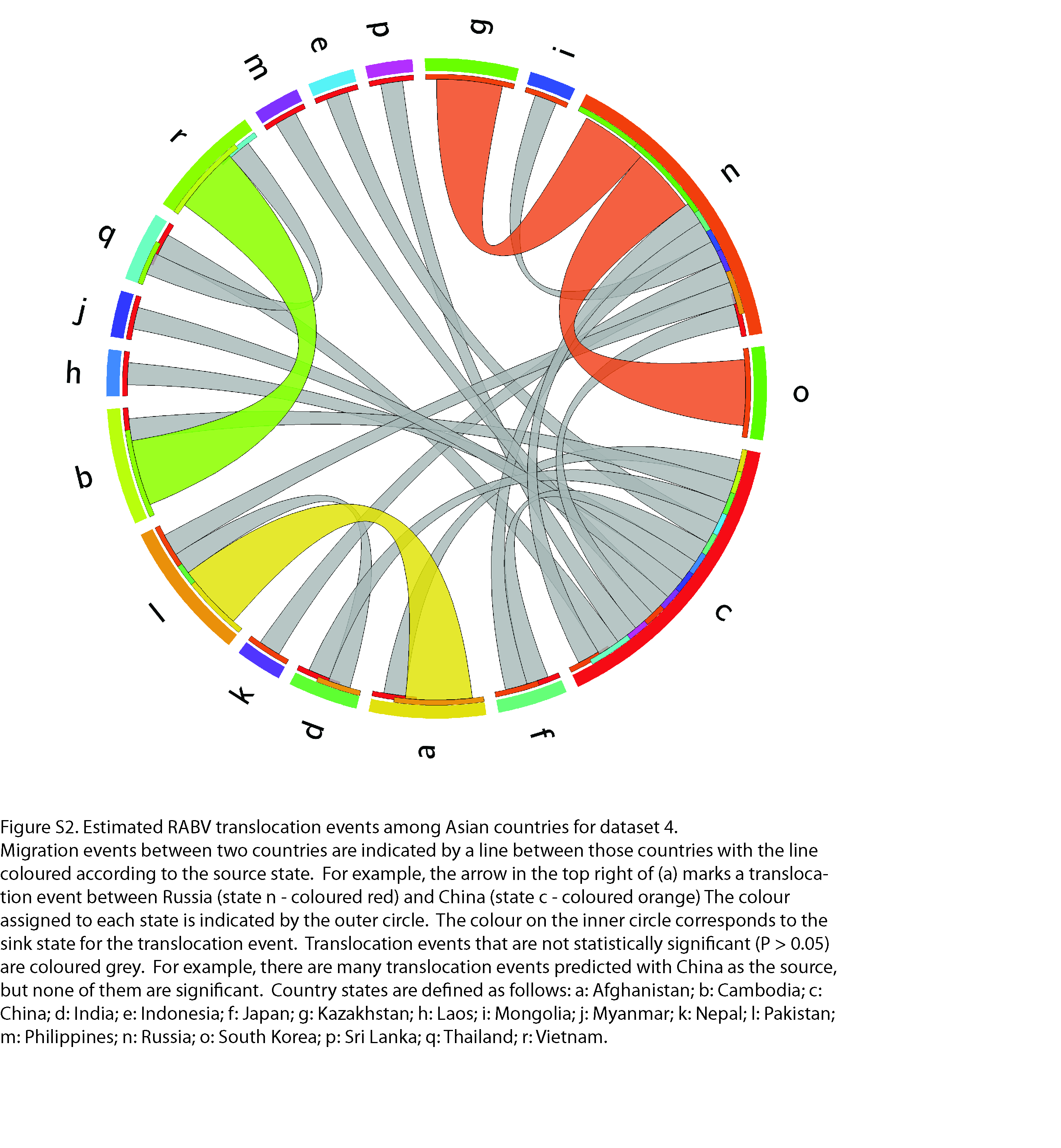

Supplement: Figure S2 — Estimated RABV translocation events among Asian countries for dataset 4. (TIF) [file pntd.0002039.s002.tif]
