# Supplementary material for: National Borders Effectively Halt the Spread of Rabies: The Current Rabies Epidemic in China Is Dislocated from Cases in Neighboring Countries
Source: PLoS Negl Trop Dis. 2013 Jan 31;7(1):e2039. doi: 10.1371/journal.pntd.0002039 (PMC3561166; doi:10.1371/journal.pntd.0002039)
Supplement: Table S1 — Epidemiological information of rabies virus isolates in this study. (DOC) [file pntd.0002039.s003.doc]

**Table S1. Epidemiological information of rabies virus isolates in this study.**

Table S1.1 Datasets 1,2,3, & 6

| Accession | Province/Country | Isolate name | Host | Collection time | Dataset | reference |
| --- | --- | --- | --- | --- | --- | --- |
| HM486360 | Anhui/China | DRV-AH08 | Dog | 2008 | 6 | This study |
| HM486362 | Anhui/China | FY13 | Dog | 2005 | 6 | This study |
| JN974823 | Guangdong/China | CGD0801D | Dog | 2008 | 6 | This study |
| HM486370 | Guangxi/China | CGX0516D | Dog | 2006 | 6 | This study |
| JN974824 | Guangxi/China | CGX0511D | Dog | 2006 | 6 | This study |
| JN974825 | Guangxi/China | CGX0601D | Dog | 2005 | 6 | This study |
| JN974826 | Guangxi/China | CGX0602D | Dog | 2008 | 6 | This study |
| JN974827 | Guangxi/China | CGX0606D | Dog | 2006 | 6 | This study |
| JN974828 | Guangxi/China | CGX0625D | Dog | 2006 | 6 | This study |
| JN974829 | Guangxi/China | CGX0801D | Dog | 2006 | 6 | This study |
| HM486368 | Guizhou/China | CGZ0508D | Dog | 2005 | 6 | This study |
| HM486369 | Guizhou/China | CGZ0620D | Dog | 2006 | 6 | This study |
| JN974830 | Guizhou/China | CGZ0501D | Dog | 2005 | 6 | This study |
| JN974831 | Guizhou/China | CGZ0518H | Human | 2010 | 6 | This study |
| JN974832 | Guizhou/China | CGZ0924H | Human | 2010 | 6 | This study |
| JN974833 | Guizhou/China | CGZ1030D | Dog | 2009 | 6 | This study |
| JN974834 | Guizhou/China | CGZ1031D | Dog | 2005 | 6 | This study |
| JN974835 | Hebei/China | CHB1007H | Human | 2010 | 6 | This study |
| JN974836 | Hunan/China | CHN0503D | Dog | 2008 | 6 | This study |
| JN974837 | Hunan/China | CHN0504D | Dog | 2008 | 6 | This study |
| JN974838 | Hunan/China | CHN0527D | Dog | 2006 | 6 | This study |
| JN974839 | Hunan/China | CHN0532D | Dog | 2006 | 6 | This study |
| JN974840 | Hunan/China | CHN0602D | Dog | 2006 | 6 | This study |
| JN974841 | Hunan/China | CHN0610H | Human | 2005 | 6 | This study |
| JN974842 | Hunan/China | CHN0633D | Dog | 2006 | 1,2,3,6 | This study |
| JN974843 | Hunan/China | CHN0635H | Human | 2008 | 6 | This study |
| JN974844 | Hunan/China | CHN0701D | Dog | 2009 | 6 | This study |
| JN974845 | Hunan/China | CHN0801D | Dog | 2005 | 6 | This study |
| JN974846 | Hunan/China | CHN0809D | Dog | 2007 | 1,2,3,6 | This study |
| JN974847 | Hunan/China | CHN0813H | Human | 2005 | 6 | This study |
| JN974848 | Hunan/China | CHN0903D | Dog | 2005 | 6 | This study |
| HM486348 | Jiangsu/China | CJS0523D | Dog | 2005 | 6 | This study |
| HM486350 | Jiangsu/China | CJS0622D | Dog | 2006 | 6 | This study |
| HM486351 | Jiangsu/China | CJS0629D | Dog | 2006 | 6 | This study |
| HM486353 | Jiangsu/China | CJS0538D | Dog | 2005 | 6 | This study |
| HM486363 | Jiangsu/China | CJS0634D | Dog | 2006 | 6 | This study |
| HM486364 | Jiangsu/China | CJS0621D | Dog | 2006 | 6 | This study |
| JN974849 | Jiangsu/China | CJS0840H | Human | 2008 | 6 | This study |
| JN974850 | Jiangsu/China | CJS0841D | Dog | 2008 | 6 | This study |
| JN974851 | Jiangsu/China | CJS0843D | Dog | 2008 | 6 | This study |
| JN974852 | Jiangsu/China | CJS0847D | Dog | 2008 | 6 | This study |
| JN974853 | Jiangxi/China | CJX0901D | Dog | 2009 | 1,2,3,6 | This study |
| JN974854 | Jiangxi/China | CJX0902D | Dog | 2009 | 6 | This study |
| JN974855 | Jiangxi/China | CJX0903D | Dog | 2009 | 1,2,3,6 | This study |
| JN974876 | Shaanxi/China | CSX0903D | Dog | 2009 | 6 | This study |
| HM486376 | Shandong/China | CSD0801D | Dog | 2008 | 6 | This study |
| HM486377 | Shandong/China | CSD0803D | Dog | 2008 | 6 | This study |
| HM486378 | Shandong/China | CSD0711D | Dog | 2007 | 6 | This study |
| HM486380 | Shandong/China | CSD0614D | Dog | 2006 | 6 | This study |
| HM486381 | Shandong/China | CSD0709D | Dog | 2007 | 1,2,3,6 | This study |
| JN974864 | Shandong/China | CSD0837D | Dog | 2009 | 6 | This study |
| JN974865 | Shandong/China | CSD0840D | Dog | 2009 | 6 | This study |
| JN974866 | Shandong/China | CSD0931D | Dog | 2009 | 6 | This study |
| JN974867 | Shandong/China | CSD0932D | Dog | 2009 | 6 | This study |
| JN974868 | Shandong/China | CSD0934D | Dog | 2008 | 6 | This study |
| JN974869 | Shandong/China | CSD0935D | Dog | 2008 | 6 | This study |
| HM486356 | Shanghai/China | CSH0419D | Dog | 2004 | 6 | This study |
| JN974870 | Shanghai/China | CSH0327D | Dog | 2003 | 6 | This study |
| JN974871 | Shanghai/China | CSH0328D | Dog | 2003 | 6 | This study |
| JN974872 | Shanghai/China | CSH0329D | Dog | 2004 | 6 | This study |
| JN974873 | Shanghai/China | CSH0415D | Dog | 2003 | 6 | This study |
| JN974874 | Shanghai/China | CSH0501D | Dog | 2005 | 6 | This study |
| JN974875 | Shanghai/China | CSH0504D | Dog | 2005 | 6 | This study |
| JN974856 | Sichuan/China | CSC0801D | Dog | 2010 | 6 | This study |
| JN974857 | Sichuan/China | CSC0808D | Dog | 2008 | 6 | This study |
| JN974858 | Sichuan/China | CSC0909D | Dog | 2009 | 6 | This study |
| JN974859 | Sichuan/China | CSC0910D | Dog | 2009 | 6 | This study |
| JN974860 | Sichuan/China | CSC0912D | Dog | 2008 | 6 | This study |
| JN974861 | Sichuan/China | CSC1013D | Dog | 2009 | 6 | This study |
| JN974862 | Sichuan/China | CSC1014D | Dog | 2010 | 6 | This study |
| JN974863 | Sichuan/China | CSC1016D | Dog | 2010 | 1,2,3,6 | This study |
| JF819605 | Yunnan/China | CYN0811D | Dog | 2008 | 1,2,3,6 | This study |
| JF819615 | Yunnan/China | CYN0814D | Dog | 2008 | 6 | This study |
| JF819611 | Yunnan/China | CYN0919D | Dog | 2009 | 6 | This study |
| JF819612 | Yunnan/China | CYN0924H | Human | 2009 | 6 | This study |
| JF819619 | Yunnan/China | CYN1003C | Cow | 2010 | 6 | This study |
| JF819623 | Yunnan/China | CYN1006D | Dog | 2010 | 6 | This study |
| JQ739682 | Yunnan/China | CYN1009D | Dog | 2010 | 6 | This study |
| JF819614 | Yunnan/China | CYN0921HN | Dog | 2009 | 6 | This study |
| JQ040596 | Yunnan/China | CYN1025H | Dog | 2009 | 6 | This study |
| CZJ0804D | Zhejiang/China | CZJ0804D | Dog | 2008 | 6 | This study |
| CZJ0805D | Zhejiang/China | CZJ0805D | Dog | 2008 | 6 | This study |
| JN974877 | Zhejiang/China | CZJ0803F | Ferret badger | 2008 | 6 | This study |
| JN974878 | Zhejiang/China | CZJ0814D | Dog | 2008 | 6 | This study |

| HQ116830 | Afghanistan | 20277 | Dog | 2005 | 3 |  |
| --- | --- | --- | --- | --- | --- | --- |
| EU086162 | Afghanistan | 04027AFG | Dog | 1996 | 1,2,3 |  |
| EU086211 | Afghanistan | 04029AFG | Dog | 2004 | 3 |  |
| EU086212 | Afghanistan | 02052AFG | Dog | 2002 | 3 |  |
| GU992304 | Afghanistan | 04035AFG | Dog | 2004 | 1,2,3 | Unpublished |
| HQ166003 | Bhutan | BHT2685 | Cattle | 2008 | 2 |  |
| HQ166004 | Bhutan | BHT88 | Dog | 2008 | 2 |  |
| HQ166006 | Bhutan | BHT187 | Cattle | 2008 | 2 |  |
| HQ166012 | Bhutan | BHT2 |  | 2008 | 2 |  |
| HQ166014 | Bhutan | BHT2000 | Dog | 2009 | 2 |  |
| HQ166017 | Bhutan | BHT2196 | Cattle | 2009 | 2 |  |
| HQ166021 | Bhutan | BHT68 | Dog | 2009 | 2 |  |
| EU086168 | Cambodia | 9911CBG | Dog | 1998 | 1,2,3 |  |
| EU086170 | Cambodia | 9914CBG | Dog | 1997 | 1,2,3 |  |
| EU086171 | Cambodia | 9916CBG | Dog | 1999 | 1,2,3 |  |
| EU086172 | Cambodia | 02006CBG | Dog | 1998 | 1,2,3 |  |
| HQ118104 | China | HeF | Dog | 2005 | 4 |  |
| EU086177 | China | 02041CHI | Dog | 2005 | 4 |  |
| EU086178 | China | 02042CHI | Dog | 2004 | 4 |  |
| EU086180 | China | 02044CHI | Dog | 2004 | 4 |  |
| EU086187 | China | 05006CHI | Dog | 2004 | 4 |  |
| EU159362 | China | FY1 | Dog | 2004 | 4 | Unpublished |
| EU159363 | China | FY2 | Dog | 2004 | 1,2,3,4 | Unpublished |
| EU159364 | China | FY3 | Dog | 2004 | 4 | Unpublished |
| EU159365 | China | FY5 | Dog | 2004 | 4 | Unpublished |
| EU159367 | China | FY9 | Dog | 2004 | 4 | Unpublished |
| EU159368 | China | FY6 | Dog | 2004 | 1,2,3,4 | Unpublished |
| EU159371 | China | FY10 | Dog | 2004 | 4 | Unpublished |
| EU159372 | China | FY12 | Dog | 2005 | 4 | Unpublished |
| EU159373 | China | FY13 | Dog | 2004 | 4 | Unpublished |
| EU159375 | China | FY15 | Dog | 2005 | 4 | Unpublished |
| EU159391 | China | H69 | Dog | 1969 | 1,2,4 | Unpublished |
| EU159396 | China | H | Dog | 1989 | 4 | Unpublished |
| EU159400 | China | H89 | Dog | 1989 | 4 | Unpublished |
| EU159401 | China | WJ | Dog | 2004 | 4 | Unpublished |
| HQ450385 | China | DRV-AH08 | Dog | 2004 | 4 | Unpublished |
| EU159379 | China | FEIDONG | Dog | 1989 | 2 | Unpublished |
| EU700031 | China | BeijingHu1 | Human | 2007 | 4 |  |
| JF441064 | China | SXTYD01 | Dog | 2010 | 3 |  |
| AY102998 | China | Rv338 | Cow |  | 3 |  |
| AY102999 | China | Rv341 | Dog |  | 3 |  |
| EU086214 | China | 9709CHI | Human | 1986 | 3 |  |
| AB294350 | China | CHdg4 | Dog |  | 2 | Unpublished |
| AB294351 | China | CHdg5 | Dog |  | 2 | Unpublished |
| AB294355 | China | CHbv9 | Bovine |  | 2 | Unpublished |
| FJ032313 | China | D01 | Dog | 2008 | 2 | Unpublished |
| FJ032318 | China | F01 | Ferret badger | 2008 | 2 | Unpublished |
| EU086185 | China | 02050CHI | Human | 1992 | 4 |  |
| EU159388 | China | CQ92 | Dog | 1992 | 1,2,3,4 | Unpublished |
| FJ561726 | China | FJ001 | Dog | 2008 | 4 | Unpublished |
| FJ561727 | China | FJ002 | Dog | 2008 | 4 | Unpublished |
| FJ561728 | China | FJ003 | Dog | 2008 | 4 | Unpublished |
| FJ561729 | China | FJ004 | Dog | 2008 | 4 | Unpublished |
| FJ561730 | China | FJ005 | Dog | 2008 | 1,2,3,4 | Unpublished |
| FJ561731 | China | FJ006 | Dog | 2008 | 1,2,3,4 | Unpublished |
| FJ561732 | China | FJ007 | Dog | 2008 | 4 | Unpublished |
| FJ866827 | China | FJ010 | Dog | 2008 | 4 | Unpublished |
| FJ866828 | China | FJ011 | Dog | 2008 | 4 | Unpublished |
| FJ866829 | China | FJ012 | Dog | 2008 | 1,2,3,4 | Unpublished |
| FJ866835 | China | FJ008 | Dog | 2007 | 4 | Unpublished |
| FJ866836 | China | FJ009 | Dog | 2008 | 4 | Unpublished |
| EU828653 | China | GN07 | Dog | 2007 | 1,2,3,4 | Unpublished |
| DQ666287 | China | Guangxi_Yl66 | Dog | 2004 | 4 |  |
| DQ866080 | China | GXB03 | Dog | 2003 | 3 |  |
| DQ866081 | China | GXcat | Cat | 2005 | 3 |  |
| DQ866082 | China | GXGG | Dog | 2005 | 3 |  |
| DQ866083 | China | GXGL | Cattle | 2004 | 3 |  |
| DQ866084 | China | GXNniu | Cattle | 2005 | 3 |  |
| DQ866085 | China | GXPX | Dog | 2005 | 3 |  |
| DQ866086 | China | GXQZ | Dog | 2005 | 3 |  |
| DQ866087 | China | GX019 | Dog | 2005 | 3 |  |
| DQ866088 | China | GX102 | Dog | 2002 | 3 |  |
| DQ866089 | China | GX120 | Dog | 2003 | 3 |  |
| DQ866090 | China | GX123 | Dog | 2003 | 3 |  |
| DQ866092 | China | GX174 | Dog | 2003 | 3 |  |
| DQ866093 | China | GX201 | Dog | 2002 | 3 |  |
| DQ866094 | China | GX441 | Dog | 2005 | 3 |  |
| DQ866095 | China | GX442 | Dog | 2005 | 3 |  |
| DQ866097 | China | GX452 | Dog | 2005 | 3 |  |
| DQ866099 | China | GX506 | Dog | 2005 | 3 |  |
| DQ866101 | China | GX509 | Dog | 2005 | 3 |  |
| DQ866102 | China | GX510 | Dog | 2005 | 3 |  |
| DQ866103 | China | GX520 | Dog | 2005 | 3 |  |
| DQ866104 | China | GX0510 | Dog | 2005 | 3 |  |
| DQ866105 | China | GX01 | Dog | 2004 | 4 |  |
| DQ866107 | China | GX074 | Dog | 2003 | 4 |  |
| DQ866108 | China | GX08 | Dog | 2003 | 4 |  |
| DQ866110 | China | GX091 | Dog | 2004 | 4 |  |
| DQ866111 | China | GXN119 | Dog | 2000 | 1,2,3,4 |  |
| DQ866112 | China | GX195 | Dog | 2004 | 4 |  |
| DQ866114 | China | GX260 | Dog | 2004 | 4 |  |
| DQ866115 | China | GXBM | Dog | 2003 | 4 |  |
| DQ866116 | China | GXLA | Dog | 2003 | 4 |  |
| DQ866117 | China | GX304 | Dog | 2004 | 1,2,3,4 |  |
| DQ866118 | China | GXBS | Dog | 2005 | 4 |  |
| DQ866119 | China | GXHX | Dog | 2005 | 4 |  |
| DQ866120 | China | GXSL | Dog | 2005 | 1,2,3,4 |  |
| DQ866121 | China | GXWXp | Dog | 2005 | 4 |  |
| EF990578 | China | CGX0601D | Dog | 2006 | 2 |  |
| EF990579 | China | CGX0602D | Dog | 2006 | 2 |  |
| EF990584 | China | CGX0607D | Dog | 2006 | 2 |  |
| EF990585 | China | CGX0510d | Dog | 2005 | 2 |  |
| EF990587 | China | CGX0612D | Dog | 2006 | 2 |  |
| EF990599 | China | CGX0626D | Dog | 2006 | 2 |  |
| EU086175 | China | 02037CHI | Dog | 1997 | 4 |  |
| EU086182 | China | 02046CHI | Dog | 1994 | 1,2,3,4 |  |
| EU086183 | China | 02047CHI | Dog | 1994 | 4 |  |
| AB294352 | China | CHdg6 | Dog |  | 2 | Unpublished |
| GQ472472 | China | GXNN2 | Dog | 2007 | 1,2,3 | Unpublished |
| EU159385 | China | Yue1 | Dog | 1997 | 1,2,3,4 | Unpublished |
| EU159390 | China | FS | Dog | 1998 | 1,2,3,4 | Unpublished |
| EU159399 | China | Yue2 | Dog | 1994 | 4 | Unpublished |
| FJ594278 | China | N11 | Dog | 1997 | 4 | Unpublished |
| GQ472468 | China | GXHXB | Dog | 2007 | 4 | Unpublished |
| GQ472469 | China | GXLA11 | Dog | 2007 | 4 | Unpublished |
| GQ472470 | China | GXLB | Dog | 2007 | 4 | Unpublished |
| GQ472471 | China | GXLCC | Dog | 2007 | 4 | Unpublished |
| GQ472477 | China | GXQZD | Dog | 2006 | 4 | Unpublished |
| GQ472478 | China | GXYZD | Dog | 2007 | 1,2,3,4 | Unpublished |
| GU358653 | China | GX4 | Dog | 1994 | 4 | Unpublished |
| HQ118102 | China | gk5 | Dog | 2006 | 4 |  |
| DQ666288 | China | Guizhou_A10 | Human | 2004 | 1,2,3,4 |  |
| DQ666289 | China | Guizhou_A101 | Dog | 2004 | 1,2,3,4 |  |
| DQ666290 | China | Guizhou_A103 | Dog | 2004 | 4 |  |
| DQ666291 | China | Guizhou_A148 | Dog | 2004 | 4 |  |
| DQ666292 | China | Guizhou_A158 | Dog | 2004 | 4 |  |
| DQ666293 | China | Guizhou_A173 | Dog | 2004 | 4 |  |
| DQ666294 | China | Guizhou_Qx1 | Dog | 2004 | 1,2,3,4 |  |
| EF990565 | China | CGZ0502D | Dog |  | 2 |  |
| EF990566 | China | CGZ0503D | Dog | 2005 | 2 |  |
| EF990568 | China | CGZ0506D | Dog | 2005 | 2 |  |
| EF990569 | China | CGZ0509D | Dog | 2005 | 2 |  |
| EF990570 | China | CGZ0510D | Dog | 2005 | 2 |  |
| EF990574 | China | CGZ0515D | Dog | 2005 | 2 |  |
| EF990576 | China | CGZ0518H | Human | 2005 | 2 |  |
| AB294348 | China | CGZ0502D | Dog | 2005 | 2 | Unpublished |
| EU159395 | China | gg4 | Dog | 2006 | 4 | Unpublished |
| EU267777 | China | Hebei0 | Human | 2007 | 4 | Unpublished |
| EU549783 | China | BD06 | Dog | 2006 | 4 | Unpublished |
| EU828651 | China | WJ07-2 | Dog | 2007 | 4 | Unpublished |
| EU828655 | China | GC07 | Dog | 2007 | 4 | Unpublished |
| EU828657 | China | WJ07-1 | Dog | 2007 | 4 | Unpublished |
| DQ666299 | China | Henan_Sq9 | Dog | 2004 | 1,3,4 |  |
| DQ666297 | China | Henan_Hb10 | Dog | 2004 | 4 |  |
| DQ666298 | China | Henan_Sq6 | Dog | 2004 | 4 |  |
| DQ666301 | China | Henan_Sq17 | Dog | 2004 | 4 |  |
| DQ666303 | China | Henan_Sq30 | Dog | 2004 | 1,2,3,4 |  |
| DQ666304 | China | Henan_Sq35 | Dog | 2004 | 4 |  |
| DQ666306 | China | Henan_Sq59 | Dog | 2004 | 4 |  |
| EU086184 | China | 02049CHI | Sika deer | 1993 | 4 |  |
| EU159378 | China | LU | Deer | 1993 | 2 | Unpublished |
| DQ875050 | China | MRV | Mouse | 1989 | 1,2,3,4 | Unpublished |
| EF611081 | China | hubei070308 | Buffalo | 2004 | 1,2,3,4 | Unpublished |
| EU159377 | China | QC | Human | 2006 | 4 | Unpublished |
| EU159380 | China | WH5 | Dog | 2005 | 1,2,3,4 | Unpublished |
| DQ496219 | China | HuNPN01 | Human | 2006 | 4 |  |
| DQ666307 | China | Hunan_DK13 | Dog | 2004 | 4 |  |
| DQ666308 | China | Hunan_Wg12 | Dog | 2004 | 4 |  |
| DQ666310 | China | Hunan_Wg22 | Dog | 2004 | 4 |  |
| DQ666313 | China | Hunan_Wg68 | Dog | 2004 | 4 |  |
| DQ666314 | China | Hunan_Wg407 | Dog | 2004 | 1,2,3,4 |  |
| DQ666317 | China | Hunan_Xx33 | Dog | 2004 | 1,2,3,4 |  |
| EU643590 | China | HN10_Hunan | Dog | 2006 | 4 |  |
| EF990600 | China | CHN0601D | Dog | 2006 | 2 |  |
| EF990601 | China | CHN0602D | Dog | 2006 | 2 |  |
| EF990602 | China | CHN0503D | Dog | 2005 | 2 |  |
| EF990604 | China | CHN0505D | Dog | 2005 | 2 |  |
| EF990611 | China | CHN0517D | Dog | 2005 | 2 |  |
| EF990618 | China | CHN0532D | Dog | 2005 | 2 |  |
| EF990620 | China | CHN0610H | Human | 2006 | 2 |  |
| EF990621 | China | CHN0635D | Human | 2006 | 2 |  |
| EU086186 | China | 05005CHI | Dog | 2005 | 4 |  |
| DQ515993 | China | HuNDN16 | Dog | 2006 | 4 | Unpublished |
| EU008919 | China | HNDB11 | Dog | 2005 | 1,2,3,4 | Unpublished |
| EU008920 | China | HNDB12 | Dog | 2006 | 1,2,3,4 | Unpublished |
| EU008922 | China | HNDB28 | Dog | 2006 | 4 | Unpublished |
| EU008923 | China | HNDB33 | Dog | 2006 | 4 | Unpublished |
| EU652444 | China | NeiMeng927B | Raccoon Dog | 2007 | 4 |  |
| EU284093 | China | NeiMeng927A | Raccoon Dog | 2007 | 1,2,3,4 |  |
| FJ415313 | China | NeiMeng925 | Raccoon Dog | 2008 | 1,3,4 |  |
| DQ666320 | China | Jiangsu_Wx0 | Human | 2004 | 4 |  |
| DQ666321 | China | Jiangsu_Wx1 | Dog | 2004 | 4 |  |
| DQ666322 | China | Jiangsu_Yc63 | Dog | 2004 | 1,2,3,4 |  |
| EU086173 | China | 9811CHI | Dog | 1998 | 4 |  |
| EU159382 | China | JSL27 | Dog | 2005 | 1,3,4 | Unpublished |
| EU159383 | China | JSL29 | Dog | 2005 | 1,3,4 | Unpublished |
| EU159384 | China | JSS62 | Dog | 2005 | 1,3,4 | Unpublished |
| GU233765 | China | JX09-17 | Ferret badger | 2009 | 1,2,3,4 |  |
| EU086188 | China | 05007CHI | Dog | 2004 | 1,3,4 |  |
| EU159389 | China | NC | Dog | 2004 | 4 | Unpublished |
| FJ719751 | China | JX08-47 | Ferret badger | 2008 | 1,2,3,4 | Unpublished |
| FJ719755 | China | JX08-58 | Ferret badger | 2008 | 4 | Unpublished |
| GU647092 | China | JX08-45 | Ferret badger | 2008 | 4 | Unpublished |
| DQ875051 | China | DRV | deer | 1989 | 1,2,3,4 | Unpublished |
| EU159387 | China | J | Human | 1985 | 4 | Unpublished |
| EU159398 | China | nu | Human | 1986 | 4 | Unpublished |
| GU591790 | China | Shaanxi-HZ-6 | Dog | 2009 | 4 | Unpublished |
| EU086176 | China | 02040CHI | Human | 1992 | 1,2,3,4 |  |
| EU086189 | China | 05008CHI | Dog | 2004 | 4 |  |
| EU086190 | China | 05009CHI | Dog | 2005 | 4 |  |
| EU159394 | China | SH06 | Dog | 2006 | 1,2,3 | Unpublished |
| EU159392 | China | SBH | Human | 1992 | 4 | Unpublished |
| EU159393 | China | SBD | Dog | 1992 | 4 | Unpublished |
| GU345748 | China | SH06 | Dog | 2006 | 4 | Unpublished |
| GU591792 | China | Sichuan-BZ-1 | Dog | 2009 | 4 | Unpublished |
| EU095330 | China | Yunnan_Md06 | Dog | 2006 | 4 | Unpublished |
| EU275243 | China | Yunnan_Tc06 | Dog | 2006 | 1,2,3,4 | Unpublished |
| EU275244 | China | Yunnan_Zt07 | Dog | 2007 | 4 | Unpublished |
| EU275245 | China | Yunnan_Qj07 | Dog | 2007 | 4 | Unpublished |
| EU282381 | China | CHVC06 | Mouse | 2006 | 1,2,3,4 | Unpublished |
| EF556197 | China | Zhejiang Wz0 | Human | 2008 | 1,2,3,4 |  |
| HQ118103 | China | ZJCA1 | Dog | 2008 | 4 |  |
| HQ118105 | China | ZJD1 | Dog | 2008 | 4 |  |
| HQ118107 | China | ZJD3 | Dog | 2008 | 4 |  |
| HQ118113 | China | ZJD11 | Dog | 2008 | 4 |  |
| HQ118114 | China | ZJF1 | Ferret badger | 2008 | 4 |  |
| EU700032 | China | Zhejiang Wz1(H) | Human | 2008 | 4 |  |
| FJ032315 | China | D03 | Dog | 2008 | 2 | Unpublished |
| EU159397 | China | LH | Dog | 2006 | 1,2,3,4 | Unpublished |
| FJ598135 | China | ZJ-LA | Ferret badger | 2008 | 4 | Unpublished |
| FJ712195 | China | F02 | Ferret badger | 2008 | 4 | Unpublished |
| FJ712196 | China | F04 | Ferret badger | 2008 | 4 | Unpublished |
| EF611857 | India | I_145 |  |  | 2,3 |  |
| EF611858 | India | I_141 |  |  | 2,3 |  |
| EF611859 | India | I_129 |  |  | 2,3 |  |
| EF611860 | India | I_123 |  |  | 3 |  |
| EF611861 | India | I_117 |  |  | 3 |  |
| EF611862 | India | I_116 |  |  | 3 |  |
| AF374721 | India |  | Human | 1997 | 1,2,3 |  |
| AY352493 | India | RV61 | Human |  | 2,3 |  |
| DQ105943 | India | R76 | Dog |  | 3 |  |
| DQ105952 | India | R116 | Dog |  | 3 |  |
| DQ105955 | India | R122 | Cow |  | 3 |  |
| DQ105957 | India | R134 | Dog |  | 3 |  |
| DQ105958 | India | R135 | Cow |  | 3 |  |
| DQ105959 | India | R141 | Dog |  | 3 |  |
| DQ105960 | India | R142 | Dog |  | 3 |  |
| DQ105962 | India | R144 | Dog |  | 3 |  |
| DQ105963 | India | R145 | Dog |  | 3 |  |
| DQ105964 | India | R155 | Cow |  | 3 |  |
| EU086191 | India | 9702INDI | Human | 1997 | 1,2,3 |  |
| AM689533 | India | BNG4 | Dog | 2006 | 3 | Unpublished |
| AM689535 | India | AB21 | Human | 2006 | 3 | Unpublished |
| AM689536 | India | AB22 | Cattle | 2006 | 3 | Unpublished |
| AY956319 | India |  | Human |  | 1,2 | Unpublished |
| DQ286762 | India | CVS | Dog |  | 2,3 | Unpublished |
| DQ991108 | India | MVCDB5 | Dog | 2005 | 3 | Unpublished |
| DQ991110 | India | MVCDB7 | Dog | 2005 | 3 | Unpublished |
| DQ991112 | India | MVCDB9 | Dog | 2006 | 3 | Unpublished |
| DQ991113 | India | MVCDB10 | Dog | 2006 | 3 | Unpublished |
| DQ991115 | India | MVCGB13 | Goat | 2006 | 3 | Unpublished |
| DQ991125 | India | NIMMB9 | Dog | 2006 | 3 | Unpublished |
| DQ991126 | India | NIMMB10 | Cattle | 2006 | 3 | Unpublished |
| EF437215 | India | NNV-RAB-H | Human | 2007 | 1,2,3 | Unpublished |
| GU731659 | India | SRV Cat TN1 | Cat | 2006 | 3 | Unpublished |
| GU731661 | India | SRV Goat TN1 | Goat | 2006 | 3 | Unpublished |
| GU731663 | India | SRV Human TN1 | Human | 2006 | 3 | Unpublished |
| GU731666 | India | SRV Dog KA2 | Dog | 2007 | 3 | Unpublished |
| GU731667 | India | SRV Dog TN1 | Dog | 2007 | 3 | Unpublished |
| HQ829841 | India | ptzn | Dog | 2009 | 1,2,3 | Unpublished |
| HQ829842 | India | SPY-BN |  | 2009 | 2 | Unpublished |
| AB154208 | Indonesia | SC01-68 | Cat | 2001 | 2,3 |  |
| AB154209 | Indonesia | SC01-74 | Cat | 2002 | 3 |  |
| AB154210 | Indonesia | SC01-75 | Cat | 2001 | 3 |  |
| AB154211 | Indonesia | SC02-82 | Cat | 2002 | 3 |  |
| AB154212 | Indonesia | SC01-73 | Cattle | 2001 | 3 |  |
| AB154213 | Indonesia | SC01-66 | Civet cat | 2001 | 2,3 |  |
| AB154214 | Indonesia | SC01-65 | Deer | 2001 | 3 |  |
| AB154215 | Indonesia | FL01-06 | Dog | 2001 | 2,3 |  |
| AB154216 | Indonesia | FL01-08 | Dog | 2002 | 3 |  |
| AB154220 | Indonesia | JA97-05 | Dog | 1997 | 3 |  |
| AB154221 | Indonesia | KL00-18 | Dog | 2000 | 3 |  |
| AB154222 | Indonesia | FL01-27 | Dog | 2001 | 3 |  |
| AB154223 | Indonesia | KL97-03 | Dog | 1997 | 3 |  |
| AB154224 | Indonesia | SC02-87 | Monkey | 2002 | 3 |  |
| AB154225 | Indonesia | SC00-12 | Dog | 2000 | 2,3 |  |
| AB154226 | Indonesia | SC00-36 | Dog | 2000 | 2,3 |  |
| AB154227 | Indonesia | SC00-45 | Dog | 2000 | 2,3 |  |
| AB154228 | Indonesia | SC01-63 | Dog | 2001 | 3 |  |
| AB154229 | Indonesia | SC02-79 | Dog | 2002 | 3 |  |
| AB154230 | Indonesia | SC02-83 | Dog | 2002 | 3 |  |
| AB154231 | Indonesia | SC02-89 | Dog | 2002 | 3 |  |
| AB154232 | Indonesia | SC02-91 | Dog | 2002 | 3 |  |
| AB154233 | Indonesia | SC97-01 | Dog | 1997 | 2,3 |  |
| AB154234 | Indonesia | SN00-03 | Dog | 2000 | 2,3 |  |
| AB154235 | Indonesia | SN00-14 | Dog | 2000 | 2,3 |  |
| AB154237 | Indonesia | SS01-13 | Dog | 2001 | 3 |  |
| AB154238 | Indonesia | SS01-21 | Dog | 2001 | 3 |  |
| AB154239 | Indonesia | SW01-11 | Dog | 2001 | 3 |  |
| AB154240 | Indonesia | SW02-22 | Dog | 2002 | 2,3 |  |
| AB154241 | Indonesia | SW97-04 | Dog | 1997 | 2,3 |  |
| AB154242 | Indonesia | SC01-70 | Tiger | 2001 | 2,3 |  |
| AB154243 | Indonesia | SC02-90 | Dog | 2002 | 2,3 |  |
| EU086192 | Indonesia | 03003INDO | Dog | 2003 | 1,2,3 |  |
| AY352494 | Japan |  | Dog | 1940s | 2,3 |  |
| AB178890 | Japan | Komatsugawa |  | 1940s | 1,2,3 |  |
| AB178891 | Japan | Takamen | Human | 1940s | 1,2,3 |  |
| AY352490 | Kazakhstan | 341f | Red fox |  | 2,3 |  |
| AY352491 | Kazakhstan | 408s | Sheep | 2004 | 2,3 |  |
| AY352492 | Kazakhstan | 409f | Red fox |  | 2,3 |  |
| AY352489 | Kazakhstan | RV259 | Red fox | 2004 | 2,3 |  |
| EU086193 | Laos | 9910LAO | Dog | 1999 | 1,2,3 |  |
| EU086194 | Laos | 02001LAO | Dog | 2002 | 1,2,3 |  |
| AB571002 | Mongolia | MGL-20 | Cattle | 2006 | 2 |  |
| AB571005 | Mongolia | MGL-23 | Camel | 2008 | 2 |  |
| AB571017 | Mongolia | MGL-35 | Sheep | 2005 | 2 |  |
| AB571018 | Mongolia | MGL_36 | Dog | 2005 | 1 |  |
| AB570996 | Mongolia | MGL-5 | Red fox | 2006 | 2,3 |  |
| AB570997 | Mongolia | MGL-10 | Cattle | 2006 | 1,2,3 |  |
| AB570999 | Mongolia | MGL-12 | Camel | 2006 | 2,3 |  |
| AB571004 | Mongolia | MGL-22 | Dog | 2007 | 1,2,3 |  |
| AB571007 | Mongolia | MGL-25 | Dog | 2008 | 1,2,3 |  |
| AB571008 | Mongolia | MGL-26 | Cattle | 2006 | 2,3 |  |
| AB571011 | Mongolia | MGL-29 | Goat | 2008 | 3 |  |
| EF614256 | Mongolia | Mongolia 4 | Wolf | 2006 | 3 | Unpublished |
| EF614257 | Mongolia | Mongolia 3 | Cow | 2006 | 2 | Unpublished |
| EU086164 | Myanmar | 9909BIR | Dog | 1999 | 1,2,3 |  |
| EU086165 | Myanmar | 9913BIR | Dog | 1999 | 1,2,3 |  |
| EU086166 | Myanmar | 9915BIR | Dog | 1999 | 1,2,3 |  |
| U22918 | Nepal | 94260NEP |  |  | 3 |  |
| EU086196 | Nepal | 9901NEP | Dog | 1998 | 1,2,3 |  |
| EU086197 | Nepal | 9902NEP | Goat | 1998 | 2,3 |  |
| EU086198 | Nepal | 9903NEP | Mongoose | 1998 | 2 |  |
| AY102996 | Pakistan | Rv193 | Dog | 1989 | 3 |  |
| AY352495 | Pakistan | 196p | Cow | 1990 | 1,2,3 |  |
| AY352496 | Pakistan | 277p | Goat | 1990 | 1,2 |  |
| AY062069 | Pakistan | Rv277 | Goat |  | 3 |  |
| AY103001 | Philippines | RV1120 | Human |  | 3 |  |
| AY103002 | Philippines | RV1121 | Dog |  | 3 |  |
| AY103003 | Philippines | 94270phi | Dog |  | 3 |  |
| AY103004 | Philippines | 94273phi | Dog |  | 3 |  |
| AY103005 | Philippines | 94280phi | Dog |  | 3 |  |
| AB070759 | Philippines | Phi123-01 |  |  | 3 |  |
| AB070760 | Philippines | Phi124-02 |  |  | 3 |  |
| AB070761 | Philippines | Phi127-03 |  |  | 3 |  |
| AB070763 | Philippines | Phi131-05 |  |  | 3 |  |
| AB070764 | Philippines | Phi134-06 |  |  | 3 |  |
| AB070765 | Philippines | Phi135-07 |  |  | 3 |  |
| AB070772 | Philippines | Phi152-14 |  |  | 3 |  |
| AB070776 | Philippines | Phi171-18 |  |  | 3 |  |
| AB070778 | Philippines | Phi179-21 |  |  | 3 |  |
| AB070779 | Philippines | Phi184-21 |  |  | 3 |  |
| AB070780 | Philippines | Phi191-22 |  |  | 3 |  |
| AB070781 | Philippines | Mdn034/24 |  |  | 3 |  |
| AB070789 | Philippines | Mdn091/35 |  |  | 3 |  |
| AB070795 | Philippines | Mdn112/41 |  |  | 3 |  |
| AB070798 | Philippines | Mdn127/48 |  |  | 3 |  |
| AB070802 | Philippines | Mdn133/54 |  |  | 3 |  |
| AB070803 | Philippines | Mdn134/55 |  |  | 3 |  |
| AB070805 | Philippines | Mdn137/58 |  |  | 3 |  |
| AB070806 | Philippines | Mdn138/59 |  |  | 3 |  |
| AB070807 | Philippines | Mdn139/60 |  |  | 3 |  |
| AB070811 | Philippines | Mdn144/65 |  |  | 3 |  |
| AB070816 | Philippines | Mdn150/71 |  |  | 3 |  |
| AB070817 | Philippines | Mdn183/45 |  |  | 3 |  |
| AB573762 | Philippines | Kyoto | Human | 2006 | 2,3 |  |
| AB573763 | Philippines | Yokohama | Human | 2006 | 2,3 |  |
| EU086200 | Philippines | 94270PHI | Dog | 1994 | 2,3 |  |
| EU086201 | Philippines | 94273PHI | Dog | 1994 | 1,2,3 |  |
| EU086202 | Philippines | 94280PHI | Dog | 1994 | 1,2,3 |  |
| EU086203 | Philippines | 03006PHI | Human | 2000 | 1,2,3 |  |
| EU086204 | Philippines | 03007PHI | Human | 2001 | 2,3 |  |
| EU086205 | Philippines | 04030PHI | Human | 2004 | 1,2 |  |
| EF611836 | Russia | SG16 | Arctic fox | 1950-1960 | 2 |  |
| EF611828 | Russia | SG21 | Arctic fox | 1987 | 3 |  |
| EF611831 | Russia | SG22 | Arctic fox | 1987 | 3 |  |
| EF611835 | Russia | SG92 | Arctic fox | 1950-1960 | 3 |  |
| EF611838 | Russia | SG10 | Arctic fox | 1950-1960 | 1,3 |  |
| EF611840 | Russia | SG15 | Arctic fox | 1950-1960 | 3 |  |
| EF611868 | Russia | 994_dog | Dog | 1980 | 2,3 |  |
| U22656 | Russia | 9141RUS | Arctic fox | 1988 | 1,2,3 |  |
| DQ010125 | Russia | RV255 | Arctic fox | 1988 | 3 |  |
| DQ010127 | Russia | RV443 | Horse | 1990 | 3 |  |
| DQ010128 | Russia | RV1334 | Wolf | 2002 | 3 |  |
| DQ010129 | Russia | RV1336 | Arctic fox | 2002 | 3 |  |
| DQ010131 | Russia | RV1338 | Arctic fox | 2002 | 3 |  |
| AY352457 | Russia | RV262 | Red fox |  | 2 |  |
| AY352481 | Russia | 3561d | Dog |  | 2 |  |
| AY352455 | Russia | 3502f | Red fox |  | 3 |  |
| AY352456 | Russia | RV1589 | Cat |  | 2,3 |  |
| AY352458 | Russia | 857r | Raccoon dog | 1980 | 1,2,3 |  |
| AY352459 | Russia | 304c | Steppe fox | 1977 | 1,2,3 |  |
| AY352461 | Russia | 1305f | Red fox |  | 2,3 |  |
| AY352462 | Russia | RVHK | Cat | 1998 | 2,3 |  |
| AY352463 | Russia | RVHN | Human |  | 2,3 |  |
| AY352464 | Russia | RV257 | Red fox |  | 2,3 |  |
| AY352465 | Russia | RV260 | Red fox |  | 2,3 |  |
| AY352466 | Russia | 3665f | Red fox |  | 3 |  |
| AY352467 | Russia | 3605f | Red fox |  | 2,3 |  |
| AY352468 | Russia | 3678c | steppe fox |  | 3 |  |
| AY352469 | Russia | 3683c | steppe fox |  | 3 |  |
| AY352472 | Russia | RV1590 | Human |  | 2,3 |  |
| AY352473 | Russia | 999c | Cat |  | 3 |  |
| AY352475 | Russia | RV245 | Human |  | 3 |  |
| AY352476 | Russia | RV234 | Dog |  | 3 |  |
| AY352477 | Russia | RV241 | Human |  | 2,3 |  |
| AY352478 | Russia | RV298 | Cow |  | 3 |  |
| AY352480 | Russia | RV250 | Squirrel |  | 2,3 |  |
| AY352482 | Russia | 686cow | Cow |  | 1,2,3 |  |
| AY352483 | Russia | 765w | Wolf |  | 1,2,3 |  |
| AY352484 | Russia | 2070f | Red fox |  | 3 |  |
| AY352485 | Russia | 2072f | Red fox |  | 3 |  |
| AY352486 | Russia | 3510w | Wolf |  | 2,3 |  |
| AY352502 | Russia | RV304 |  |  | 3 |  |
| AY352503 | Russia | RV290 |  |  | 3 |  |
| AY352507 | Russia | RV237 |  |  | 3 |  |
| AY352509 | Russia | RV239 |  |  | 3 |  |
| AY352510 | Russia | RV246 |  |  | 3 |  |
| AY352513 | Russia | RV293 |  |  | 3 |  |
| AY352514 | Russia | RV294 |  |  | 3 |  |
| AY062071 | Russia | Rv450 | Fox |  | 3 |  |
| DQ317516 | Russia | RV240 | Human | 1988 | 3 | Unpublished |
| DQ317520 | Russia | RV448 | Red fox | 1990 | 3 | Unpublished |
| AY730595 | South Korea | KRH2-04 | Raccoon dog | 2004 | 2,3 |  |
| AY730596 | South Korea | KRH3-04 | Dog | 2004 | 1,2 |  |
| GU937039 | South Korea | KRVR0801 | Raccoon dog | 2008 | 1,2,3 |  |
| GU937044 | South Korea | KRVB0903 | Bovine | 2009 | 2,3 |  |
| DQ076121 | South Korea | SKRRD9902PJ | Raccoon dog | 1999 | 2,3 |  |
| DQ076124 | South Korea | SKRDG0203CW | Dog | 2002 | 1,2 |  |
| DQ076127 | South Korea | SKRRD0205HC | Raccoon dog | 2002 | 2,3 |  |
| DQ076131 | South Korea | SKRRD9903YG | Bovine | 1999 | 1,2,3 |  |
| GU937036 | South Korea | KRVR0901 | Raccoon dog | 2009 | 2 | Unpublished |
| GU937041 | Southkorea | KRVB0907 | Bovine | 2009 | 3 |  |
| GU937043 | Southkorea | KRVB0904 | Bovine | 2009 | 3 |  |
| GU937045 | Southkorea | KRVB0902 | Bovine | 2009 | 1,3 |  |
| DQ076119 | Southkorea | SKRBV9801YC | Bovine | 1998 | 3 |  |
| DQ076123 | Southkorea | SKRRD9901PJ | Raccoon dog | 1999 | 2,3 |  |
| DQ076129 | Southkorea | SKRBV0403CW | Bovine | 2004 | 3 |  |
| AY102995 | Sri Lanka | Rv69 | Dog |  | 3 |  |
| AB041964 | Sri Lanka | SRL1032 | Jackal | 1996 | 2 |  |
| AB041965 | Sri Lanka | SRL1036 | Human | 1996 | 2 |  |
| AB041966 | Sri Lanka | SRL1060 | Dog | 1996 | 1,3 |  |
| AB041967 | Sri Lanka | SRL1077 | Mongoose | 1996 | 1,3 |  |
| AB041968 | Sri Lanka | SRL1143 | Cat | 1996 | 3 |  |
| AB041969 | Sri Lanka | SRL1145 | Buffalo | 1996 | 2,3 |  |
| U22917 | Sri Lanka | 94257SRI |  |  | 3 |  |
| AB638768 | Sri Lanka | H-1218-08 | Human | 2008 | 2,3 |  |
| AB638769 | Sri Lanka | H-1281-08 | Human | 2008 | 2 |  |
| AB638772 | Sri Lanka | H-1366-09 | Cat | 2009 | 2,3 |  |
| AY138549 | Sri Lanka | 1294 | Dog | 1986 | 1,2 |  |
| AB569299 | Sri Lanka | H-08-1320 | Human | 2008 | 1,2,3 |  |
| DQ267925 | Thailand | D664-25 | Dog |  | 3 |  |
| GQ303555 | Thailand | UA341 | Human | 2009 | 1,3 |  |
| GQ303556 | Thailand | D9/52 | Dog | 2009 | 3 |  |
| U22653 | Thailand | 8738THA |  |  | 3 |  |
| EU293111 | Thailand | 8764THA | Human | 1983 | 3 |  |
| EU293121 | Thailand | 8743THA |  |  | 3 |  |
| AB178892 | Thailand | THA1013 | Dog | 1985 | 1 |  |
| AB178894 | Thailand | THA1017 | Dog | 1985 | 1 |  |
| AB178895 | Thailand | THA_Abha | Dog | 1977 | 1 |  |
| AB178896 | Thailand | THA_AY | Dog | 1963 | 1 |  |
| AY218996 | Thailand | D23 | Dog |  | 2,3 |  |
| AY218997 | Thailand | D48 | Dog |  | 3 |  |
| AY218998 | Thailand | D51 | Dog |  | 3 |  |
| AY218999 | Thailand | HM208 | Human |  | 3 |  |
| AY219000 | Thailand | HM65 | Human |  | 3 |  |
| AY219001 | Thailand | HM75 | Human |  | 3 |  |
| AY219002 | Thailand | HM88 | Human |  | 3 |  |
| EU086206 | Thailand | 8734THA | Human | 1983 | 3 |  |
| EU086207 | Thailand | 8743THA | Human | 1983 | 1,3 |  |
| EU086208 | Thailand | 8738THA | Human | 1983 | 1,2,3 |  |
| EU086216 | Thailand | 8664THA | Dog | 1986 | 3 |  |
| EU086217 | Thailand | 8758THA | Human | 1983 | 3 |  |
| EU086218 | Thailand | 8760THA | Human | 1983 | 3 |  |
| AY849023 | Thailand | 26NPpmt | Dog | 2001 | 2 |  |
| AY849024 | Thailand | 53SPppd | Dog | 1999 | 2 |  |
| AY849035 | Thailand | 217SSm | Dog | 1999 | 2 |  |
| AY849061 | Thailand | 333KJm | Dog | 2001 | 2 |  |
| AY849064 | Thailand | 38/43 | Dog | 2000 | 2 |  |
| AY849099 | Thailand | 408SKhy | Dog | 2002 | 2 |  |
| AY849113 | Thailand | 156PTns | Dog | 1999 | 2 |  |
| AY849121 | Thailand | 157PJsry | Dog | 1999 | 2 |  |
| AY849192 | Thailand | HMS241CL | Human | 2001 | 2 |  |
| AY849212 | Thailand | 685BRppc | Dog | 2002 | 2 |  |
| AY849227 | Thailand | HMS223RY | Human | 2002 | 2 |  |
| AY849241 | Thailand | 807UDts | Dog | 2002 | 2 |  |
| AY849253 | Thailand | 785SUkkl | Dog | 2003 | 2 |  |
| GU992307 | Thailand | 8764THA | Dog | 1983 | 3 | Unpublished |
| EU086209 | Vietnam | 01016VNM | Dog | 2001 | 1,2,3 |  |
| EU086210 | Vietnam | 01017VNM | Dog | 2001 | 1,2 |  |
| AB116579 | Vietnam | VN3 | Dog |  | 3 | Unpublished |
| AB116580 | Vietnam | VN52 | Dog |  | 3 | Unpublished |
| AB299032 | Vietnam | HCM1 | Dog |  | 3 | Unpublished |
| AB299034 | Vietnam | HCM5 | Dog |  | 3 | Unpublished |
| AB299035 | Vietnam | HCM6 | Dog |  | 3 | Unpublished |
| AB299037 | Vietnam | HCM8 | Dog |  | 3 | Unpublished |
| AB299038 | Vietnam | HCM9 | Dog |  | 3 | Unpublished |

References

1. Hoffmann B, Freuling CM, Wakeley PR, Rasmussen TB, Leech S, et al. (2010) Improved safety for molecular diagnosis of classical rabies viruses by use of a TaqMan real-time reverse transcription-PCR "double check" strategy. J Clin Microbiol 48: 3970-3978.

2. Bourhy H, Reynes JM, Dunham EJ, Dacheux L, Larrous F, et al. (2008) The origin and phylogeography of dog rabies virus. J Gen Virol 89: 2673-2681.

3. Tenzin, Wacharapluesadee S, Denduangboripant J, Dhand NK, Dorji R, et al. (2011) Rabies virus strains circulating in Bhutan: implications for control. Epidemiol Infect 139: 1457-1462.

4. Meng S, Sun Y, Wu X, Tang J, Xu G, et al. (2011) Evolutionary dynamics of rabies viruses highlights the importance of China rabies transmission in Asia. Virology 410: 403-409.

5. Zhang YZ, Xiong CL, Lin XD, Zhou DJ, Jiang RJ, et al. (2009) Genetic diversity of Chinese rabies viruses: evidence for the presence of two distinct clades in China. Infect Genet Evol 9: 87-96.

6. Zhu Y, Zhang G, Shao M, Lei Y, Jiang Y, et al. (2011) An outbreak of sheep rabies in Shanxi province, China. Epidemiol Infect: 1-4.

7. Smith J, McElhinney L, Parsons G, Brink N, Doherty T, et al. (2003) Case report: rapid ante-mortem diagnosis of a Human case of rabies imported into the UK from the Philippines. J Med Virol 69: 150-155.

8. Zhang YZ, Xiong CL, Zou Y, Wang DM, Jiang RJ, et al. (2006) Molecular characterization of rabies virus isolates in China during 2004. Virus Res 121: 179-188.

9. Liu Q, Xiong Y, Luo TR, Wei YC, Nan SJ, et al. (2007) Molecular epidemiology of rabies in Guangxi Province, south of China. J Clin Virol 39: 295-303.

10. Tao XY, Tang Q, Li H, Mo ZJ, Zhang H, et al. (2009) Molecular epidemiology of rabies in Southern People's Republic of China. Emerg Infect Dis 15: 1192-1198.

11. Jiang Y, Yu X, Wang L, Lu Z, Liu H, et al. (2008) An outbreak of pig rabies in Hunan province, China. Epidemiol Infect 136: 504-508.

12. Ming P, Du J, Tang Q, Yan J, Nadin-Davis SA, et al. (2009) Molecular characterization of the complete genome of a street rabies virus isolated in China. Virus Res 143: 6-14.

13. Shao XQ, Yan XJ, Luo GL, Zhang HL, Chai XL, et al. (2011) Genetic evidence for domestic raccoon dog rabies caused by Arctic-like rabies virus in Inner Mongolia, China. Epidemiol Infect 139: 629-635.

14. Zhang S, Zhao J, Liu Y, Fooks AR, Zhang F, et al. (2010) Characterization of a rabies virus isolate from a ferret badger (Melogale moschata) with unique molecular differences in glycoprotein antigenic site III. Virus Res 149: 143-151.

15. Yao WR, Pan,G.Q., Xiong,C.L., Zhou,Q.F., Xiao,Q.Y., Li,M.H. and Zhang,Y.Z (2007) Detection and Genetic Characterization of Rabies Virus from Human Patients. Virol Sin 22 (4), 307-315.

16. Kuzmin IV, Hughes GJ, Botvinkin AD, Gribencha SG, Rupprecht CE (2008) Arctic and Arctic-like rabies viruses: distribution, phylogeny and evolutionary history. Epidemiol Infect 136: 509-519.

17. Jayakumar R, Tirumurugaan KG, Ganga G, Kumanan K, Mahalinga Nainar A (2004) Characterization of nucleoprotein gene sequence of an Indian isolate of rabies virus. Acta Virol 48: 47-50.

18. Kuzmin IV, Botvinkin AD, McElhinney LM, Smith JS, Orciari LA, et al. (2004) Molecular epidemiology of terrestrial rabies in the former Soviet Union. J Wildl Dis 40: 617-631.

19. Nagarajan T, Nagendrakumar SB, Mohanasubramanian B, Rajalakshmi S, Hanumantha NR, et al. (2009) Phylogenetic analysis of nucleoprotein gene of dog rabies virus isolates from Southern India. Infect Genet Evol 9: 976-982.

20. Susetya H, Sugiyama M, Inagaki, Ito N, Mudiarto G, et al. (2008) Molecular epidemiology of rabies in Indonesia. Virus Res 135: 144-149.

21. Arai YT (2004) Phylogenetic analysis of two rabies viruses, Takamen and Komatsugawa strains isolated in Japan in the 1940's. Kansenshogaku Zasshi 78: 815-822.

22. Boldbaatar B, Inoue S, Tuya N, Dulam P, Batchuluun D, et al. (2010) Molecular epidemiology of rabies virus in Mongolia, 2005-2008. Jpn J Infect Dis 63: 358-363.

23. Kissi B, Tordo N, Bourhy H (1995) Genetic polymorphism in the rabies virus nucleoprotein gene. Virology 209: 526-537.

24. Johnson N, McElhinney LM, Smith J, Lowings P, Fooks AR (2002) Phylogenetic comparison of the genus Lyssavirus using distal coding sequences of the glycoprotein and nucleoprotein genes. Archives of Virology 147: 2111-2123.

25. Nishizono A, Mannen K, Elio-Villa LP, Tanaka S, Li KS, et al. (2002) Genetic analysis of rabies virus isolates in the Philippines. Microbiol Immunol 46: 413-417.

26. Mansfield KL, Racloz V, McElhinney LM, Marston DA, Johnson N, et al. (2006) Molecular epidemiological study of Arctic rabies virus isolates from Greenland and comparison with isolates from throughout the Arctic and Baltic regions. Virus Res 116: 1-10.

27. Park YJ, Shin MK, Kwon HM (2005) Genetic characterization of rabies virus isolates in Korea. Virus Genes 30: 341-347.

28. Yang DK, Shin EK, Oh YI, Kang HK, Lee KW, et al. (2011) Molecular epidemiology of rabies virus circulating in South Korea, 1998-2010. J Vet Med Sci 73: 1077-1082.

29. Hyun BH, Lee KK, Kim IJ, Lee KW, Park HJ, et al. (2005) Molecular epidemiology of rabies virus isolates from South Korea. Virus Res 114: 113-125.

30. Arai YT, Takahashi H, Kameoka Y, Shiino T, Wimalaratne O, et al. (2001) Characterization of Sri Lanka rabies virus isolates using nucleotide sequence analysis of nucleoprotein gene. Acta Virol 45: 327-333.

31. Matsumoto T, Ahmed K, Wimalaratne O, Nanayakkara S, Perera D, et al. (2011) Novel sylvatic rabies virus variant in endangeRed golden palm civet, Sri Lanka. Emerg Infect Dis 17: 2346-2349.

32. Nanayakkara S, Smith JS, Rupprecht CE (2003) Rabies in Sri Lanka: splendid isolation. Emerg Infect Dis 9: 368-371.

33. Matsumoto T, Ahmed K, Wimalaratne O, Yamada K, Nanayakkara S, et al. (2011) Whole-genome analysis of a Human rabies virus from Sri Lanka. Arch Virol 156: 659-669.

34. Wacharapluesadee S, Ruangvejvorachai P, Hemachudha T (2006) A simple method for detection of rabies viral sequences in 16-year old archival brain specimens with one-week fixation in formalin. J Virol Methods 134: 267-271.

35. Wilde H, Choomkasien P, Hemachudha T, Supich C, Chutivongse S (1989) Failure of rabies postexposure treatment in Thailand. Vaccine 7: 49-52.

36. Delmas O, Holmes EC, Talbi C, Larrous F, Dacheux L, et al. (2008) Genomic diversity and evolution of the lyssaviruses. PLoS One 3: e2057.

37. Hemachudha T, Wacharapluesadee S, Lumlertdaecha B, Orciari LA, Rupprecht CE, et al. (2003) Sequence analysis of rabies virus in Humans exhibiting encephalitic or paralytic rabies. J Infect Dis 188: 960-966.

38. Denduangboripant J, Wacharapluesadee S, Lumlertdacha B, Ruankaew N, Hoonsuwan W, et al. (2005) Transmission dynamics of rabies virus in Thailand: implications for disease control. BMC Infect Dis 5: 52.

**Table S1.3 Dataset** 5

| **Accession** | **Location** | **Host** | **Isolate** | **Date** | **Longitude/Latitude** |
| --- | --- | --- | --- | --- | --- |
| EU086167 | Cambodia | Dog | 9908CBG | 1999 | 105.46, 11.40 |
| EU086168 | Cambodia | Dog | 9911CBG | 1998 | 104.68, 10.31 |
| EU086169 | Cambodia | Dog | 9912CBG | 1998 | 104.34, 11.42 |
| EU086170 | Cambodia | Dog | 9914CBG | 1997 | 107.02, 13.75 |
| EU086171 | Cambodia | Dog | 9916CBG | 1999 | 104.18, 10.61 |
| EU086172 | Cambodia | Dog | 02006CBG | 1998 |  |
| EU159391 | China_Anhui | Dog | H69 | 1969 | 117.20, 31.82 |
| EU159396 | China_Anhui | Dog | H | 1989 | 117.20, 31.82 |
| EU159400 | China_Anhui | Dog | H89 | 1989 | 117.20, 31.82 |
| HQ118104 | China_Anhui | Dog | HeFei | 1989 | 117.20, 31.82 |
| EU159379 | China_Anhui | Dog | FEIDONG | 1989 | 117.20, 31.82 |
| EU086185 | China_Chongqing | Human | 02050CHI | 1992 | 107.60, 29.58 |
| DQ866111 | China_Guangxi | Dog | GXN119 | 2000 | 108.58, 23.64 |
| FJ594278 | China_Guangxi | Dog | N11 | 1997 | 108.58, 23.64 |
| EF990579 | China_Guangxi | Dog | CGX0602D | 2006 | 108.58, 23.64 |
| JN974827 | China_Guangxi | Dog | CGX0606D | 2006 | 108.58, 23.64 |
| DQ666287 | China_Guangxi | Dog | Guangxi_Yl66 | 2004 | 108.58, 23.64 |
| DQ866107 | China_Guangxi | Dog | GX074 | 2003 | 108.58, 23.64 |
| DQ866118 | China_Guangxi | Dog | GXBS | 2005 | 108.58, 23.64 |
| EU159399 | China_Guangxi | Dog | Yue2 | 1994 | 108.58, 23.64 |
| GQ472478 | China_Guangxi | Dog | GXYZD | 2007 | 108.58, 23.64 |
| EU086175 | China_Guangxi | Dog | 02037CHI | 1997 | 108.58, 23.64 |
| EF990578 | China_Guangxi | Dog | CGX0601D | 2006 | 108.58, 23.64 |
| JN974828 | China_Guangxi | Dog | CGX0625D | 2006 | 108.58, 23.64 |
| DQ866105 | China_Guangxi | Dog | GX01 | 2004 | 108.58, 23.64 |
| DQ866108 | China_Guangxi | Dog | GX08 | 2003 | 108.58, 23.64 |
| DQ866114 | China_Guangxi | Dog | GX260 | 2004 | 108.58, 23.64 |
| DQ866119 | China_Guangxi | Dog | GXHX | 2005 | 108.58, 23.64 |
| DQ866121 | China_Guangxi | Dog | GXWXp | 2005 | 108.58, 23.64 |
| GU358653 | China_Guangxi | Dog | GX4 | 1994 | 108.58, 23.64 |
| GQ472468 | China_Guangxi | Dog | GXHXB | 2007 | 108.58, 23.64 |
| GQ472470 | China_Guangxi | Dog | GXLB | 2007 | 108.58, 23.64 |
| GQ472477 | China_Guangxi | Dog | GXQZD | 2006 | 108.58, 23.64 |
| EU086182 | China_Guangxi | Dog | 02046CHI | 1994 | 108.58, 23.64 |
| EU086183 | China_Guangxi | Dog | 02047CHI | 1994 | 108.58, 23.64 |
| HM486370 | China_Guangxi | Dog | CGX0516D | 2005 | 108.58, 23.64 |
| DQ866112 | China_Guangxi | Dog | GX195 | 2004 | 108.58, 23.64 |
| GQ472469 | China_Guangxi | Dog | GXLA11 | 2007 | 108.58, 23.64 |
| GQ472471 | China_Guangxi | Dog | GXLCC | 2007 | 108.58, 23.64 |
| EU159390 | China_Guangxi | Dog | FS | 1998 | 108.58, 23.64 |
| EU159385 | China_Guangxi | Dog | Yue1 | 1997 | 108.58, 23.64 |
| DQ666293 | China_Guizhou | Dog | Guizhou_A173 | 2004 | 107.22, 26.78 |
| JN974832 | China_Guizhou | Human | CGZ0924H | 2009 | 107.22, 26.78 |
| DQ666291 | China_Guizhou | Dog | Guizhou_A148 | 2004 | 107.22, 26.78 |
| DQ666294 | China_Guizhou | Dog | Guizhou_Qx1 | 2004 | 107.22, 26.78 |
| HQ118102 | China_Guizhou | Dog | gk5 | 2006 | 107.22, 26.78 |
| HM486369 | China_Guizhou | Dog | CGZ0620D | 2006 | 107.22, 26.78 |
| HM486368 | China_Guizhou | Dog | CGZ0508D | 2005 | 107.22, 26.78 |
| DQ666290 | China_Guizhou | Dog | Guizhou_A103 | 2004 | 107.22, 26.78 |
| JN974830 | China_Guizhou | Dog | CGZ0501D | 2005 | 107.22, 26.78 |
| EU159395 | China_Guizhou | Dog | gg4 | 2006 | 107.22, 26.78 |
| DQ666288 | China_Guizhou | Dog | Guizhou_A10 | 2004 | 107.22, 26.78 |
| EU159392 | China_Shanghai | Human | SBH | 1992 | 121.42, 31.16 |
| JN974836 | China_Hunan | Dog | CHN0504D | 2005 | 111.84, 27.72 |
| JN974846 | China_Hunan | Dog | CHN0809D | 2008 | 111.84, 27.72 |
| DQ666308 | China_Hunan | Dog | Hunan_Wg12 | 2004 | 111.84, 27.72 |
| HM486363 | China_Jiangsu | Dog | CJS0634D | 2006 | 119.92, 32.84 |
| EU086173 | China_Jiangsu | Dog | 9811CHI | 1998 | 119.92, 32.84 |
| JN974854 | China_Jiangxi | Dog | CJX0902D | 2009 | 115.57, 27.56 |
| JN974855 | China_Jiangxi | Dog | CJX0903D | 2009 | 115.57, 27.57 |
| DQ875051 | China_Jilin | Deer | DRV | 1989 | 125.40, 43.88 |
| JN974872 | China_Shanghai | Dog | CSH0329D | 2003 | 121.42, 31.16 |
| JN974874 | China_Shanghai | Dog | CSH0501D | 2005 | 121.42, 31.16 |
| EU086176 | China_Shanghai | Human | 02040CHI | 1992 | 121.42, 31.16 |
| EU159393 | China_Shanghai | Dog | SBD | 1992 | 121.42, 31.16 |
| GU591790 | China_Sichuan | Dog | Sichuan-BZ-1 | 2009 | 102.60, 30.63 |
| JN974859 | China_Sichuan | Dog | CSC0910D | 2009 | 102.60, 30.63 |
| EU275243 | China_Yunnan | Dog | Yunnan_Tc06 | 2006 | 101.42, 24.32 |
| JQ730682 | China_Yunnan | Dog | CYN1009D | 2010 | 101.42, 24.32 |
| JF819623 | China_Yunnan | Dog | CYN1006D | 2010 | 101.42, 24.32 |
| JF819619 | China_Yunnan | Dog | CYN1003D | 2010 | 101.42, 24.32 |
| JF819611 | China_Yunnan | Dog | CYN0919D | 2009 | 101.42, 24.32 |
| JQ040596 | China_Yunnan | Human | CYN1025H | 2010 | 101.42, 24.32 |
| EU095330 | China_Yunnan | Dog | Yunnan_Md06 | 2006 | 101.42, 24.32 |
| EU275244 | China_Yunnan | Dog | Yunnan_Zt07 | 2007 | 101.42, 24.32 |
| EU275245 | China_Yunnan | Dog | Yunnan_Qj07 | 2007 | 101.42, 24.32 |
| JF819605 | China_Yunnan | Dog | CYN0811D | 2008 | 101.42, 24.32 |
| JF819612 | China_Yunnan | Human | CYN0924H | 2009 | 101.42, 24.32 |
| JF819615 | China_Yunnan | Dog | CYN0814D | 2008 | 101.42, 24.32 |
| JF819614 | China_Yunnan | Human | CYN0921HN | 2009 | 101.42, 24.32 |
| EU282381 | China_Yunnan | Dog | CHVC06 | 2006 | 101.42, 24.32 |
| JN974878 | China_Zhejiang | Dog | CZJ0814D | 2008 | 120.05, 29.42 |
| EU159397 | China_Zhejiang | Dog | LH | 2006 | 120.05, 29.42 |
| HQ118107 | China_Zhejiang | Dog | ZJD3 | 2008 | 120.05, 29.42 |
| EU086193 | Laos_Vientiane | Dog | 9910LAO | 1999 | 102.62, 17.98 |
| EU086194 | Laos_Vientiane | Dog | 02001LAO | 2002 | 102.62, 17.98 |
| EU086195 | Laos_Vientiane | Dog | 02002LAO | 2002 | 102.62, 17.98 |
| EU086164 | Myanmar_Yangon | Dog | 9909BIR | 1999 | 96.15, 16.90 |
| EU086165 | Myanmar_Yangon | Dog | 9913BIR | 1999 | 96.15, 16.90 |
| EU086166 | Myanmar_Yangon | Dog | 9915BIR | 1999 | 96.15, 16.90 |
| EU086200 | Philippines | Dog | 94270PHI | 1994 |  |
| EU086201 | Philippines | Dog | 94273PHI | 1994 |  |
| EU086202 | Philippines | Dog | 94280PHI | 1994 |  |
| EU086203 | Philippines | Human | 03006PHI | 2000 |  |
| EU086204 | Philippines | Human | 03007PHI | 2001 |  |
| EU086205 | Philippines | Human | 04030PHI | 2004 |  |
| AY138572 | Thailand | Dog | 2908 | 1995 |  |
| AY580089 | Thailand | Dog | THA8738 |  |  |
| DQ267925 | Thailand | Dog | D664_45 |  |  |
| EU086206 | Thailand | Human | 8734THA | 1983 |  |
| EU086207 | Thailand | Human | 8743THA | 1983 |  |
| EU086208 | Thailand | Human | 8738THA | 1983 |  |
| EU293111 | Thailand | Human | 8764THA | 1983 |  |
| EU293121 | Thailand |  | 8743THA |  |  |
| GQ303555 | Thailand | Human | UA341 | 2009 |  |
| GQ303556 | Thailand | Dog | D9/52 | 2009 |  |
| GU992307 | Thailand | Dog | 8764THA | 1983 |  |
| U22653 | Thailand |  | 8738THA |  |  |
| AY849164 | Thailand_Amnat_Chareon | Dog | 486UMhsp | 2002 | 104.76, 15.90 |
| AY849165 | Thailand_Amnat_Charoen | Dog | 487UMm | 2002 | 104.76, 15.90 |
| AY849187 | Thailand_Amnat_Charoen | Dog | 488UMm | 1998 | 104.76, 15.90 |
| AY849195 | Thailand_Amnat_Charoen | Dog | 689Umm | 2001 | 104.76, 15.90 |
| AY849196 | Thailand_Amnat_Charoen | Dog | 688Umm | 2002 | 104.76, 15.90 |
| AY849123 | Thailand_Ang_Thong | Dog | 270ATvsc | 2001 | 100.30, 14.70 |
| AB178896 | Thailand_Ayudhaya | Dog | THA-AY | 1963 | 100.60, 14,02 |
| AY849128 | Thailand_Ayutthaya | Dog | 191AYsn | 1999 | 100.60, 14,02 |
| AY849136 | Thailand_Ayutthaya | Dog | 250AYbt | 1999 | 100.60, 14,02 |
| AY218997 | Thailand_Bangkok | Dog | D48 |  | 100.57, 13.77 |
| AY218998 | Thailand_Bangkok | Dog | D51 |  | 100.57, 13.78 |
| AY218999 | Thailand_Bangkok | Human | HM208 |  | 100.57, 13.79 |
| AY219000 | Thailand_Bangkok | Human | HM65 |  | 100.57, 13.80 |
| AY219002 | Thailand_Bangkok | Human | HM88 |  | 100.57, 13.81 |
| AY580090 | Thailand_Bangkok | Human | HM88BKjj | 1999 | 100.57, 13.77 |
| AY580091 | Thailand_Bangkok | Human | HM75BK | 1998 | 100.57, 13.77 |
| AY580092 | Thailand_Bangkok | Human | HM208BKpv | 2001 | 100.57, 13.77 |
| AY580112 | Thailand_Bangkok | Dog | 6BKjt | 2001 | 100.57, 13.77 |
| AY580117 | Thailand_Bangkok | Dog | 16BKwtl | 2001 | 100.57, 13.77 |
| AY580118 | Thailand_Bangkok | Dog | 19BKbkp | 2001 | 100.57, 13.77 |
| AY580119 | Thailand_Bangkok | Dog | 20Bkds | 2001 | 100.57, 13.77 |
| AY580122 | Thailand_Bangkok | Dog | 35BKbk | 2001 | 100.57, 13.77 |
| AY580123 | Thailand_Bangkok | Dog | 36BKpsc | 2001 | 100.57, 13.77 |
| AY580124 | Thailand_Bangkok | Dog | 38BKlp | 2001 | 100.57, 13.77 |
| AY580125 | Thailand_Bangkok | Dog | 40BKpp | 2001 | 100.57, 13.77 |
| AY580126 | Thailand_Bangkok | Dog | 48BKpyt | 2001 | 100.57, 13.77 |
| AY580127 | Thailand_Bangkok | Dog | 51BKds | 2001 | 100.57, 13.77 |
| AY580129 | Thailand_Bangkok | Dog | 70BKdd | 2001 | 100.57, 13.77 |
| AY580133 | Thailand_Bangkok | Human | HM65BK | 1998 | 100.57, 13.77 |
| AY580141 | Thailand_Bangkok | Cat | C267BKbkn | 2001 | 100.57, 13.77 |
| AY580142 | Thailand_Bangkok | Cat | C271BKrtv | 2001 | 100.57, 13.77 |
| AY580143 | Thailand_Bangkok | Cat | C274BKdd | 1999 | 100.57, 13.77 |
| AY580144 | Thailand_Bangkok | Cat | C276BKpv | 1999 | 100.57, 13.77 |
| AY580145 | Thailand_Bangkok | Cat | C277BKkt | 1999 | 100.57, 13.77 |
| AY849051 | Thailand_Bangkok | Cat | C267BKbkn | 2000 | 100.57, 13.77 |
| AY849053 | Thailand_Bangkok | Cat | C271BKrtv | 2001 | 100.57, 13.77 |
| AY849054 | Thailand_Bangkok | Cat | C274BKdd | 1999 | 100.57, 13.77 |
| AY849055 | Thailand_Bangkok | Cat | C276BKpv | 1999 | 100.57, 13.77 |
| AY849056 | Thailand_Bangkok | Cat | C277BKkt | 1999 | 100.57, 13.77 |
| AY849071 | Thailand_Bangkok | Dog | 51BKds | 1999 | 100.57, 13.77 |
| AY849073 | Thailand_Bangkok | Dog | 48BKpyt | 2001 | 100.57, 13.77 |
| AY849074 | Thailand_Bangkok | Human | HM65BK | 1998 | 100.57, 13.77 |
| AY849075 | Thailand_Bangkok | Human | HM75BK | 1998 | 100.57, 13.77 |
| AY849076 | Thailand_Bangkok | Human | HM88BKjj | 1999 | 100.57, 13.77 |
| AY849077 | Thailand_Bangkok | Human | HM208BKpv | 2001 | 100.57, 13.77 |
| AY849112 | Thailand_Bangkok | Dog | 87BKsl | 2001 | 100.57, 13.77 |
| AY219001 | Thailand_Bangkok | Human | HM75 | 1998 | 100.57, 13.77 |
| AB178892 | Thailand_Bangkok | Dog | THA1013 | 1985 | 100.57, 13.77 |
| AB178893 | Thailand_Bangkok | Dog | THA1015 | 1985 | 100.57, 13.77 |
| AB178894 | Thailand_Bangkok | Dog | THA1017 | 1985 | 100.57, 13.77 |
| AB178895 | Thailand_Bangkok | Dog | THA-Abha | 1977 | 100.57, 13.77 |
| AY849162 | Thailand_Buri_Ram | Dog | 485BRpk | 2002 | 103.00.14.90 |
| AY849177 | Thailand_Buri_Ram | Dog | 481BRhr | 2002 | 103.00.14.90 |
| AY849189 | Thailand_Buri_Ram | Dog | 472BRm | 2001 | 103.00.14.90 |
| AY849210 | Thailand_Buri_Ram | Dog | 678cBRm | 2000 | 103.00.14.90 |
| AY849212 | Thailand_Buri_Ram | Dog | 685BRppc | 2002 | 103.00.14.90 |
| AY849114 | Thailand_Chachoengsao | Dog | 182CCbnp | 1999 | 101.40, 13,66 |
| AY849084 | Thailand_Chai_Nat | Dog | 353CNmnr | 2001 | 100.02, 15.13 |
| AY849094 | Thailand_Chai_Nat | Dog | 374CNspy | 2001 | 100.02, 15.13 |
| AY849095 | Thailand_Chai_Nat | Dog | 362CNm | 2001 | 100.02, 15.13 |
| AY849101 | Thailand_Chai_Nat | Dog | 354CNhk | 2001 | 100.02, 15.13 |
| AY849107 | Thailand_Chai_Nat | Dog | 358CNsbr | 2001 | 100.02, 15.13 |
| AY580134 | Thailand_Chaiyaphum | Dog | 38/43 | 2000 | 101.85, 16.03 |
| AY580135 | Thailand_Chaiyaphum | Dog | 39/43 | 2000 | 101.85, 16.03 |
| AY580136 | Thailand_Chaiyaphum | Dog | 46/43 | 2000 | 101.85, 16.03 |
| AY580137 | Thailand_Chaiyaphum | Dog | 33/43 | 2000 | 101.85, 16.03 |
| AY580138 | Thailand_Chaiyaphum | Dog | 19/43 | 2000 | 101.85, 16.03 |
| AY849064 | Thailand_Chaiyaphum | Dog | 38/43 | 2000 | 101.85, 16.03 |
| AY849065 | Thailand_Chaiyaphum | Dog | 39/43 | 2000 | 101.85, 16.03 |
| AY849066 | Thailand_Chaiyaphum | Dog | 46/43 | 2000 | 101.85, 16.03 |
| AY849068 | Thailand_Chaiyaphum | Dog | 33/43 | 2000 | 101.85, 16.03 |
| AY849069 | Thailand_Chaiyaphum | Dog | 34/43 | 2000 | 101.85, 16.03 |
| AY849070 | Thailand_Chaiyaphum | Dog | 19/43 | 2000 | 101.85, 16.03 |
| AY849081 | Thailand_Chaiyaphum | Dog | 294CPm | 2002 | 101.85, 16.03 |
| AY849082 | Thailand_Chaiyaphum | Dog | 295CPksb3 | 2002 | 101.85, 16.03 |
| AY849140 | Thailand_Chaiyaphum | Dog | 296CPksb | 2002 | 101.85, 16.03 |
| AY849130 | Thailand_Chanthaburi | Dog | 22CBkhm | 2001 | 102.10, 12.88 |
| AY849231 | Thailand_Chiang_Mai | Dog | 773CMcp | 2002 | 98.66, 18.83 |
| AY849134 | Thailand_Chon_Buri | Dog | 136CLblm | 1999 | 101.42, 13.29 |
| AY849192 | Thailand_Chon_Buri | Human | HMS241CL | 2001 | 101.42, 13.29 |
| AY849155 | Thailand_Kalasin | Dog | 676KSm | 2002 | 103.62, 16.59 |
| AY849168 | Thailand_Kalasin | Dog | 459KSm | 2002 | 103.62, 16.60 |
| AY849199 | Thailand_Kalasin | Dog | 698KSm | 2002 | 103.62, 16.61 |
| AY849200 | Thailand_Kalasin | Dog | 700KStkt | 2003 | 103.62, 16.62 |
| AY849220 | Thailand_Kalasin | Dog | 732KSm | 2001 | 103.62, 16.63 |
| AY849237 | Thailand_Kamphaeng_Phet | Dog | 802KPlkb | 2002 | 99.53, 16.35 |
| AY580148 | Thailand_Kanchanaburi | Dog | 303KJtmk | 2001 | 99.06, 16.59 |
| AY580149 | Thailand_Kanchanaburi | Dog | 308KJm | 2001 | 99.06, 16.59 |
| AY580150 | Thailand_Kanchanaburi | Dog | 318KJtmk | 2001 | 99.06, 16.59 |
| AY580151 | Thailand_Kanchanaburi | Dog | 326KJtm | 2001 | 99.06, 16.59 |
| AY580157 | Thailand_Kanchanaburi | Dog | 333KJm | 2001 | 99.06, 16.59 |
| AY580158 | Thailand_Kanchanaburi | Dog | 335KJpnt | 2001 | 99.06, 16.59 |
| AY849057 | Thailand_Kanchanaburi | Dog | 303KJtmk | 2001 | 99.06, 16.59 |
| AY849058 | Thailand_Kanchanaburi | Dog | 308KJm | 2001 | 99.06, 16.59 |
| AY849059 | Thailand_Kanchanaburi | Dog | 318KJtmk | 2001 | 99.06, 16.59 |
| AY849060 | Thailand_Kanchanaburi | Dog | 326KJtm | 2001 | 99.06, 16.59 |
| AY849061 | Thailand_Kanchanaburi | Dog | 333KJm | 2001 | 99.06, 16.59 |
| AY849062 | Thailand_Kanchanaburi | Dog | 335KJpnt | 2001 | 99.06, 16.59 |
| AY849176 | Thailand_Khon_Kaen | Dog | 723KKm | 2001 | 102.63, 16.38 |
| AY849205 | Thailand_Khon_Kaen | Dog | 738KKm | 2002 | 102.63, 16.38 |
| AY849218 | Thailand_Khon_Kaen | Dog | 704KKcp | 2000 | 102.63, 16.38 |
| AY849219 | Thailand_Khon_Kaen | Dog | 703KKm | 2000 | 102.63, 16.38 |
| AY849223 | Thailand_Khon_Kaen | Dog | 713KKcp | 2001 | 102.63, 16.38 |
| AY849143 | Thailand_Krabi | Dog | 505KBlt | 2000 | 99.00, 8.18 |
| AY849146 | Thailand_Krabi | Dog | 515KBlt | 2000 | 99.00, 8.18 |
| AY849147 | Thailand_Krabi | Dog | 524KBkn | 2000 | 99.00, 8.18 |
| AY849149 | Thailand_Krabi | Dog | 578KBkt | 2001 | 99.00, 8.18 |
| AY849151 | Thailand_Krabi | Dog | 603KBlt | 2001 | 99.00, 8.18 |
| AY849225 | Thailand_Loei | Dog | 707LYm | 2000 | 101.56, 17.40 |
| AY849091 | Thailand_Lop_Buri | Dog | 393LBm | 2001 | 100.88, 15.11 |
| AY849103 | Thailand_Lop_Buri | Dog | 389LBm | 2001 | 100.88, 15.11 |
| AY849250 | Thailand_Lop_Buri | Dog | 778LBtv | 2003 | 100.88, 15.11 |
| AY849257 | Thailand_Lop_Buri | Dog | 777LBm | 2003 | 100.88, 15.11 |
| AY849203 | Thailand_Mukdahan | Dog | 725MDm | 2001 | 104.50, 16.54 |
| AY849206 | Thailand_Mukdahan | Dog | 742MDm | 2002 | 104.50, 16.55 |
| AY849207 | Thailand_Mukdahan | Dog | 740MDdt | 2002 | 104.50, 16.56 |
| AY849216 | Thailand_Mukdahan | Dog | 705MDm | 2000 | 104.50, 16.57 |
| AY849122 | Thailand_Nakhon_Nayok | Dog | 237NYm | 1999 | 101.15, 14.21 |
| AY580095 | Thailand_Nakhon_Pathom | Dog | 26NPpmt | 2001 | 100.10, 13.92 |
| AY580109 | Thailand_Nakhon_Pathom | Dog | 304NPbl | 2001 | 100.10, 13.92 |
| AY580110 | Thailand_Nakhon_Pathom | Dog | 315NPm | 2001 | 100.10, 13.92 |
| AY580111 | Thailand_Nakhon_Pathom | Dog | 324NPm | 2001 | 100.10, 13.92 |
| AY580114 | Thailand_Nakhon_Pathom | Dog | 13NPsp | 2001 | 100.10, 13.92 |
| AY580146 | Thailand_Nakhon_Pathom | Dog | 232NPsp | 1999 | 100.10, 13.92 |
| AY580154 | Thailand_Nakhon_Pathom | Dog | 215NPncs | 1999 | 100.10, 13.92 |
| AY580155 | Thailand_Nakhon_Pathom | Dog | 263NPm | 1999 | 100.10, 13.92 |
| AY849023 | Thailand_Nakhon_Pathom | Dog | 26NPpmt | 2001 | 100.10, 13.92 |
| AY849039 | Thailand_Nakhon_Pathom | Dog | 304NPbl | 2001 | 100.10, 13.92 |
| AY849040 | Thailand_Nakhon_Pathom | Dog | 315NPm | 2001 | 100.10, 13.92 |
| AY849041 | Thailand_Nakhon_Pathom | Dog | 324NPm | 2001 | 100.10, 13.92 |
| AY849047 | Thailand_Nakhon_Pathom | Dog | 215NPncs | 1999 | 100.10, 13.92 |
| AY849048 | Thailand_Nakhon_Pathom | Dog | 232NPsp | 1999 | 100.10, 13.92 |
| AY849050 | Thailand_Nakhon_Pathom | Dog | 263NPm | 1999 | 100.10, 13.92 |
| AY849124 | Thailand_Nakhon_Ratchasima | Dog | 281NRm | 2001 | 102.10, 15.00 |
| AY849125 | Thailand_Nakhon_Ratchasima | Dog | 282NRpc | 2001 | 102.10, 15.00 |
| AY849142 | Thailand_Nakhon_Ratchasima | Dog | 288NRbl | 2002 | 102.10, 15.00 |
| AY849167 | Thailand_Nakhon_Ratchasima | Dog | 283NRpm | 2001 | 102.10, 15.00 |
| AY849175 | Thailand_Nakhon_Ratchasima | Dog | 289NRht | 2001 | 102.10, 15.00 |
| AY849083 | Thailand_Nakhon_Sawan | Dog | 351NSm | 2001 | 100.23, 15.68 |
| AY849085 | Thailand_Nakhon_Sawan | Dog | 363NScs | 2001 | 100.23, 15.68 |
| AY849088 | Thailand_Nakhon_Sawan | Dog | 361NSly | 2001 | 100.23, 15.68 |
| AY849098 | Thailand_Nakhon_Sawan | Dog | 381NSttk | 2001 | 100.23, 15.68 |
| AY849119 | Thailand_Nakhon_Sawan | Dog | 352NStk | 2001 | 100.23, 15.68 |
| AY849234 | Thailand_Nakhon_Sawan | Dog | 776NStk | 2002 | 100.23, 15.68 |
| AY849235 | Thailand_Nakhon_Sawan | Dog | 795NScs | 2003 | 100.23, 15.68 |
| AY849245 | Thailand_Nakhon_Sawan | Dog | 780NSm | 2003 | 100.23, 15.68 |
| AY849258 | Thailand_Nakhon_Sawan | Dog | 779NSly | 2003 | 100.23, 15.68 |
| AY849093 | Thailand_Nakhon_Si_Thammarat | Dog | 411NTht | 2002 | 99.80, 8.30 |
| AY849145 | Thailand_Nakhon_Si_Thammarat | Dog | 511NTts | 2000 | 99.80, 8.30 |
| AY849163 | Thailand_Nakhon_Si_Thammarat | Dog | 510NTm | 2000 | 99.80, 8.30 |
| AY849166 | Thailand_Nakhon_Si_Thammarat | Dog | 501RNm | 2002 | 99.80, 8.30 |
| AY849173 | Thailand_Nakhon_Si_Thammarat | Dog | 528NTrpb | 2000 | 99.80, 8.30 |
| AY849174 | Thailand_Nakhon_Si_Thammarat | Dog | 548NTlsk | 2000 | 99.80, 8.30 |
| AY849232 | Thailand_Nan | Dog | 774NNm | 2002 | 100.83, 18.88 |
| AY849194 | Thailand_Nong_Khai | Dog | 690NKsps | 2002 | 102.73, 17.77 |
| AY849197 | Thailand_Nong_Khai | Dog | 691NKbk | 2002 | 102.73, 17.77 |
| AY580094 | Thailand_Nonthaburi | Dog | 5NBm | 2001 | 100.40, 13.91 |
| AY580099 | Thailand_Nonthaburi | Dog | 81NBtn | 1999 | 100.40, 13.91 |
| AY580106 | Thailand_Nonthaburi | Dog | 218NBbk | 1999 | 100.40, 13.91 |
| AY580107 | Thailand_Nonthaburi | Dog | 222NBbbt | 1999 | 100.40, 13.91 |
| AY580108 | Thailand_Nonthaburi | Dog | 228NBtn | 1999 | 100.40, 13.91 |
| AY580121 | Thailand_Nonthaburi | Dog | 27NBpk | 2001 | 100.40, 13.91 |
| AY580147 | Thailand_Nonthaburi | Dog | 235NBpk | 1999 | 100.40, 13.91 |
| AY580152 | Thailand_Nonthaburi | Dog | 95NBtn | 1999 | 100.40, 13.91 |
| AY580153 | Thailand_Nonthaburi | Dog | 195NBby | 1999 | 100.40, 13.91 |
| AY849022 | Thailand_Nonthaburi | Dog | 5NBm | 2001 | 100.40, 13.91 |
| AY849027 | Thailand_Nonthaburi | Dog | 81NBtn | 2001 | 100.40, 13.91 |
| AY849036 | Thailand_Nonthaburi | Dog | 218NBbk | 2001 | 100.40, 13.91 |
| AY849037 | Thailand_Nonthaburi | Dog | 222NBbbt | 1999 | 100.40, 13.91 |
| AY849038 | Thailand_Nonthaburi | Dog | 228NBtn | 1999 | 100.40, 13.91 |
| AY849043 | Thailand_Nonthaburi | Dog | 95NBtn | 1999 | 100.40, 13.91 |
| AY849046 | Thailand_Nonthaburi | Dog | 195NBby | 1999 | 100.40, 13.91 |
| AY849049 | Thailand_Nonthaburi | Dog | 235NBpk | 1999 | 100.40, 13.91 |
| AY580131 | Thailand_Pathum_Thani | Dog | 89PTtb | 1999 | 100.67, 14.06 |
| AY849025 | Thailand_Pathum_Thani | Dog | 67PTtyb | 1999 | 100.67, 14.06 |
| AY849026 | Thailand_Pathum_Thani | Dog | 80PTllk | 1999 | 100.67, 14.06 |
| AY849028 | Thailand_Pathum_Thani | Dog | 99PTkl | 1999 | 100.67, 14.06 |
| AY849029 | Thailand_Pathum_Thani | Dog | 79PTm | 1999 | 100.67, 14.06 |
| AY849042 | Thailand_Pathum_Thani | Dog | 91PTns | 1999 | 100.67, 14.06 |
| AY849044 | Thailand_Pathum_Thani | Dog | 108PTllk | 1999 | 100.67, 14.06 |
| AY849045 | Thailand_Pathum_Thani | Dog | 112PTtyb | 1999 | 100.67, 14.06 |
| AY849052 | Thailand_Pathum_Thani | Cat | C269PTm | 2000 | 100.67, 14.06 |
| AY849063 | Thailand_Pathum_Thani | Dog | 89PTtb | 1999 | 100.67, 14.06 |

**Table S1.3 Dataset 5**

| **Accession** | **Location** | **Host** | **Isolate** | **Date** | **Longitude/Latitude** |
| --- | --- | --- | --- | --- | --- |
| EU086167 | Cambodia | Dog | 9908CBG | 1999 | 105.46, 11.40 |
| EU086168 | Cambodia | Dog | 9911CBG | 1998 | 104.68, 10.31 |
| EU086169 | Cambodia | Dog | 9912CBG | 1998 | 104.34, 11.42 |
| EU086170 | Cambodia | Dog | 9914CBG | 1997 | 107.02, 13.75 |
| EU086171 | Cambodia | Dog | 9916CBG | 1999 | 104.18, 10.61 |
| EU086172 | Cambodia | Dog | 02006CBG | 1998 |  |
| EU159391 | China_Anhui | Dog | H69 | 1969 | 117.20, 31.82 |
| EU159396 | China_Anhui | Dog | H | 1989 | 117.20, 31.82 |
| EU159400 | China_Anhui | Dog | H89 | 1989 | 117.20, 31.82 |
| HQ118104 | China_Anhui | Dog | HeFei | 1989 | 117.20, 31.82 |
| EU159379 | China_Anhui | Dog | FEIDONG | 1989 | 117.20, 31.82 |
| EU086185 | China_Chongqing | Human | 02050CHI | 1992 | 107.60, 29.58 |
| DQ866111 | China_Guangxi | Dog | GXN119 | 2000 | 108.58, 23.64 |
| FJ594278 | China_Guangxi | Dog | N11 | 1997 | 108.58, 23.64 |
| EF990579 | China_Guangxi | Dog | CGX0602D | 2006 | 108.58, 23.64 |
| JN974827 | China_Guangxi | Dog | CGX0606D | 2006 | 108.58, 23.64 |
| DQ666287 | China_Guangxi | Dog | Guangxi_Yl66 | 2004 | 108.58, 23.64 |
| DQ866107 | China_Guangxi | Dog | GX074 | 2003 | 108.58, 23.64 |
| DQ866118 | China_Guangxi | Dog | GXBS | 2005 | 108.58, 23.64 |
| EU159399 | China_Guangxi | Dog | Yue2 | 1994 | 108.58, 23.64 |
| GQ472478 | China_Guangxi | Dog | GXYZD | 2007 | 108.58, 23.64 |
| EU086175 | China_Guangxi | Dog | 02037CHI | 1997 | 108.58, 23.64 |
| EF990578 | China_Guangxi | Dog | CGX0601D | 2006 | 108.58, 23.64 |
| JN974828 | China_Guangxi | Dog | CGX0625D | 2006 | 108.58, 23.64 |
| DQ866105 | China_Guangxi | Dog | GX01 | 2004 | 108.58, 23.64 |
| DQ866108 | China_Guangxi | Dog | GX08 | 2003 | 108.58, 23.64 |
| DQ866114 | China_Guangxi | Dog | GX260 | 2004 | 108.58, 23.64 |
| DQ866119 | China_Guangxi | Dog | GXHX | 2005 | 108.58, 23.64 |
| DQ866121 | China_Guangxi | Dog | GXWXp | 2005 | 108.58, 23.64 |
| GU358653 | China_Guangxi | Dog | GX4 | 1994 | 108.58, 23.64 |
| GQ472468 | China_Guangxi | Dog | GXHXB | 2007 | 108.58, 23.64 |
| GQ472470 | China_Guangxi | Dog | GXLB | 2007 | 108.58, 23.64 |
| GQ472477 | China_Guangxi | Dog | GXQZD | 2006 | 108.58, 23.64 |
| EU086182 | China_Guangxi | Dog | 02046CHI | 1994 | 108.58, 23.64 |
| EU086183 | China_Guangxi | Dog | 02047CHI | 1994 | 108.58, 23.64 |
| HM486370 | China_Guangxi | Dog | CGX0516D | 2005 | 108.58, 23.64 |
| DQ866112 | China_Guangxi | Dog | GX195 | 2004 | 108.58, 23.64 |
| GQ472469 | China_Guangxi | Dog | GXLA11 | 2007 | 108.58, 23.64 |
| GQ472471 | China_Guangxi | Dog | GXLCC | 2007 | 108.58, 23.64 |
| EU159390 | China_Guangxi | Dog | FS | 1998 | 108.58, 23.64 |
| EU159385 | China_Guangxi | Dog | Yue1 | 1997 | 108.58, 23.64 |
| DQ666293 | China_Guizhou | Dog | Guizhou_A173 | 2004 | 107.22, 26.78 |
| JN974832 | China_Guizhou | Human | CGZ0924H | 2009 | 107.22, 26.78 |
| DQ666291 | China_Guizhou | Dog | Guizhou_A148 | 2004 | 107.22, 26.78 |
| DQ666294 | China_Guizhou | Dog | Guizhou_Qx1 | 2004 | 107.22, 26.78 |
| HQ118102 | China_Guizhou | Dog | gk5 | 2006 | 107.22, 26.78 |
| HM486369 | China_Guizhou | Dog | CGZ0620D | 2006 | 107.22, 26.78 |
| HM486368 | China_Guizhou | Dog | CGZ0508D | 2005 | 107.22, 26.78 |
| DQ666290 | China_Guizhou | Dog | Guizhou_A103 | 2004 | 107.22, 26.78 |
| JN974830 | China_Guizhou | Dog | CGZ0501D | 2005 | 107.22, 26.78 |
| EU159395 | China_Guizhou | Dog | gg4 | 2006 | 107.22, 26.78 |
| DQ666288 | China_Guizhou | Dog | Guizhou_A10 | 2004 | 107.22, 26.78 |
| EU159392 | China_Shanghai | Human | SBH | 1992 | 121.42, 31.16 |
| JN974836 | China_Hunan | Dog | CHN0504D | 2005 | 111.84, 27.72 |
| JN974846 | China_Hunan | Dog | CHN0809D | 2008 | 111.84, 27.72 |
| DQ666308 | China_Hunan | Dog | Hunan_Wg12 | 2004 | 111.84, 27.72 |
| HM486363 | China_Jiangsu | Dog | CJS0634D | 2006 | 119.92, 32.84 |
| EU086173 | China_Jiangsu | Dog | 9811CHI | 1998 | 119.92, 32.84 |
| JN974854 | China_Jiangxi | Dog | CJX0902D | 2009 | 115.57, 27.56 |
| JN974855 | China_Jiangxi | Dog | CJX0903D | 2009 | 115.57, 27.57 |
| DQ875051 | China_Jilin | Deer | DRV | 1989 | 125.40, 43.88 |
| JN974872 | China_Shanghai | Dog | CSH0329D | 2003 | 121.42, 31.16 |
| JN974874 | China_Shanghai | Dog | CSH0501D | 2005 | 121.42, 31.16 |
| EU086176 | China_Shanghai | Human | 02040CHI | 1992 | 121.42, 31.16 |
| EU159393 | China_Shanghai | Dog | SBD | 1992 | 121.42, 31.16 |
| GU591790 | China_Sichuan | Dog | Sichuan-BZ-1 | 2009 | 102.60, 30.63 |
| JN974859 | China_Sichuan | Dog | CSC0910D | 2009 | 102.60, 30.63 |
| EU275243 | China_Yunnan | Dog | Yunnan_Tc06 | 2006 | 101.42, 24.32 |
| JQ730682 | China_Yunnan | Dog | CYN1009D | 2010 | 101.42, 24.32 |
| JF819623 | China_Yunnan | Dog | CYN1006D | 2010 | 101.42, 24.32 |
| JF819619 | China_Yunnan | Dog | CYN1003D | 2010 | 101.42, 24.32 |
| JF819611 | China_Yunnan | Dog | CYN0919D | 2009 | 101.42, 24.32 |
| JQ040596 | China_Yunnan | Human | CYN1025H | 2010 | 101.42, 24.32 |
| EU095330 | China_Yunnan | Dog | Yunnan_Md06 | 2006 | 101.42, 24.32 |
| EU275244 | China_Yunnan | Dog | Yunnan_Zt07 | 2007 | 101.42, 24.32 |
| EU275245 | China_Yunnan | Dog | Yunnan_Qj07 | 2007 | 101.42, 24.32 |
| JF819605 | China_Yunnan | Dog | CYN0811D | 2008 | 101.42, 24.32 |
| JF819612 | China_Yunnan | Human | CYN0924H | 2009 | 101.42, 24.32 |
| JF819615 | China_Yunnan | Dog | CYN0814D | 2008 | 101.42, 24.32 |
| JF819614 | China_Yunnan | Human | CYN0921HN | 2009 | 101.42, 24.32 |
| EU282381 | China_Yunnan | Dog | CHVC06 | 2006 | 101.42, 24.32 |
| JN974878 | China_Zhejiang | Dog | CZJ0814D | 2008 | 120.05, 29.42 |
| EU159397 | China_Zhejiang | Dog | LH | 2006 | 120.05, 29.42 |
| HQ118107 | China_Zhejiang | Dog | ZJD3 | 2008 | 120.05, 29.42 |
| EU086193 | Laos_Vientiane | Dog | 9910LAO | 1999 | 102.62, 17.98 |
| EU086194 | Laos_Vientiane | Dog | 02001LAO | 2002 | 102.62, 17.98 |
| EU086195 | Laos_Vientiane | Dog | 02002LAO | 2002 | 102.62, 17.98 |
| EU086164 | Myanmar_Yangon | Dog | 9909BIR | 1999 | 96.15, 16.90 |
| EU086165 | Myanmar_Yangon | Dog | 9913BIR | 1999 | 96.15, 16.90 |
| EU086166 | Myanmar_Yangon | Dog | 9915BIR | 1999 | 96.15, 16.90 |
| EU086200 | Philippines | Dog | 94270PHI | 1994 |  |
| EU086201 | Philippines | Dog | 94273PHI | 1994 |  |
| EU086202 | Philippines | Dog | 94280PHI | 1994 |  |
| EU086203 | Philippines | Human | 03006PHI | 2000 |  |
| EU086204 | Philippines | Human | 03007PHI | 2001 |  |
| EU086205 | Philippines | Human | 04030PHI | 2004 |  |
| AY138572 | Thailand | Dog | 2908 | 1995 |  |
| AY580089 | Thailand | Dog | THA8738 |  |  |
| DQ267925 | Thailand | Dog | D664_45 |  |  |
| EU086206 | Thailand | Human | 8734THA | 1983 |  |
| EU086207 | Thailand | Human | 8743THA | 1983 |  |
| EU086208 | Thailand | Human | 8738THA | 1983 |  |
| EU293111 | Thailand | Human | 8764THA | 1983 |  |
| EU293121 | Thailand |  | 8743THA |  |  |
| GQ303555 | Thailand | Human | UA341 | 2009 |  |
| GQ303556 | Thailand | Dog | D9/52 | 2009 |  |
| GU992307 | Thailand | Dog | 8764THA | 1983 |  |
| U22653 | Thailand |  | 8738THA |  |  |
| AY849164 | Thailand_Amnat_Chareon | Dog | 486UMhsp | 2002 | 104.76, 15.90 |
| AY849165 | Thailand_Amnat_Charoen | Dog | 487UMm | 2002 | 104.76, 15.90 |
| AY849187 | Thailand_Amnat_Charoen | Dog | 488UMm | 1998 | 104.76, 15.90 |
| AY849195 | Thailand_Amnat_Charoen | Dog | 689Umm | 2001 | 104.76, 15.90 |
| AY849196 | Thailand_Amnat_Charoen | Dog | 688Umm | 2002 | 104.76, 15.90 |
| AY849123 | Thailand_Ang_Thong | Dog | 270ATvsc | 2001 | 100.30, 14.70 |
| AB178896 | Thailand_Ayudhaya | Dog | THA-AY | 1963 | 100.60, 14,02 |
| AY849128 | Thailand_Ayutthaya | Dog | 191AYsn | 1999 | 100.60, 14,02 |
| AY849136 | Thailand_Ayutthaya | Dog | 250AYbt | 1999 | 100.60, 14,02 |
| AY218997 | Thailand_Bangkok | Dog | D48 |  | 100.57, 13.77 |
| AY218998 | Thailand_Bangkok | Dog | D51 |  | 100.57, 13.78 |
| AY218999 | Thailand_Bangkok | Human | HM208 |  | 100.57, 13.79 |
| AY219000 | Thailand_Bangkok | Human | HM65 |  | 100.57, 13.80 |
| AY219002 | Thailand_Bangkok | Human | HM88 |  | 100.57, 13.81 |
| AY580090 | Thailand_Bangkok | Human | HM88BKjj | 1999 | 100.57, 13.77 |
| AY580091 | Thailand_Bangkok | Human | HM75BK | 1998 | 100.57, 13.77 |
| AY580092 | Thailand_Bangkok | Human | HM208BKpv | 2001 | 100.57, 13.77 |
| AY580112 | Thailand_Bangkok | Dog | 6BKjt | 2001 | 100.57, 13.77 |
| AY580117 | Thailand_Bangkok | Dog | 16BKwtl | 2001 | 100.57, 13.77 |
| AY580118 | Thailand_Bangkok | Dog | 19BKbkp | 2001 | 100.57, 13.77 |
| AY580119 | Thailand_Bangkok | Dog | 20Bkds | 2001 | 100.57, 13.77 |
| AY580122 | Thailand_Bangkok | Dog | 35BKbk | 2001 | 100.57, 13.77 |
| AY580123 | Thailand_Bangkok | Dog | 36BKpsc | 2001 | 100.57, 13.77 |
| AY580124 | Thailand_Bangkok | Dog | 38BKlp | 2001 | 100.57, 13.77 |
| AY580125 | Thailand_Bangkok | Dog | 40BKpp | 2001 | 100.57, 13.77 |
| AY580126 | Thailand_Bangkok | Dog | 48BKpyt | 2001 | 100.57, 13.77 |
| AY580127 | Thailand_Bangkok | Dog | 51BKds | 2001 | 100.57, 13.77 |
| AY580129 | Thailand_Bangkok | Dog | 70BKdd | 2001 | 100.57, 13.77 |
| AY580133 | Thailand_Bangkok | Human | HM65BK | 1998 | 100.57, 13.77 |
| AY580141 | Thailand_Bangkok | Cat | C267BKbkn | 2001 | 100.57, 13.77 |
| AY580142 | Thailand_Bangkok | Cat | C271BKrtv | 2001 | 100.57, 13.77 |
| AY580143 | Thailand_Bangkok | Cat | C274BKdd | 1999 | 100.57, 13.77 |
| AY580144 | Thailand_Bangkok | Cat | C276BKpv | 1999 | 100.57, 13.77 |
| AY580145 | Thailand_Bangkok | Cat | C277BKkt | 1999 | 100.57, 13.77 |
| AY849051 | Thailand_Bangkok | Cat | C267BKbkn | 2000 | 100.57, 13.77 |
| AY849053 | Thailand_Bangkok | Cat | C271BKrtv | 2001 | 100.57, 13.77 |
| AY849054 | Thailand_Bangkok | Cat | C274BKdd | 1999 | 100.57, 13.77 |
| AY849055 | Thailand_Bangkok | Cat | C276BKpv | 1999 | 100.57, 13.77 |
| AY849056 | Thailand_Bangkok | Cat | C277BKkt | 1999 | 100.57, 13.77 |
| AY849071 | Thailand_Bangkok | Dog | 51BKds | 1999 | 100.57, 13.77 |
| AY849073 | Thailand_Bangkok | Dog | 48BKpyt | 2001 | 100.57, 13.77 |
| AY849074 | Thailand_Bangkok | Human | HM65BK | 1998 | 100.57, 13.77 |
| AY849075 | Thailand_Bangkok | Human | HM75BK | 1998 | 100.57, 13.77 |
| AY849076 | Thailand_Bangkok | Human | HM88BKjj | 1999 | 100.57, 13.77 |
| AY849077 | Thailand_Bangkok | Human | HM208BKpv | 2001 | 100.57, 13.77 |
| AY849112 | Thailand_Bangkok | Dog | 87BKsl | 2001 | 100.57, 13.77 |
| AY219001 | Thailand_Bangkok | Human | HM75 | 1998 | 100.57, 13.77 |
| AB178892 | Thailand_Bangkok | Dog | THA1013 | 1985 | 100.57, 13.77 |
| AB178893 | Thailand_Bangkok | Dog | THA1015 | 1985 | 100.57, 13.77 |
| AB178894 | Thailand_Bangkok | Dog | THA1017 | 1985 | 100.57, 13.77 |
| AB178895 | Thailand_Bangkok | Dog | THA-Abha | 1977 | 100.57, 13.77 |
| AY849162 | Thailand_Buri_Ram | Dog | 485BRpk | 2002 | 103.00.14.90 |
| AY849177 | Thailand_Buri_Ram | Dog | 481BRhr | 2002 | 103.00.14.90 |
| AY849189 | Thailand_Buri_Ram | Dog | 472BRm | 2001 | 103.00.14.90 |
| AY849210 | Thailand_Buri_Ram | Dog | 678cBRm | 2000 | 103.00.14.90 |
| AY849212 | Thailand_Buri_Ram | Dog | 685BRppc | 2002 | 103.00.14.90 |
| AY849114 | Thailand_Chachoengsao | Dog | 182CCbnp | 1999 | 101.40, 13,66 |
| AY849084 | Thailand_Chai_Nat | Dog | 353CNmnr | 2001 | 100.02, 15.13 |
| AY849094 | Thailand_Chai_Nat | Dog | 374CNspy | 2001 | 100.02, 15.13 |
| AY849095 | Thailand_Chai_Nat | Dog | 362CNm | 2001 | 100.02, 15.13 |
| AY849101 | Thailand_Chai_Nat | Dog | 354CNhk | 2001 | 100.02, 15.13 |
| AY849107 | Thailand_Chai_Nat | Dog | 358CNsbr | 2001 | 100.02, 15.13 |
| AY580134 | Thailand_Chaiyaphum | Dog | 38/43 | 2000 | 101.85, 16.03 |
| AY580135 | Thailand_Chaiyaphum | Dog | 39/43 | 2000 | 101.85, 16.03 |
| AY580136 | Thailand_Chaiyaphum | Dog | 46/43 | 2000 | 101.85, 16.03 |
| AY580137 | Thailand_Chaiyaphum | Dog | 33/43 | 2000 | 101.85, 16.03 |
| AY580138 | Thailand_Chaiyaphum | Dog | 19/43 | 2000 | 101.85, 16.03 |
| AY849064 | Thailand_Chaiyaphum | Dog | 38/43 | 2000 | 101.85, 16.03 |
| AY849065 | Thailand_Chaiyaphum | Dog | 39/43 | 2000 | 101.85, 16.03 |
| AY849066 | Thailand_Chaiyaphum | Dog | 46/43 | 2000 | 101.85, 16.03 |
| AY849068 | Thailand_Chaiyaphum | Dog | 33/43 | 2000 | 101.85, 16.03 |
| AY849069 | Thailand_Chaiyaphum | Dog | 34/43 | 2000 | 101.85, 16.03 |
| AY849070 | Thailand_Chaiyaphum | Dog | 19/43 | 2000 | 101.85, 16.03 |
| AY849081 | Thailand_Chaiyaphum | Dog | 294CPm | 2002 | 101.85, 16.03 |
| AY849082 | Thailand_Chaiyaphum | Dog | 295CPksb3 | 2002 | 101.85, 16.03 |
| AY849140 | Thailand_Chaiyaphum | Dog | 296CPksb | 2002 | 101.85, 16.03 |
| AY849130 | Thailand_Chanthaburi | Dog | 22CBkhm | 2001 | 102.10, 12.88 |
| AY849231 | Thailand_Chiang_Mai | Dog | 773CMcp | 2002 | 98.66, 18.83 |
| AY849134 | Thailand_Chon_Buri | Dog | 136CLblm | 1999 | 101.42, 13.29 |
| AY849192 | Thailand_Chon_Buri | Human | HMS241CL | 2001 | 101.42, 13.29 |
| AY849155 | Thailand_Kalasin | Dog | 676KSm | 2002 | 103.62, 16.59 |
| AY849168 | Thailand_Kalasin | Dog | 459KSm | 2002 | 103.62, 16.60 |
| AY849199 | Thailand_Kalasin | Dog | 698KSm | 2002 | 103.62, 16.61 |
| AY849200 | Thailand_Kalasin | Dog | 700KStkt | 2003 | 103.62, 16.62 |
| AY849220 | Thailand_Kalasin | Dog | 732KSm | 2001 | 103.62, 16.63 |
| AY849237 | Thailand_Kamphaeng_Phet | Dog | 802KPlkb | 2002 | 99.53, 16.35 |
| AY580148 | Thailand_Kanchanaburi | Dog | 303KJtmk | 2001 | 99.06, 16.59 |
| AY580149 | Thailand_Kanchanaburi | Dog | 308KJm | 2001 | 99.06, 16.59 |
| AY580150 | Thailand_Kanchanaburi | Dog | 318KJtmk | 2001 | 99.06, 16.59 |
| AY580151 | Thailand_Kanchanaburi | Dog | 326KJtm | 2001 | 99.06, 16.59 |
| AY580157 | Thailand_Kanchanaburi | Dog | 333KJm | 2001 | 99.06, 16.59 |
| AY580158 | Thailand_Kanchanaburi | Dog | 335KJpnt | 2001 | 99.06, 16.59 |
| AY849057 | Thailand_Kanchanaburi | Dog | 303KJtmk | 2001 | 99.06, 16.59 |
| AY849058 | Thailand_Kanchanaburi | Dog | 308KJm | 2001 | 99.06, 16.59 |
| AY849059 | Thailand_Kanchanaburi | Dog | 318KJtmk | 2001 | 99.06, 16.59 |
| AY849060 | Thailand_Kanchanaburi | Dog | 326KJtm | 2001 | 99.06, 16.59 |
| AY849061 | Thailand_Kanchanaburi | Dog | 333KJm | 2001 | 99.06, 16.59 |
| AY849062 | Thailand_Kanchanaburi | Dog | 335KJpnt | 2001 | 99.06, 16.59 |
| AY849176 | Thailand_Khon_Kaen | Dog | 723KKm | 2001 | 102.63, 16.38 |
| AY849205 | Thailand_Khon_Kaen | Dog | 738KKm | 2002 | 102.63, 16.38 |
| AY849218 | Thailand_Khon_Kaen | Dog | 704KKcp | 2000 | 102.63, 16.38 |
| AY849219 | Thailand_Khon_Kaen | Dog | 703KKm | 2000 | 102.63, 16.38 |
| AY849223 | Thailand_Khon_Kaen | Dog | 713KKcp | 2001 | 102.63, 16.38 |
| AY849143 | Thailand_Krabi | Dog | 505KBlt | 2000 | 99.00, 8.18 |
| AY849146 | Thailand_Krabi | Dog | 515KBlt | 2000 | 99.00, 8.18 |
| AY849147 | Thailand_Krabi | Dog | 524KBkn | 2000 | 99.00, 8.18 |
| AY849149 | Thailand_Krabi | Dog | 578KBkt | 2001 | 99.00, 8.18 |
| AY849151 | Thailand_Krabi | Dog | 603KBlt | 2001 | 99.00, 8.18 |
| AY849225 | Thailand_Loei | Dog | 707LYm | 2000 | 101.56, 17.40 |
| AY849091 | Thailand_Lop_Buri | Dog | 393LBm | 2001 | 100.88, 15.11 |
| AY849103 | Thailand_Lop_Buri | Dog | 389LBm | 2001 | 100.88, 15.11 |
| AY849250 | Thailand_Lop_Buri | Dog | 778LBtv | 2003 | 100.88, 15.11 |
| AY849257 | Thailand_Lop_Buri | Dog | 777LBm | 2003 | 100.88, 15.11 |
| AY849203 | Thailand_Mukdahan | Dog | 725MDm | 2001 | 104.50, 16.54 |
| AY849206 | Thailand_Mukdahan | Dog | 742MDm | 2002 | 104.50, 16.55 |
| AY849207 | Thailand_Mukdahan | Dog | 740MDdt | 2002 | 104.50, 16.56 |
| AY849216 | Thailand_Mukdahan | Dog | 705MDm | 2000 | 104.50, 16.57 |
| AY849122 | Thailand_Nakhon_Nayok | Dog | 237NYm | 1999 | 101.15, 14.21 |
| AY580095 | Thailand_Nakhon_Pathom | Dog | 26NPpmt | 2001 | 100.10, 13.92 |
| AY580109 | Thailand_Nakhon_Pathom | Dog | 304NPbl | 2001 | 100.10, 13.92 |
| AY580110 | Thailand_Nakhon_Pathom | Dog | 315NPm | 2001 | 100.10, 13.92 |
| AY580111 | Thailand_Nakhon_Pathom | Dog | 324NPm | 2001 | 100.10, 13.92 |
| AY580114 | Thailand_Nakhon_Pathom | Dog | 13NPsp | 2001 | 100.10, 13.92 |
| AY580146 | Thailand_Nakhon_Pathom | Dog | 232NPsp | 1999 | 100.10, 13.92 |
| AY580154 | Thailand_Nakhon_Pathom | Dog | 215NPncs | 1999 | 100.10, 13.92 |
| AY580155 | Thailand_Nakhon_Pathom | Dog | 263NPm | 1999 | 100.10, 13.92 |
| AY849023 | Thailand_Nakhon_Pathom | Dog | 26NPpmt | 2001 | 100.10, 13.92 |
| AY849039 | Thailand_Nakhon_Pathom | Dog | 304NPbl | 2001 | 100.10, 13.92 |
| AY849040 | Thailand_Nakhon_Pathom | Dog | 315NPm | 2001 | 100.10, 13.92 |
| AY849041 | Thailand_Nakhon_Pathom | Dog | 324NPm | 2001 | 100.10, 13.92 |
| AY849047 | Thailand_Nakhon_Pathom | Dog | 215NPncs | 1999 | 100.10, 13.92 |
| AY849048 | Thailand_Nakhon_Pathom | Dog | 232NPsp | 1999 | 100.10, 13.92 |
| AY849050 | Thailand_Nakhon_Pathom | Dog | 263NPm | 1999 | 100.10, 13.92 |
| AY849124 | Thailand_Nakhon_Ratchasima | Dog | 281NRm | 2001 | 102.10, 15.00 |
| AY849125 | Thailand_Nakhon_Ratchasima | Dog | 282NRpc | 2001 | 102.10, 15.00 |
| AY849142 | Thailand_Nakhon_Ratchasima | Dog | 288NRbl | 2002 | 102.10, 15.00 |
| AY849167 | Thailand_Nakhon_Ratchasima | Dog | 283NRpm | 2001 | 102.10, 15.00 |
| AY849175 | Thailand_Nakhon_Ratchasima | Dog | 289NRht | 2001 | 102.10, 15.00 |
| AY849083 | Thailand_Nakhon_Sawan | Dog | 351NSm | 2001 | 100.23, 15.68 |
| AY849085 | Thailand_Nakhon_Sawan | Dog | 363NScs | 2001 | 100.23, 15.68 |
| AY849088 | Thailand_Nakhon_Sawan | Dog | 361NSly | 2001 | 100.23, 15.68 |
| AY849098 | Thailand_Nakhon_Sawan | Dog | 381NSttk | 2001 | 100.23, 15.68 |
| AY849119 | Thailand_Nakhon_Sawan | Dog | 352NStk | 2001 | 100.23, 15.68 |
| AY849234 | Thailand_Nakhon_Sawan | Dog | 776NStk | 2002 | 100.23, 15.68 |
| AY849235 | Thailand_Nakhon_Sawan | Dog | 795NScs | 2003 | 100.23, 15.68 |
| AY849245 | Thailand_Nakhon_Sawan | Dog | 780NSm | 2003 | 100.23, 15.68 |
| AY849258 | Thailand_Nakhon_Sawan | Dog | 779NSly | 2003 | 100.23, 15.68 |
| AY849093 | Thailand_Nakhon_Si_Thammarat | Dog | 411NTht | 2002 | 99.80, 8.30 |
| AY849145 | Thailand_Nakhon_Si_Thammarat | Dog | 511NTts | 2000 | 99.80, 8.30 |
| AY849163 | Thailand_Nakhon_Si_Thammarat | Dog | 510NTm | 2000 | 99.80, 8.30 |
| AY849166 | Thailand_Nakhon_Si_Thammarat | Dog | 501RNm | 2002 | 99.80, 8.30 |
| AY849173 | Thailand_Nakhon_Si_Thammarat | Dog | 528NTrpb | 2000 | 99.80, 8.30 |
| AY849174 | Thailand_Nakhon_Si_Thammarat | Dog | 548NTlsk | 2000 | 99.80, 8.30 |
| AY849232 | Thailand_Nan | Dog | 774NNm | 2002 | 100.83, 18.88 |
| AY849194 | Thailand_Nong_Khai | Dog | 690NKsps | 2002 | 102.73, 17.77 |
| AY849197 | Thailand_Nong_Khai | Dog | 691NKbk | 2002 | 102.73, 17.77 |
| AY580094 | Thailand_Nonthaburi | Dog | 5NBm | 2001 | 100.40, 13.91 |
| AY580099 | Thailand_Nonthaburi | Dog | 81NBtn | 1999 | 100.40, 13.91 |
| AY580106 | Thailand_Nonthaburi | Dog | 218NBbk | 1999 | 100.40, 13.91 |
| AY580107 | Thailand_Nonthaburi | Dog | 222NBbbt | 1999 | 100.40, 13.91 |
| AY580108 | Thailand_Nonthaburi | Dog | 228NBtn | 1999 | 100.40, 13.91 |
| AY580121 | Thailand_Nonthaburi | Dog | 27NBpk | 2001 | 100.40, 13.91 |
| AY580147 | Thailand_Nonthaburi | Dog | 235NBpk | 1999 | 100.40, 13.91 |
| AY580152 | Thailand_Nonthaburi | Dog | 95NBtn | 1999 | 100.40, 13.91 |
| AY580153 | Thailand_Nonthaburi | Dog | 195NBby | 1999 | 100.40, 13.91 |
| AY849022 | Thailand_Nonthaburi | Dog | 5NBm | 2001 | 100.40, 13.91 |
| AY849027 | Thailand_Nonthaburi | Dog | 81NBtn | 2001 | 100.40, 13.91 |
| AY849036 | Thailand_Nonthaburi | Dog | 218NBbk | 2001 | 100.40, 13.91 |
| AY849037 | Thailand_Nonthaburi | Dog | 222NBbbt | 1999 | 100.40, 13.91 |
| AY849038 | Thailand_Nonthaburi | Dog | 228NBtn | 1999 | 100.40, 13.91 |
| AY849043 | Thailand_Nonthaburi | Dog | 95NBtn | 1999 | 100.40, 13.91 |
| AY849046 | Thailand_Nonthaburi | Dog | 195NBby | 1999 | 100.40, 13.91 |
| AY849049 | Thailand_Nonthaburi | Dog | 235NBpk | 1999 | 100.40, 13.91 |
| AY580131 | Thailand_Pathum_Thani | Dog | 89PTtb | 1999 | 100.67, 14.06 |
| AY849025 | Thailand_Pathum_Thani | Dog | 67PTtyb | 1999 | 100.67, 14.06 |
| AY849026 | Thailand_Pathum_Thani | Dog | 80PTllk | 1999 | 100.67, 14.06 |
| AY849028 | Thailand_Pathum_Thani | Dog | 99PTkl | 1999 | 100.67, 14.06 |
| AY849029 | Thailand_Pathum_Thani | Dog | 79PTm | 1999 | 100.67, 14.06 |
| AY849042 | Thailand_Pathum_Thani | Dog | 91PTns | 1999 | 100.67, 14.06 |
| AY849044 | Thailand_Pathum_Thani | Dog | 108PTllk | 1999 | 100.67, 14.06 |
| AY849045 | Thailand_Pathum_Thani | Dog | 112PTtyb | 1999 | 100.67, 14.06 |
| AY849052 | Thailand_Pathum_Thani | Cat | C269PTm | 2000 | 100.67, 14.06 |
| AY849063 | Thailand_Pathum_Thani | Dog | 89PTtb | 1999 | 100.67, 14.06 |
| AY849113 | Thailand_Pathum_Thani | Dog | 156PTns | 1999 | 100.67, 14.06 |
| AY580097 | Thailand_Pathum_Thani | Dog | 67PTtyb | 2001 | 100.67, 14.06 |
| AY580098 | Thailand_Pathum_Thani | Dog | 80PTllk | 1999 | 100.67, 14.06 |
| AY580113 | Thailand_Pathum_Thani | Dog | 8PTm | 2001 | 100.67, 14.06 |
| AY580116 | Thailand_Pathum_Thani | Dog | 15PTllk | 2001 | 100.67, 14.06 |
| AY580130 | Thailand_Pathum_Thani | Dog | 79PTm | 1999 | 100.67, 14.06 |
| AY580139 | Thailand_Pathum_Thani | Dog | 108PTllk | 1999 | 100.67, 14.06 |
| AY580140 | Thailand_Pathum_Thani | Dog | 112PTtyb | 1999 | 100.67, 14.06 |
| AY580156 | Thailand_Pathum_Thani | Cat | C269PTm | 1999 | 100.67, 14.06 |
| AY849228 | Thailand_Phangnga | Dog | 747PGtp | 2003 | 98.41, 8.64 |
| AY849078 | Thailand_Phatthalung | Dog | 404PLkcs | 2002 | 100.06, 7.51 |
| AY849080 | Thailand_Phatthalung | Dog | 415PLtm | 2002 | 100.06, 7.51 |
| AY849154 | Thailand_Phatthalung | Dog | 656PLppy | 2002 | 100.06, 7.51 |
| AY849156 | Thailand_Phatthalung | Dog | 513PLm | 2000 | 100.06, 7.51 |
| AY849233 | Thailand_Phayao | Dog | 775PYp | 2003 | 100.06, 19.21 |
| AY849229 | Thailand_Phetchabun | Dog | 766PRm | 2003 | 101.00, 16.22 |
| AY849230 | Thailand_Phetchabun | Dog | 769PRcd | 2003 | 101.00, 16.22 |
| AY849243 | Thailand_Phetchabun | Dog | 815PRls | 2002 | 101.00, 16.22 |
| AY849246 | Thailand_Phetchabun | Dog | 762PRm | 2003 | 101.00, 16.22 |
| AY849249 | Thailand_Phetchabun | Dog | 816PRcd | 2002 | 101.00, 16.22 |
| AY849115 | Thailand_Phetchaburi | Dog | 349PBm | 2001 | 101.00, 16.22 |
| AY849117 | Thailand_Phetchaburi | Dog | 329PBm | 2001 | 101.00, 16.22 |
| AY849132 | Thailand_Phetchaburi | Dog | 319PBm | 2001 | 101.00, 16.22 |
| AY849141 | Thailand_Phetchaburi | Dog | 313PBnyp | 2001 | 101.00, 16.22 |
| AY849131 | Thailand_Phichit | Dog | 162PCm | 1999 | 100.35, 16.26 |
| AY849244 | Thailand_Phichit | Dog | 805PCbmn | 2002 | 100.35, 16.26 |
| AY849102 | Thailand_Phisanulok | Dog | 425PN | 2002 | 100.50, 16.99 |
| AY849104 | Thailand_Phisanulok | Dog | 423PN | 2002 | 100.50, 16.99 |
| AY849105 | Thailand_Phisanulok | Dog | 424PN | 2002 | 100.50, 16.99 |
| AY849139 | Thailand_Phisanulok | Dog | 426PN | 2002 | 100.50, 16.99 |
| AY849239 | Thailand_Phisanulok | Dog | 806PNnm | 2002 | 100.50, 16.99 |
| AY849247 | Thailand_Phisanulok | Dog | 813PNbrk | 2002 | 100.50, 16.99 |
| AY849248 | Thailand_Phisanulok | Dog | 796PNbrk | 2003 | 100.50, 16.99 |
| AY849109 | Thailand_Prachuap_Khiri_Khan | Dog | 334PJsry | 2001 | 99.73, 12.26 |
| AY849121 | Thailand_Prachuap_Khiri_Khan | Dog | 157PJsry | 1999 | 99.73, 12.26 |
| AY849260 | Thailand_Prachuap_Khiri_Khan | Dog | 338PJm | 2001 | 99.73, 12.26 |
| AY849153 | Thailand_Ranong | Dog | 493RNm | 2000 | 98.72, 10.03 |
| AY849178 | Thailand_Ranong | Dog | 494RNm | 2000 | 98.72, 10.03 |
| AY849185 | Thailand_Ranong | Dog | 495RNm | 2001 | 98.72, 10.03 |
| AY849116 | Thailand_Ratchaburi | Dog | 307RBptr | 2001 | 99.57, 13.52 |
| AY849120 | Thailand_Ratchaburi | Dog | 301RBm | 2001 | 99.57, 13.52 |
| AY849126 | Thailand_Ratchaburi | Dog | 305RBbp | 2001 | 99.57, 13.52 |
| AY849127 | Thailand_Ratchaburi | Dog | 306RBptr | 2001 | 99.57, 13.52 |
| AY849137 | Thailand_Ratchaburi | Dog | 302RBpt | 2001 | 99.57, 13.52 |
| AY849227 | Thailand_Rayong | Human | HMS223RY | 2002 | 101.42, 12.83 |
| AY849184 | Thailand_Roi_Et | Dog | 454LAm | 2001 | 103.77, 15.92 |
| AY849186 | Thailand_Roi_Et | Dog | 460LAm | 2002 | 103.77, 15.92 |
| AY849193 | Thailand_Roi_Et | Dog | 694LAsp | 2002 | 103.77, 15.92 |
| AY849198 | Thailand_Roi_Et | Dog | 695LAck | 2002 | 103.77, 15.92 |
| AY849182 | Thailand_Roi_Et | Dog | 458LAm | 2001 | 103.77, 15.92 |
| AY849208 | Thailand_Sakon_Nakhon | Dog | 711SLm | 2000 | 103.83, 17.37 |
| AY580093 | Thailand_Samut_Prakan | Dog | 1SPpsd | 2001 | 100.71, 13.61 |
| AY580096 | Thailand_Samut_Prakan | Dog | 53SPppd | 1999 | 100.71, 13.61 |
| AY580115 | Thailand_Samut_Prakan | Dog | 14SPppd | 2001 | 100.71, 13.61 |
| AY580120 | Thailand_Samut_Prakan | Dog | 23SPppd | 2001 | 100.71, 13.61 |
| AY580128 | Thailand_Samut_Prakan | Dog | 62SPbp | 1999 | 100.71, 13.61 |
| AY580132 | Thailand_Samut_Prakan | Dog | 211SPbp | 1999 | 100.71, 13.61 |
| AY849024 | Thailand_Samut_Prakan | Dog | 53SPppd | 1999 | 100.71, 13.61 |
| AY849067 | Thailand_Samut_Prakan | Dog | 23SPppd | 2001 | 100.71, 13.61 |
| AY849072 | Thailand_Samut_Prakan | Dog | 62SPbp | 1999 | 100.71, 13.61 |
| AY849133 | Thailand_Samut_Prakan | Dog | 86SPm | 2001 | 100.71, 13.61 |
| AY849226 | Thailand_Samut_Prakan | Dog | 131SPpsj | 1999 | 100.71, 13.61 |
| AY580100 | Thailand_Samut_Sakhon | Dog | 125SSktb | 1999 | 100.21, 13.57 |
| AY580101 | Thailand_Samut_Sakhon | Dog | 133SSm | 1999 | 100.21, 13.57 |
| AY580102 | Thailand_Samut_Sakhon | Dog | 176SSktb | 1999 | 100.21, 13.57 |
| AY580103 | Thailand_Samut_Sakhon | Dog | 187SSm | 1999 | 100.21, 13.57 |
| AY580104 | Thailand_Samut_Sakhon | Dog | 207SSm | 1999 | 100.21, 13.57 |
| AY580105 | Thailand_Samut_Sakhon | Dog | 217SSm | 1999 | 100.21, 13.57 |
| AY849030 | Thailand_Samut_Sakhon | Dog | 125SSktb | 1999 | 100.21, 13.57 |
| AY849031 | Thailand_Samut_Sakhon | Dog | 133SSm | 1999 | 100.21, 13.57 |
| AY849032 | Thailand_Samut_Sakhon | Dog | 176SSktb | 1999 | 100.21, 13.57 |
| AY849033 | Thailand_Samut_Sakhon | Dog | 187SSm | 1999 | 100.21, 13.57 |
| AY849034 | Thailand_Samut_Sakhon | Dog | 207SSm | 1999 | 100.21, 13.57 |
| AY849035 | Thailand_Samut_Sakhon | Dog | 217SSm | 1999 | 100.21, 13.57 |
| AY849138 | Thailand_Samut_Songkhram | Dog | 316SMm | 2001 | 99.94, 13.40 |
| AY218996 | Thailand_Samutpraharn | Dog | D23 |  | 100.75,13.67 |
| AY849118 | Thailand_Satun | Dog | 406STm | 2002 | 99.95, 6.87 |
| AY849150 | Thailand_Satun | Dog | 584TRkt | 2001 | 99.95, 6.87 |
| AY849158 | Thailand_Satun | Dog | 608SRm | 2001 | 99.95, 6.87 |
| AY849180 | Thailand_Satun | Dog | 553STdkl | 2000 | 99.95, 6.87 |
| AY849181 | Thailand_Satun | Dog | 666STm | 2002 | 99.95, 6.87 |
| AY849159 | Thailand_Si_Sa_Ket | Human | HMS152S | 2001 | 104.42, 14.89 |
| AY849188 | Thailand_Si_Sa_Ket | Dog | 489SEkh | 2000 | 104.42, 14.89 |
| AY849209 | Thailand_Si_Sa_Ket | Dog | 709SEktr | 2000 | 104.42, 14.89 |
| AY849089 | Thailand_Sing_Buri | Dog | 376SHib | 2001 | 100.34, 14.94 |
| AY849092 | Thailand_Sing_Buri | Dog | 384SHib | 2001 | 100.34, 14.94 |
| AY849086 | Thailand_Songkla | Dog | 412SKsd | 2002 | 100.59, 6.85 |
| AY849087 | Thailand_Songkla | Dog | 414SKm | 2002 | 100.59, 6.85 |
| AY849099 | Thailand_Songkla | Dog | 408SKhy | 2002 | 100.59, 6.85 |
| AY849106 | Thailand_Songkla | Dog | 413SKhy | 2001 | 100.59, 6.85 |
| AY849111 | Thailand_Songkla | Dog | 400SKm | 2001 | 100.59, 6.85 |
| AY849236 | Thailand_Sukhothai | Dog | 801SUsk | 2002 | 99.72, 17.26 |
| AY849238 | Thailand_Sukhothai | Dog | 811SUkrm | 2002 | 99.72, 17.26 |
| AY849251 | Thailand_Sukhothai | Dog | 794SUssr | 2003 | 99.72, 17.26 |
| AY849252 | Thailand_Sukhothai | Dog | 784SUm | 2003 | 99.72, 17.26 |
| AY849253 | Thailand_Sukhothai | Dog | 785SUkkl | 2003 | 99.72, 17.26 |
| AY849254 | Thailand_Sukhothai | Dog | 788SUsk | 2003 | 99.72, 17.26 |
| AY849255 | Thailand_Sukhothai | Dog | 808SUssn | 2002 | 99.72, 17.26 |
| AY849256 | Thailand_Sukhothai | Dog | 793SUsn | 2003 | 99.72, 17.26 |
| AY849100 | Thailand_Suphan_Buri | Dog | 332SBm | 2001 | 100.00, 14.59 |
| AY849108 | Thailand_Suphan_Buri | Dog | 317SBspn | 2001 | 100.00, 14.59 |
| AY849110 | Thailand_Suphan_Buri | Dog | 340SBspn | 2001 | 100.00, 14.59 |
| AY849129 | Thailand_Suphan_Buri | Dog | 151SBdc | 1999 | 100.00, 14.59 |
| AY849179 | Thailand_Surat_Thani | Dog | 503SNws | 2002 | 99.06, 8.99 |
| AY849183 | Thailand_Surat_Thani | Dog | 500SNks | 2002 | 99.06, 8.99 |
| AY849190 | Thailand_Surat_Thani | Dog | 502SNcb | 2002 | 99.06, 8.99 |
| AY849191 | Thailand_Surat_Thani | Dog | 499SNps | 2002 | 99.06, 8.99 |
| AY849161 | Thailand_Surin | Dog | 473BRpk | 2001 | 103.70, 14.90 |
| AY849169 | Thailand_Surin | Dog | 463SRsn | 2001 | 103.70, 14.90 |
| AY849170 | Thailand_Surin | Dog | 464SRskp | 2001 | 103.70, 14.90 |
| AY849171 | Thailand_Surin | Dog | 465SRm | 2001 | 103.70, 14.90 |
| AY849172 | Thailand_Surin | Dog | 466SRrbr | 2001 | 103.70, 14.90 |
| AY849144 | Thailand_Trang | Dog | 507TRrd | 2000 | 99.58, 7.57 |
| AY849148 | Thailand_Trang | Dog | 559TRpl | 2000 | 99.58, 7.57 |
| AY849160 | Thailand_Trang | Dog | 599TRhy | 2001 | 99.58, 7.57 |
| AY849204 | Thailand_Ubol_Ratchathani | Dog | 726UBbt | 2001 | 105.10, 15.21 |
| AY849211 | Thailand_Ubol_Ratchathani | Dog | 728UBm | 2001 | 105.10, 15.21 |
| AY849217 | Thailand_Ubol_Ratchathani | Dog | 731UBvrc | 2001 | 105.10, 15.21 |
| AY849222 | Thailand_Ubol_Ratchathani | Dog | 744UBdud | 2002 | 105.10, 15.21 |
| AY849224 | Thailand_Ubol_Ratchathani | Dog | 717UBm | 2001 | 105.10, 15.21 |
| AY849079 | Thailand_Uthai_Thani | Dog | 355UThk | 2001 | 99.49, 15.33 |
| AY849090 | Thailand_Uthai_Thani | Dog | 380UTbr | 2001 | 99.49, 15.33 |
| AY849096 | Thailand_Uthai_Thani | Dog | 357UTm | 2001 | 99.49, 15.33 |
| AY849097 | Thailand_Uthai_Thani | Dog | 356UTth | 2001 | 99.49, 15.33 |
| AY849240 | Thailand_Uttaradit | Dog | 814UDm | 2002 | 100.47, 17.72 |
| AY849241 | Thailand_Uttaradit | Dog | 807UDts | 2002 | 100.47, 17.72 |
| AY849242 | Thailand_Uttaradit | Dog | 800Udm | 2002 | 100.47, 17.72 |
| AY849259 | Thailand_Uttaradit | Dog | 804UDm | 2002 | 100.47, 17.72 |
| AY849135 | Thailand_Yala | Dog | 396YLm | 2001 | 101.23, 6.22 |
| AY849152 | Thailand_Yala | Dog | 595Ylbns | 2001 | 101.23, 6.22 |
| AY849157 | Thailand_Yala | Dog | 589YLm | 2001 | 101.23, 6.22 |
| AY849202 | Thailand_Yala | Dog | 568YLrm | 2000 | 101.23, 6.22 |
| AY849201 | Thailand_Yasothon | Dog | 708YSm | 2000 | 104.31, 15.94 |
| AY849213 | Thailand_Yasothon | Dog | 714YSm | 2001 | 104.31, 15.94 |
| AY849214 | Thailand_Yasothon | Dog | 715YSm | 2001 | 104.31, 15.94 |
| AY849215 | Thailand_Yasothon | Dog | 718YSlkk | 2001 | 104.31, 15.94 |
| AY849221 | Thailand_Yasothon | Dog | 741YSm | 2002 | 104.31, 15.94 |
| EU086210 | VietNam_Ho_Chi_Minh | Dog | 01017VNM | 2001 | 106.64, 10.82 |
| EU086209 | VietNam_Lai_Thieu | Dog | 01016VNM | 2001 | 106.70, 10.90 |
