# Supplementary material for: National Borders Effectively Halt the Spread of Rabies: The Current Rabies Epidemic in China Is Dislocated from Cases in Neighboring Countries
Source: PLoS Negl Trop Dis. 2013 Jan 31;7(1):e2039. doi: 10.1371/journal.pntd.0002039 (PMC3561166; doi:10.1371/journal.pntd.0002039)
Supplement: Table S2 — Summary of datasets used in this study. (DOC) [file pntd.0002039.s004.doc]

**Table S2. Summary of datasets used in this study**


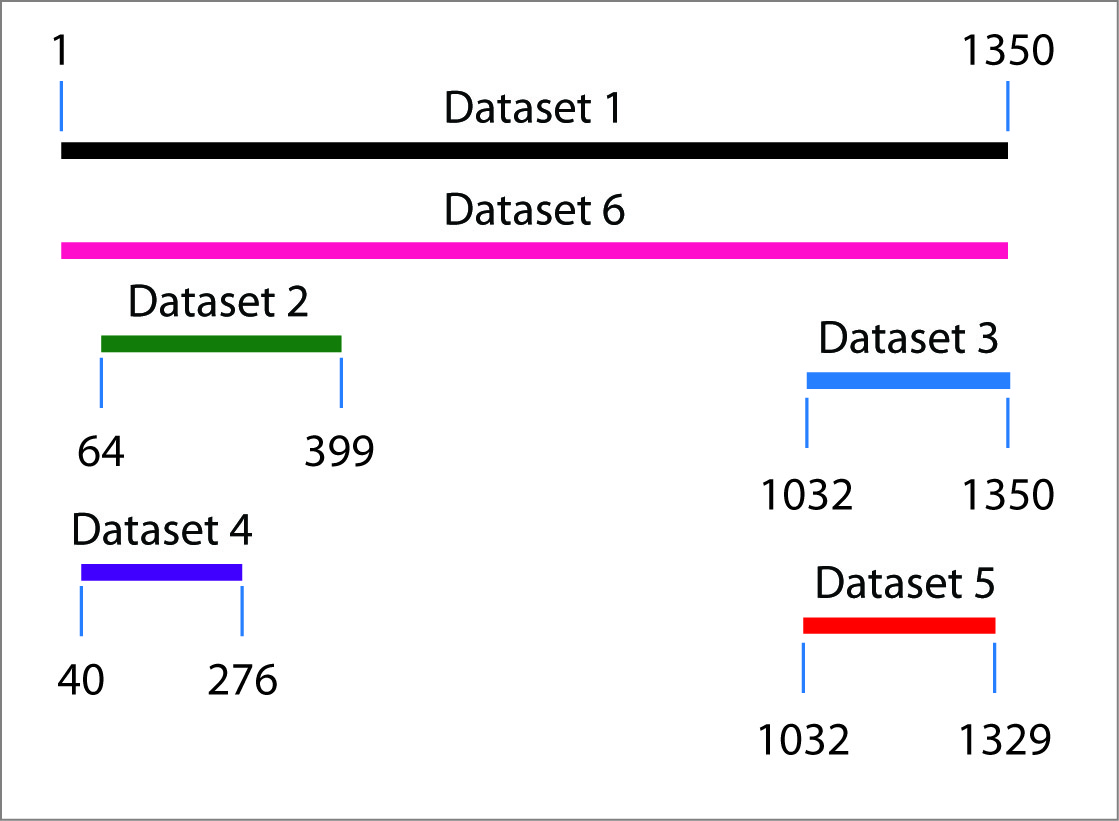


| **Country** | **Dataset 1** | **Dataset 2** | **Dataset 3** |
| --- | --- | --- | --- |
| Afghanistan | 2 | 2 | 5 |
| Bhutan |  | 7 |  |
| Cambodia | 4 | 4 | 4 |
| China | 54 | 80 | 78 |
| India | 5 | 11 | 37 |
| Indonesia | 1 | 14 | 33 |
| Japan | 2 | 3 | 3 |
| Kazakhstan | 2 | 4 | 4 |
| Laos | 2 | 2 | 2 |
| Mongolia | 4 | 10 | 8 |
| Myanmar | 3 | 3 | 3 |
| Nepal | 1 | 3 | 3 |
| Pakistan | 2 | 2 | 3 |
| Philippines | 4 | 8 | 35 |
| Russia | 6 | 21 | 47 |
| South Korea | 5 | 9 | 11 |
| Sri Lanka | 4 | 8 | 9 |
| Thailand | 7 | 15 | 20 |
| Vietnam | 2 | 2 | 8 |
| Total | 110 | 208 | 313 |

| **dataset 4 sequence range N40-N276** | | |  |
| --- | --- | --- | --- |
|  |  |  |  |
| **Country** | **No Of Seqs** |  |  |
|  |  |  |  |
| Viet Nam | 38 |  |  |
| Cambodia | 7 |  |  |
| Laos | 4 |  |  |
| Myanmar | 3 |  |  |
| Philippines | 72 |  |  |
| Thailand | 74 |  |  |
| China | 93 | Guangxi | 36 |
|  |  | Yunnan | 13 |
|  |  | Guizhou | 14 |
|  |  | Guangdong | 2 |
|  |  | Henan | 3 |
|  |  | Hunan | 1 |
|  |  | Zhejiang | 2 |
|  |  | Shanghai | 4 |
|  |  | Ningxia | 1 |
|  |  | Jiangsu | 4 |
|  |  | Jiangxi | 2 |
|  |  | Shandong | 1 |
|  |  | Sichuan | 3 |
|  |  | Jilin | 1 |
|  |  | Anhui | 5 |
|  |  | Chongqing | 1 |

| **dataset 5 sequence range N1033-1329** | | | |
| --- | --- | --- | --- |
|  |  |  |  |
| **Country** | **No Of Seqs** |  |  |
|  |  |  |  |
| Viet Nam | 2 |  |  |
| Cambodia | 6 |  |  |
| Laos | 3 |  |  |
| Myanmar | 3 |  |  |
| Philippines | 6 |  |  |
| Thailand | 332 |  |  |
| China | 78 | Guangxi | 29 |
|  |  | Yunnan | 13 |
|  |  | Guizhou | 12 |
|  |  | Hunan | 3 |
|  |  | Zhejiang | 3 |
|  |  | Shanghai | 5 |
|  |  | Jiangsu | 2 |
|  |  | Jiangxi | 2 |
|  |  | Sichuan | 2 |
|  |  | Anhui | 5 |
|  |  | Chongqing | 1 |
|  |  | Jilin | 1 |

| **Province** | **Dataset 6** |
| --- | --- |
| Anhui | 22 |
| Chongqing | 2 |
| Fujian | 12 |
| Guangdong | 2 |
| Guangxi | 36 |
| Guizhou | 16 |
| Hebei, Beijing | 7 |
| Henan | 9 |
| Hubei | 3 |
| Hunan | 27 |
| Inner Mongolia | 3 |
| Jiangsu | 17 |
| Jiangxi | 9 |
| Jilin | 1 |
| Ningxia | 2 |
| Shaanxi | 2 |
| Shandong | 11 |
| Shanghai | 13 |
| Sichuan | 9 |
| Yunnan | 14 |
| Zhejiang | 15 |
| Total | 232 |
