# Supplementary material for: National Borders Effectively Halt the Spread of Rabies: The Current Rabies Epidemic in China Is Dislocated from Cases in Neighboring Countries
Source: PLoS Negl Trop Dis. 2013 Jan 31;7(1):e2039. doi: 10.1371/journal.pntd.0002039 (PMC3561166; doi:10.1371/journal.pntd.0002039)
Supplement: Table S3 — Number of China I and China II isolates collected by Trial National Rabies Surveillance Program. (DOC) [file pntd.0002039.s005.doc]

**Table S3. Number of China I and China II isolates by Trial National Rabies Surveillance Program.**

Number of China I and China II isolates collected in China by year as part of Trial National Rabies Surveillance Program. As rabies has spread through China and the surveillance area has expanded, the number of China I isolates has increased while the number of China II isolates has decreased. Furthermore, China II isolates are still only found in the southwestern provinces of China. Thus, China I is now the dominant lineage in the current epidemic

| **year** | **I** | **II** |
| --- | --- | --- |
| 2003 | 6 | 16 |
| 2004 | 61 | 16 |
| 2005 | 71 | 31 |
| 2006 | 42 | 22 |
| 2007 | 34 | 13 |
| 2008 | 76 | 33 |
| 2009 | 43 | 2 |
| 2010 | 21 | 0 |
